# Aryl nitrile oxide cycloaddition reactions in the presence of pinacol boronic acid ester

**DOI:** 10.3762/bjoc.8.67

**Published:** 2012-04-19

**Authors:** Sarah L Harding, Sebastian M Marcuccio, G Paul Savage

**Affiliations:** 1 CSIRO Materials Science and Engineering, Private Bag 10, Clayton South MDC, Vic 3169, Australia; 2 Advanced Molecular Technologies Pty Ltd, Unit 1, 7–11 Rocco Drive, Scoresby, VIC 3179, Australia

**Keywords:** dipolar cycloaddition, heterocycle, nitrile oxide, hypervalent iodine oxidation, pinacol boronic acid esters

## Abstract

An aryl substrate with dual functionality consisting of a nitrile oxide and a pinacolyl boronate ester was prepared by mild hypervalent iodine oxidation (diacetoxyiodobenzene) of the corresponding aldoxime, without decomposition of the boronate functionality. The nitrile oxide was trapped in situ with a variety of dipolarophiles to yield aryl isoxazolines with the boronate ester function intact and available for subsequent reaction.

## Introduction

**Scheme 1:**
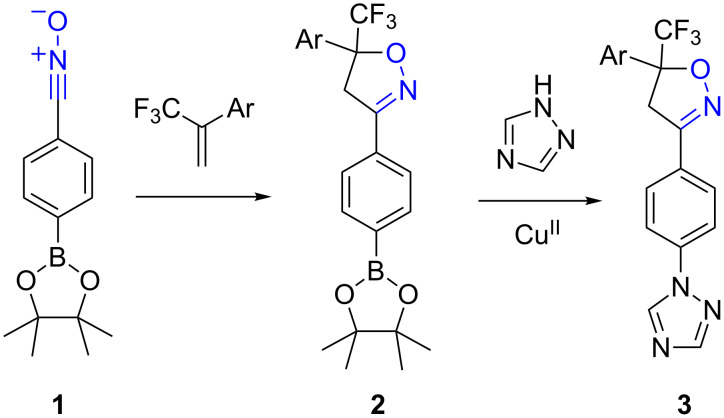
Concept for library generation by dipolar cycloaddition followed by boronate coupling.

Metal-mediated coupling reactions to form carbon–carbon bonds, and 1,3-dipolar cycloaddition reactions to construct five-membered heterocycles are both powerful tools for assembling organic molecules. Used in combination, these tools offer great flexibility for strategies such as diversity-oriented synthesis [1], solution-phase combinatorial libraries [2], and continuous-flow processes [3–4]. An important consideration when using these reactions for multistep syntheses is whether they are chemically compatible, without having to resort to protection/deprotection systems. For example, to generate a library of 3-bi(hetero)aryl isoxazoline analogues **3** a convenient substrate would be the arylboronate nitrile oxide **1**, which would undergo 1,3-dipolar cycloaddition to give isoxazolines **2**. This latter compound could in turn be coupled with heterocycles or aryl groups to give insecticidal [5] derivatives of type **3** (Scheme 1).

The utility of arylboronic acids and esters in organic synthesis is demonstrated by their use as key intermediates in transition-metal-catalysed bond-forming reactions [6], which include the Miyaura–Suzuki coupling reaction [7], copper-catalysed heteroatom arylation [8], allylboration [9], and the Petasis reaction [10]. Aryl boronic esters also undergo many of these coupling reactions and Miyaura’s protocol for the palladium-catalysed cross-coupling of bis(pinacolato)diboron with aryl and vinyl halides or triflates has become one of the most popular methods for preparing arylboronic esters under mild conditions [11]. The resulting pinacolyl boronate esters have the advantage of being stable, readily handled compounds.

**Scheme 2:**
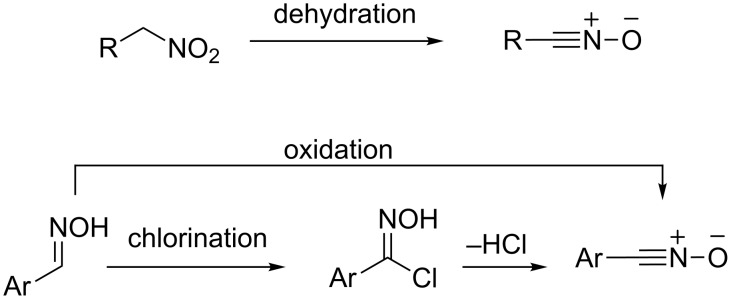
General formation of alkyl (R) and aryl (Ar) nitrile oxides.

The Huisgen 1,3-dipolar cycloaddition reaction is a powerful and versatile method for constructing five-membered heterocycles [12–14]. Nitrile oxide 1,3-dipoles react with carbon–carbon dipolarophiles, such as alkenes [15], alkynes [16–17], and benzyne [18–19], to give Δ^2^-isoxazolines and isoxazoles. These are interesting sources of bioactive compounds in their own right, but isoxazoles are particularly valuable for their latent functionality as β-hydroxyketones, β-aminoalcohols, 1,3-diols, and a range of other 1,3-disubstituted compounds, through N–O bond cleavage [20]. Nitrile oxides are reactive intermediates that are usually generated in situ and react immediately with the dipolarophile. There have been many methods reported for the generation of nitrile oxides, but the most common one for alkyl nitrile oxides involves the dehydration of primary nitro compounds [21]. Aryl nitrile oxides are more commonly prepared by chlorination of aldoximes followed by dehydrohalogenation of the resulting hydroximoyl chlorides, or by direct oxidative dehydrogenation of the aldoximes (Scheme 2) [17].

While the halogenation–dehydrohalogenation process is most common, several methods involving direct oxidative dehydrogenation of aldoximes have been reported, including the use of lead tetraacetate [22–23], mercury(II) acetate [24], hypervalent iodine [25–26], and manganese(IV) oxide [27]. We were interested in developing a mild method for the introduction of a nitrile oxide functionality in the presence of an arylboronic ester, allowing subsequent elaboration. We herein report the synthesis and 1,3-dipolar cycloaddition reactions of 4-pinacolatoboron benzonitrile oxide **1**.

## Results and Discussion

Several reports of 1,3-dipolar cycloaddition reactions of nitrile oxides to vinylboronate esters [28–29] and alkynylboronate esters [30–31] have recently appeared. In each case the nitrile oxide was either isolated first (this procedure is limited to hindered nitrile oxides, such as 2,4,6-trimethylbenzonitrile oxide) or generated in situ by dehydrohalogenation of a preformed hydroximoyl chloride. This is presumably to avoid the competing oxidative side reactions that would be expected at the boronate ester if the nitrile oxide were generated oxidatively from the aldoxime [32]. The same thermodynamic bias favouring the oxidation of carbon–boron bonds, which makes boronic ester chemistry chemoselective, is a constraint that potentially limits the utility of nitrile oxide cycloadditions in the presence of a boronic acid ester.

**Scheme 3:**
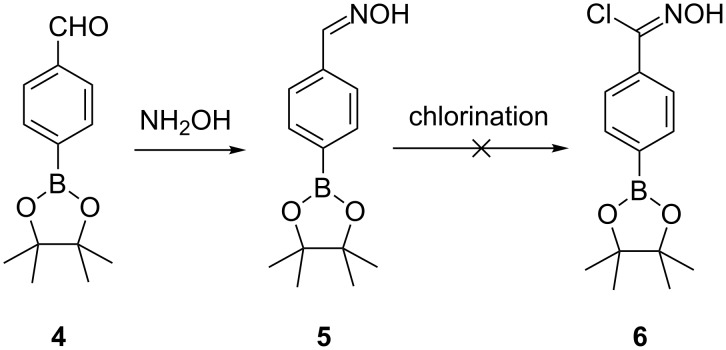
Formation of 4-(aldoxime)phenylboronic acid pinacol ester **5**.

4-Formylphenylboronic acid pinacol ester **4** is commercially available or easily prepared from the corresponding boronic acid, via the bromide [33]. Reaction with 50% aqueous hydroxylamine gives the aldoxime **5** in good yield (Scheme 3). Only one geometric isomer of the aldoxime was observed in the ^1^H and ^13^C NMR spectra, and this was assigned as the *Z* isomer based on the 8.17 ppm chemical shift of the C(H)=N proton [34]. Attempted methods for the chlorination of aryl aldoximes to aryl hydroximoyl chloride include the use of *N*-chlorosuccinimide [35], chloramine-T [36], biphasic sodium hypochlorite [37–38], and *tert*-butyl hypochlorite [39]. These methods led to either no reaction or, when forced, decomposition of the intermediate **5** with no detectible hydroximoyl chloride **6**. Given that these reagents are oxidants such a result is perhaps not surprising.

We then turned our attention to the direct oxidation of aldoximes using the mild conditions of hypervalent iodine oxidation and were pleased to discover that diacetoxyiodobenzene (DIB) [26,40] could cleanly convert the aldoxime to the corresponding nitrile oxide without decomposition of the boronate ester function. The nitrile oxide was trapped in situ with a variety of dipolarophiles to yield isoxazolines (Table 1).

**Table 1:** Cycloaddition of 4-pinacolatoboron benzonitrile oxide **1** with dipolarophiles.

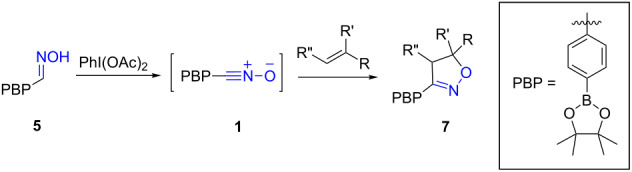			

entry	dipolarophile	product (**7**)	purified yield (%)

a	styrene	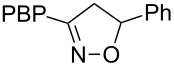	74
b	methyl acrylate	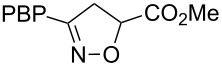	59
c	*tert*-butyl acrylate	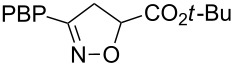	56
d	*N,N*-dimethylacrylamide	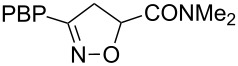	49
e	methyl methacrylate	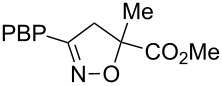	82
f	1-heptene	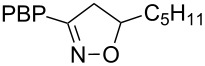	36
g	α-methylstyrene	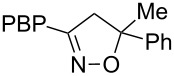	48
h	*trans* β-methylstyrene	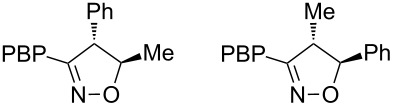 1:2	36
i	4-bromostyrene	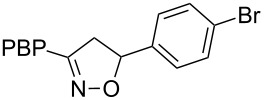	71
j	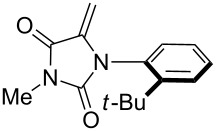	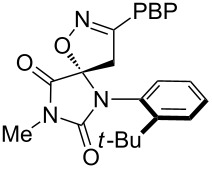	48

For mono-substituted or 1,1-disubstituted alkenes the regiochemistry of the nitrile oxide cycloaddition followed the expected outcome in which the oxygen of the nitrile oxide became attached to the more substituted end of the double bond [41]. This regiochemical orientation was established from the ^1^H NMR chemical shifts for the cycloadduct isoxazoline ring protons. The resonances of protons on C4 of the isoxazoline ring appear 1–2 ppm upfield from those of C5 protons on the isoxazoline rings [42], and hence 5-substituted isoxazolines are easily distinguished from 4-substituted isoxazolines. All of the monosubstituted and 1,1-disubstituted alkenes led to 5-substituted and 5,5-disubstituted isoxazolines, respectively. In the case of *trans*-β-methylstyrene (Table 1, entry h), an inseparable mixture of regioisomers was obtained in a 2:1 ratio favouring the addition of the nitrile oxide oxygen to the phenyl-substituted end of the carbon–carbon double bond. This is consistent with previously reported benzonitrile oxide cycloaddition reactions with *trans*-β-methylstyrene [43]. For both regioisomers the coupling between the C4 and C5 protons (approximately 5–6 Hz) indicated a retention of the configuration of the trans geometry in the cycloadduct, which is consistent with the concerted nature of the 1,3-dipolar cycloaddition reaction.

For the cycloaddition reaction with the hydantoin compound, 3-methyl-1-(2-*tert*-butylphenyl)-5-methyleneimidazol-2,4-dione (Table 1, entry j), only a single diastereomer was detected. We have previously observed that nitrile oxide cycloadditions to this hydantoin and related compounds can show facial selectivity based on atropisomerism around the *N*-aryl bond [38,44]. With benzonitrile oxide the facial selectivity was 30:1 favouring addition *anti* to the *tert*-butyl group; however, with the boronate ester benzonitrile oxide **5** only the *anti* cycloadduct was isolated and no *syn* cycloadduct was detected.

## Conclusion

Aryl nitrile oxides can be prepared oxidatively in the presence of boronate esters by using the hypervalent iodine reagent, diacetoxyiodobenzene. Nitrile oxides prepared in this way undergo 1,3-dipolar cycloaddition to yield substituted isoxazolines.

## Experimental

### General experimental procedures

Melting points were determined on a Büchi B-545 instrument and are uncorrected. ^1^H and ^13^C NMR spectra were recorded on a Bruker AV400 spectrometer at 400 and 100 MHz, respectively, by using CDCl_3_ as solvent and internal reference. Electron impact (EI) mass spectra were run on a ThermoQuest MAT95XP mass spectrometer with an ionization energy of 70 eV. Accurate mass measurements were obtained on the same instrument with a resolution of 5000–10000 by using perfluorokerosene (PFK) as the reference compound. Accurate masses were measured on the ^11^B ions.

**(*Z*)-4-(4,4,5,5-tetramethyl-1,3,2-dioxaborolan-2-yl)benzaldehyde oxime (5)**: To a stirred solution of 4-formylphenylboronic acid pinacol ester **4** (100 g, 0.43 mol) in diethyl ether (400 mL) was added 50% hydroxylamine in H_2_O (25.9 mL, 0.43 mol) in one portion. The reaction mixture immediately became warm and was stirred for a further 10 min, then dried (MgSO_4_) and filtered, and the ether was removed under reduced pressure to yield a pale yellow oil (95 g, 89%) that crystallized upon standing. A sample of the crude material was kept as a slurry in hexane (140 mL) overnight, collected by filtration and dried at room temperature in a vacuum oven, mp 115–117 °C; ^1^H NMR (CDCl_3_, 400 MHz) δ 8.22 (s, 1H), 8.17 (s, 1H), 7.84 (d, *J* = 8.0 Hz, 2H), 7.59 (d, *J* = 8.0 Hz, 2H), 1.35 (s, 12H); ^13^C NMR (CDCl_3_, 100 MHz) δ 150.3, 135.1, 134.4, 126.2, 84.0, 24.8; HRMS–EI (*m*/*z*): calcd for C_13_H_18_BNO_3_, 247.1374; found, 247.1373.

### General procedure for cycloaddition reactions

To a stirred solution of the appropriate dipolarophile (0.55 mmol) and diacetoxyiodobenzene (177 mg, 0.55 mmol) in methanol (5 mL), at 0 °C, was added 4-(4,4,5,5-tetramethyl-1,3,2-dioxaborolan-2-yl)benzaldehyde oxime (**5**) (125 mg, 0.5 mmol) in methanol (3 mL), dropwise over 10 min followed by three drops of trifluoroacetic acid. The pale yellow solution was allowed to warm to room temperature and stirred for 2 h then concentrated under reduced pressure. The residue was purified by column chromatography on silica as stated.

**5-Phenyl-3-(4-(4,4,5,5-tetramethyl-1,3,2-dioxaborolan-2-yl)phenyl)-4,5-dihydroisoxazole (7a)**: Isolated as a white solid (130 mg, 74%) after purification by column chromatography (20% Et_2_O in petroleum ether, *R*_f_ 0.41, 50% Et_2_O in petroleum ether); ^1^H NMR (CDCl_3_, 400 MHz) δ 7.85 (d, *J* = 8.1 Hz, 2H), 7.69 (d, *J* = 8.1 Hz, 2H), 7.42–7.29 (m, 5H), 5.75 (dd, *J* = 11.0, 8.4 Hz, 1H), 3.79 (dd, *J* = 16.6, 11.0 Hz, 1H), 3.35 (dd, *J* = 16.6, 8.4 Hz, 1H), 1.36 (s, 12H); ^13^C NMR (CDCl_3_, 100 MHz) δ 156.1, 140.7, 134.95, 131.7, 128.65, 128.1, 125.8, 125.7, 83.8, 82.6, 42.9, 24.75; IR (KBr) ν/cm^−1^: 2979 (w), 1755 (w), 1607 (w), 1396 (m), 1358 (st), 1325 (m), 1269 (m), 1142 (st); HRMS–EI (*m*/*z*): calcd for C_21_H_24_BNO_3_, 349.1844; found, 349.1839.

**Methyl 3-(4-(4,4,5,5-tetramethyl-1,3,2-dioxaborolan-2-yl)phenyl)-4,5-dihydroisoxazole-5-carboxylate (7b)**: Isolated as a white solid (98 mg, 59%) after purification by column chromatography (20% Et_2_O in petroleum ether, *R*_f_ 0.18, 50% Et_2_O in petroleum ether); ^1^H NMR (CDCl_3_, 400 MHz) δ 7.83 (d, *J* = 8.2 Hz, 2H), 7.65 (d, *J* = 8.2 Hz, 2H), 5.19 (dd, *J* = 10.8, 7.5 Hz, 1H), 3.81 (s, 3H), 3.79 (m, 2H), 1.34 (s, 12H); ^13^C NMR (CDCl_3_, 100 MHz) δ 170.6, 156.1, 135.1, 130.8, 126.05, 84.05, 78.0, 52.8, 38.8, 24.8; IR (KBr) ν/cm^−1^: 2916 (w), 1761 (m), 1607 (w), 1392 (m), 1353 (st), 1325 (m), 1268 (m), 1208 (m), 1140 (st); HRMS–EI (*m*/*z*): calcd for C_17_H_22_BNO_5_, 331.1586; found, 331.1588.

***tert*-Butyl 3-(4-(4,4,5,5-tetramethyl-1,3,2-dioxaborolan-2-yl)phenyl)-4,5-dihydroisoxazole-5-carboxylate (7c)**: Isolated as a white solid (105 mg, 56%) after purification by column chromatography (10% Et_2_O in petroleum ether); ^1^H NMR (CDCl_3_, 400 MHz) δ 7.78 (d, *J* = 8.2 Hz, 2H), 7.64 (d, *J* = 8.2 Hz, 2H), 5.03 (t, *J* = 9.4 Hz, 1H), 3.56 (d, *J* = 9.4 Hz, 2H), 1.47 (s, 9H), 1.32 (s, 12H); ^13^C NMR (CDCl_3_, 100 MHz) δ 169.25, 156.1, 135.2, 131.3, 126.2, 84.2, 82.9, 79.0, 38.8, 28.1, 25.0; IR (KBr) ν/cm^−1^: 2966 (m), 1742 (m), 1726 (m), 1611 (w), 1390 (m), 1356 (st), 1323 (m), 1142 (st); HRMS–EI (*m*/*z*): calcd for C_20_H_28_BNO_5_, 373.2055; found, 373.2048.

***N,N*-dimethyl-3-(4-(4,4,5,5-tetramethyl-1,3,2-dioxaborolan-2-yl)phenyl)-4,5-dihydroisoxazole-5-carboxamide (7d)**: Isolated as a colourless oil (85 mg, 49%) after purification by column chromatography (80% Et_2_O in petroleum ether, *R*_f_ 0.43, 50% Et_2_O in petroleum ether); ^1^H NMR (CDCl_3_, 400 MHz) δ 7.82 (d, *J* = 8.3 Hz, 2H), 7.67 (d, *J* = 8.2 Hz, 2H), 5.38 (dd, *J* = 11.2, 7.7 Hz, 1H), 4.19 (dd, *J* = 16.8, 7.7 Hz, 1H), 3.38 (dd, *J* = 16.8, 11.3 Hz, 1H), 3.21 (s, 3H), 3.00 (s, 3H), 1.34 (s, 12H); ^13^C NMR (CDCl_3_, 100 MHz) δ 167.2, 157.4, 135.0, 131.3, 126.0, 84.0, 78.3, 37.3, 36.8, 36.1, 24.8; IR (KBr) ν/cm^−1^: 2966 (m), 1742 (m), 1726 (m), 1611 (w), 1390 (m), 1356 (st), 1323 (m), 1142 (st); HRMS–EI (*m*/*z*): calcd for C_18_H_25_BN_2_O_4_, 344.1902; found, 344.1899.

**Methyl 5-methyl-3-(4-(4,4,5,5-tetramethyl-1,3,2-dioxaborolan-2-yl)phenyl)-4,5-dihydroisoxazole-5-carboxylate (7e)**: Isolated as a pale yellow oil (141 mg, 82%) after purification by column chromatography (15% Et_2_O in petroleum ether, *R*_f_ 0.36, 50% Et_2_O in petroleum ether); ^1^H NMR (CDCl_3_, 400 MHz) δ 7.81 (d, *J* = 8.1 Hz, 2H), 7.63 (d, *J* = 8.2 Hz, 2H), 3.88 (d, *J* = 17.0 Hz, 1H), 3.79 (s, 3H), 3.21 (d, *J* = 17.0 Hz, 1H), 1.70 (s, 3H), 1.33 (s, 12H); ^13^C NMR (CDCl_3_, 100 MHz) δ 172.5, 156.3, 135.0, 131.3, 125.85, 86.2, 84.0, 53.0, 44.65, 24.8, 23.6; IR (KBr) ν/cm^−1^: 2975 (w), 1744 (m), 1609 (w), 1516 (w), 1394 (m), 1349 (st), 1326 (st), 1268 (m), 1146 (st); HRMS–EI (*m*/*z*): calcd for C_18_H_24_BNO_5_, 345.1742; found, 345.1740.

**5-Pentyl-3-(4-(4,4,5,5-tetramethyl-1,3,2-dioxaborolan-2-yl)phenyl)-4,5-dihydroisoxazole (7f)**: Isolated as a colourless oil (61 mg, 36%) after purification by column chromatography (20% Et_2_O in petroleum ether, *R*_f_ 0.40, 50% Et_2_O in petroleum ether); ^1^H NMR (CDCl_3_, 400 MHz) δ 8.32 (d, *J* = 8.3 Hz, 2H), 7.50 (m, 4H), 7.31 (ddd, *J* = 8.2, 7.2, 1.6 Hz, 1H), 7.19 (ddd, *J* = 7.9, 7.3, 1.5 Hz, 1H), 3.87 (d, *J* = 17.8 Hz, 1H), 3.37 (d, *J* = 17.5 Hz, 1H), 3.20 (s, 3H), 1.36 (s, 9H), 1.32 (s, 12H); ^13^C NMR (CDCl_3_, 100 MHz) δ 156.4, 134.95, 132.3, 125.7, 83.95, 81.6, 39.8, 35.3, 31.6, 25.15, 24.8, 22.5, 13.95; IR (KBr) ν/cm^−1^: 2929 (w), 1611 (w), 1397 (m), 1357 (st), 1323 (m), 1268 (m), 1142 (st), 1091 (st), 858 (m); HRMS–EI (*m*/*z*): calcd for C_20_H_30_BNO_3_, 343.2313; found, 343.2311.

**5-Methyl-5-phenyl-3-(4-(4,4,5,5-tetramethyl-1,3,2-dioxaborolan-2-yl)phenyl)-4,5-dihydroisoxazole (7g)**: Isolated as a colourless oil (88 mg, 48%) after purification by column chromatography (10% Et_2_O in petroleum ether, *R*_f_ 0.54, 50% Et_2_O in petroleum ether); ^1^H NMR (CDCl_3_, 400 MHz) δ 7.80 (d, *J* = 8.3 Hz, 2H), 7.63 (d, *J* = 8.3 Hz, 2H), 7.47 (m, 2H), 7.35 (m, 2H), 7.27 (m, 1H), 3.49 (AB quartet, *J* = 16.5 Hz, 2H), 1.79 (s, 3H), 1.33 (s, 12H); ^13^C NMR (CDCl_3_, 100 MHz) δ 156.4, 145.6, 135.2, 132.4, 128.7, 127.5, 125.9, 124.8, 88.4, 84.2, 48.8, 28.5, 25.0; HRMS–EI (*m*/*z*): calcd for C_22_H_26_BNO_3_, 363.2000; found, 363.1979.

**4-Methyl-5-phenyl-3-(4-(4,4,5,5-tetramethyl-1,3,2-dioxaborolan-2-yl)phenyl)-4,5-dihydroisoxazole (7h_a_) and 5-methyl-4-phenyl-3-(4-(4,4,5,5-tetramethyl-1,3,2-dioxaborolan-2-yl)phenyl)-4,5-dihydroisoxazole (7h_b_)**: Isolated as a colourless oil (65 mg, 36%) after purification by column chromatography (10% Et_2_O in petroleum ether, *R*_f_ 0.57, 50% Et_2_O in petroleum ether) to give an inseparable 2:1 mixture of regioisomers **a** and **b**; ^1^H NMR (CDCl_3_, 400 MHz) regioisomer **a**, δ 7.84 (d, *J* = 8.3 Hz, 2H), 7.66 (d, *J* = 8.3 Hz, 2H), 7.39–7.28 (m, 5H), 5.31 (d, *J* = 5.6 Hz, 1H), 3.70 (dq, *J* = 7.1, 5.7 Hz, 1H), 1.45 (d, *J* = 7.1 Hz, 3H), 1.35 (s, 12H); ^1^H NMR (CDCl_3_, 400 MHz) regioisomer **b**, δ 7.70 (d, *J* = 8.3 Hz, 2H), 7.56 (d, *J* = 8.3 Hz, 2H), 7.25–7.19 (m, 5H), 4.66 (apparent qn, *J* = 6.3 Hz, 1H), 4.33 (d, *J* = 6.0 Hz, 1H), 1.49 (d, *J* = 6.3 Hz, 3H), 1.31 (s, 12H); ^13^C NMR (CDCl_3_, 100 MHz) resonances that could be assigned to specific regioisomers by using proton–carbon 2D correlation spectroscopy are designated a or b, δ 160.2, 158.1, 140.8, 139.0, 135.1, 134.8, 131.5, 131.2, 130.4, 129.2, 128.7, 128.1, 127.6, 127.45, 126.3, 126.2, 125.4, 90.15^a^, 86.95^b^, 84.0, 83.9, 61.0^b^, 50.75^a^, 24.8, 24.75, 20.5^b^, 18.2^a^; HRMS–EI (*m*/*z*): calcd for C_22_H_26_BNO_3_, 363.2000; found, 363.1988.

**5-(4-Bromophenyl)-3-(4-(4,4,5,5-tetramethyl-1,3,2-dioxaborolan-2-yl)phenyl)-4,5-dihydroisoxazole (7i)**: Isolated as a white solid (152 mg, 71%) after purification by column chromatography (10% Et_2_O in petroleum ether, *R*_f_ 0.35, 50% Et_2_O in petroleum ether); ^1^H NMR (CDCl_3_, 400 MHz) δ 7.82 (d, *J* = 8.3 Hz, 2H), 7.65 (d, *J* = 8.3 Hz, 2H), 7.48 (d, *J* = 8.5 Hz, 2H), 7.26 (d, *J* = 8.5 Hz, 2H), 5.68 (dd, *J* = 11.0, 8.2 Hz, 1H), 3.77 (dd, *J* = 16.7, 11.1 Hz, 1H), 3.28 (dd, *J* = 16.7, 8.2 Hz, 1H), 1.33 (s, 12H); ^13^C NMR (CDCl_3_, 400 MHz) δ 156.1, 139.9, 135.1, 131.9, 131.5, 127.55, 125.9, 122.1, 84.0, 81.9, 43.0, 24.85; HRMS–EI (*m*/*z*): calcd for C_21_H_23_BBrNO_3_, 427.0949; found, 427.0936.

**6-(2-*tert*-Butylphenyl)-8-methyl-3-(4-(4,4,5,5-tetramethyl-1,3,2-dioxaborolan-2-yl)phenyl)-1-oxa-2,6,8-triazaspiro[4.4]non-2-ene-7,9-dione (7j)**: Isolated as a white solid (121 mg, 48%) after purification by column chromatography (50% Et_2_O in petroleum ether, *R*_f_ 0.38, Et_2_O); ^1^H NMR (CDCl_3_, 400 MHz) δ 8.32 (d, *J* = 8.3 Hz, 2H), 7.50 (m, 4H), 7.31 (ddd, *J* = 8.2, 7.2, 1.6 Hz, 1H), 7.19 (ddd, *J* = 7.9, 7.3, 1.5 Hz, 1H), 3.87 (d, *J* = 17.5 Hz, 1H), 3.37 (d, *J* = 17.5 Hz, 1H), 3.20 (s, 3H), 1.36 (s, 9H), 1.32 (s, 12H); ^13^C NMR (CDCl_3_, 100 MHz) δ 169.1, 156.1, 155.4, 148.3, 135.1, 132.2, 130.3, 130.2, 129.8, 128.15, 127.5, 125.7, 97.1, 84.1, 36.7, 36.5, 32.5, 25.3, 24.8; IR (KBr) ν/cm^−1^: 2975 (m), 1791 (m), 1732 (st), 1489 (m), 1443 (m), 1367(st), 1367 (st), 1353 (st); HRMS–EI (*m*/*z*): calcd for C_28_H_34_BN_3_O_5_, 503.2586; found, 503.2576.

## Supporting Information

File 1 ^1^H and ^13^C NMR spectra, 2D spectra where required, and mass spectra for all compounds.
