# Supplementary material for: Aryl nitrile oxide cycloaddition reactions in the presence of pinacol boronic acid ester
Source: Beilstein J Org Chem. 2012 Apr 19;8:606–12. doi: 10.3762/bjoc.8.67 (PMC3343286; doi:10.3762/bjoc.8.67)

Supporting Information

for

Aryl nitrile oxide cycloaddition reactions in the presence of pinacol boronic acid ester

Sarah L. Harding[1], Sebastian M. Marcuccio[2] and G. Paul Savage*[1]

Address: [1]CSIRO Materials Science and Engineering, Private Bag 10, Clayton South MDC, Vic 3169, Australia and [2]Advanced Molecular Technologies Pty Ltd, Unit 1, 7–11 Rocco Drive, Scoresby, VIC 3179, Australia

Email: G. Paul Savage - paul.savage@csiro.au

* Corresponding author

^{1}H and ^{13}C NMR spectra, 2D spectra where required, and mass spectra for all compounds

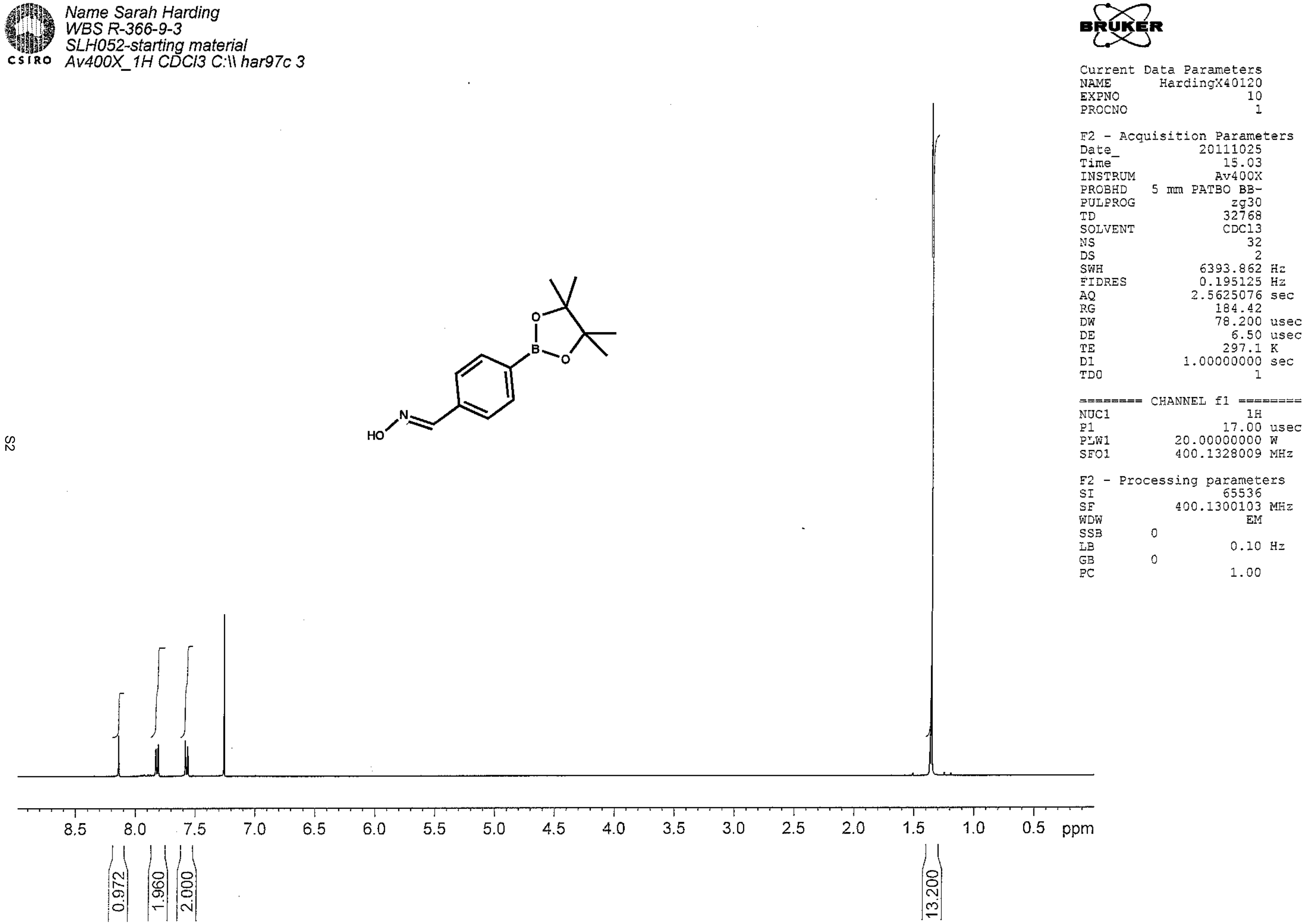
CSIRO
Name Sarah Harding
WBS R-366-9-3
SLH052-starting material
Av400X_1H CDCl3 C:\\ har97c 3
BRUKER
Current Data Parameters
NAME HardingX40120
EXPNO 10
PROCNO 1
F2 - Acquisition Parameters
Date_ 20111025
Time_ 15.03
INSTRUM Av400X
PROBHD 5 mm PATBO BB-
PULPROG zg30
TD 32768
SOLVENT CDCl3
NS 32
DS 2
SWH 6393.862 Hz
FIDRES 0.195125 Hz
AQ 2.5625076 sec
RG 184.42
DW 78.200 usec
DE 6.50 usec
TE 297.1 K
D1 1.00000000 sec
TD0 1
======== CHANNEL f1 ========
NUC1 1H
P1 17.00 usec
PLW1 20.00000000 W
SFO1 400.1328009 MHz
F2 - Processing parameters
SI 65536
SF 400.1300103 MHz
WDW EM
SSB 0
LB 0.10 Hz
GB 0
PC 1.00
HO
N
O
B
O
8.5
8.0
7.5
7.0
6.5
6.0
5.5
5.0
4.5
4.0
3.5
3.0
2.5
2.0
1.5
1.0
0.5
ppm
0.972
1.960
2.000
13.200

Name Sarah Harding
WBS R-00366-09-003
SLH-boronate
Av400X_13C CDCl3 C:\\ har97c 6

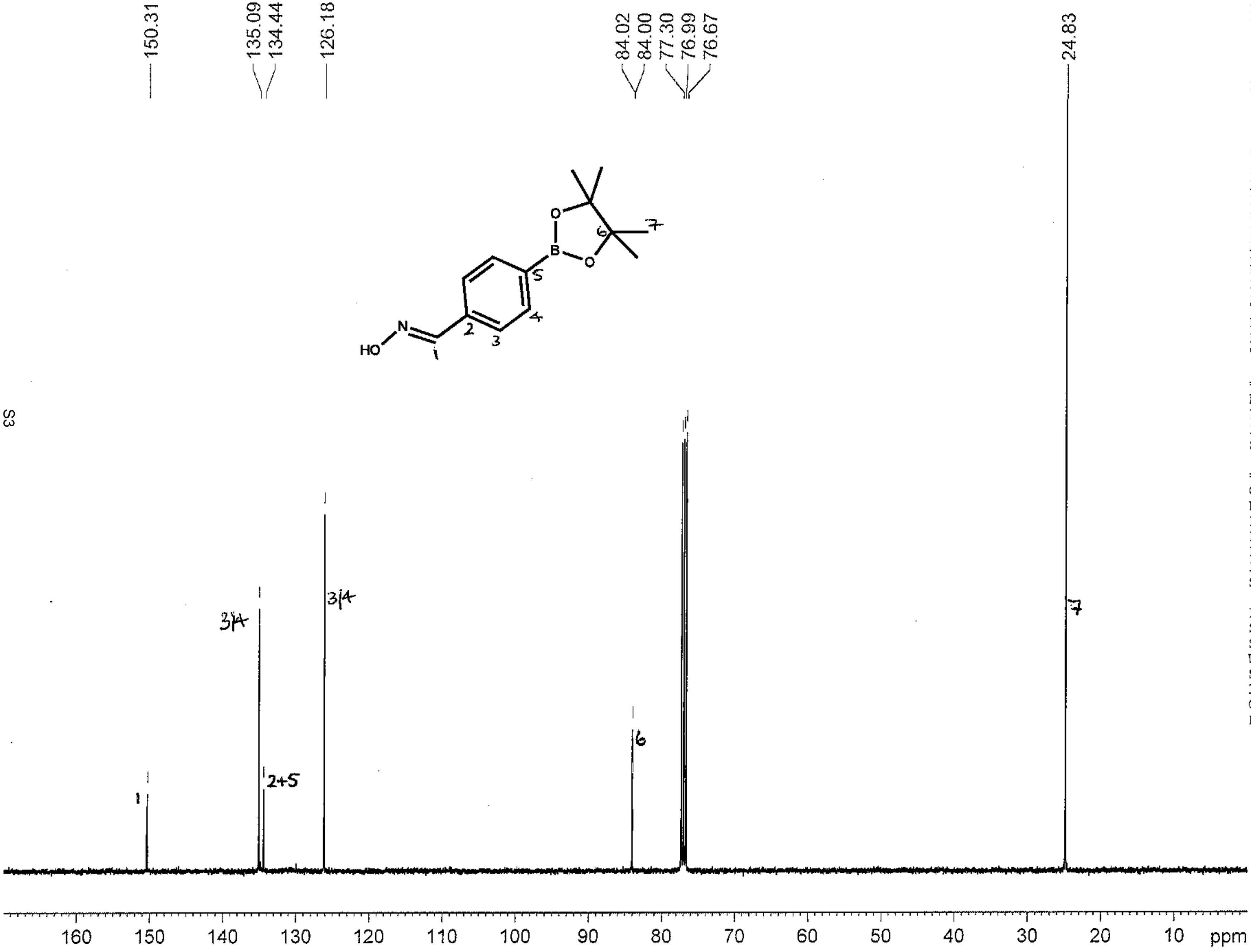


```
Current Data Parameters
NAME       HardingX40198
EXPNO                 11
PROCNO                 1

F2 - Acquisition Parameters
Date_           20111205
Time               12.34
INSTRUM           Av400X
PROBHD    5 mm PATBO BB-
PULPROG          zgpg30
TD                 65536
SOLVENT            CDCl3
NS                   750
DS                     4
SWH           26041.666 Hz
FIDRES         0.397364 Hz
AQ            1.2583412 sec
RG                184.42
DW                19.200 usec
DE                  6.50 usec
TE                 297.1 K
D1            1.00000000 sec
D11           0.03000000 sec
TD0                    1

======== CHANNEL f1 ========
NUC1                 13C
P1                 10.00 usec
PLW1         67.00000000 W
SFO1         100.6248425 MHz

======== CHANNEL f2 ========
CPDPRG2   bi_waltz65_256
NUC2                  1H
PCPD2              90.00 usec
PLW2         20.00000000 W
PLW12         0.71358001 W
PLW13         0.57800001 W
SFO2         400.1316005 MHz

F2 - Processing parameters
SI                 32768
SF           100.6127734 MHz
WDW                   EM
SSB       0
LB                  1.00 Hz
GB        0
PC                  1.40
```

CSIRO

Name Sarah Harding
WBS R-00366-09-003
SLH-boronate
Av400X_hmbc CDCl3 C:\\ har97c 6

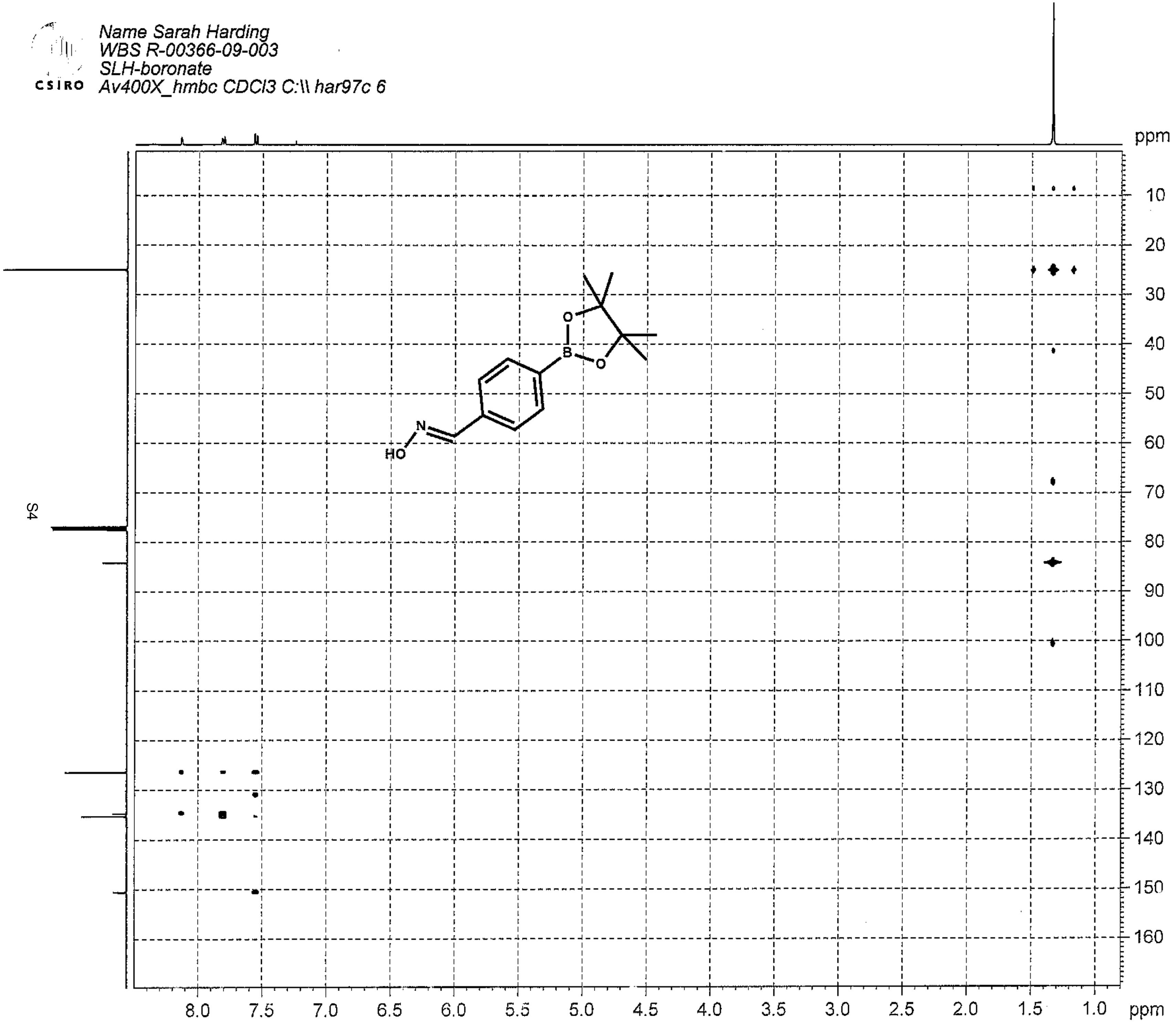

BRUKER

```
Current Data Parameters
NAME         HardingX40198
EXPNO                   14
PROCNO                   1

F2 - Acquisition Parameters
Date_             20111205
Time                 19.04
INSTRUM             Av400X
PROBHD   5 mm PATBO BB-
PULPROG     hmbcetgpl2nd
TD                    4096
SOLVENT              CDCl3
NS                       8
DS                      16
SWH            4795.396 Hz
FIDRES         1.170751 Hz
AQ            0.4271263 sec
RG                  184.42
DW             104.267 usec
DE                6.50 usec
TE                 297.1 K
CNST6        160.0000000
CNST7        110.0000000
CNST13         8.0000000
CNST30         0.5931140
D0          0.00000300 sec
D1          1.50000000 sec
D6          0.06250000 sec
D16         0.00020000 sec
IN0         0.00002260 sec

======== CHANNEL f1 ========
NUC1                    1H
P1               17.00 usec
P2               34.00 usec
PLW1        20.00000000 W
SFO1        400.1322007 MHz

======== CHANNEL f2 ========
NUC2                   13C
P3               10.00 usec
P24            2000.00 usec
PLW2        67.00000000 W
SFO2        100.6233333 MHz
SPNAM7       Crp60comp.4
SPOAL7               0.500
SPOFFS7   0 Hz
SPW7        10.23700047 W

====== GRADIENT CHANNEL =====
GPNAM1        SMSQ10.100
GPNAM3        SMSQ10.100
GPNAM4        SMSQ10.100
GPNAM5        SMSQ10.100
GPZ1              80.00 %
GPZ3              15.00 %
GPZ4             -10.00 %
GPZ5              -5.00 %
P16            1000.00 usec

F1 - Acquisition parameters
TD                     512
SFO1            100.6233 MHz
FIDRES         43.235508 Hz
SW               219.995 ppm
FnMODE     Echo-Antiecho

F2 - Processing parameters
SI                    2048
SF           400.1300178 MHz
WDW                  QSINE
SSB                      2
LB          0 Hz
GB          0
PC                    1.40

F1 - Processing parameters
SI                    2048
MC2        echo-antiecho
SF           100.6127541 MHz
WDW
SSB                      2
LB          0 Hz
GB          0
```

Low Resolution EI Spectrum

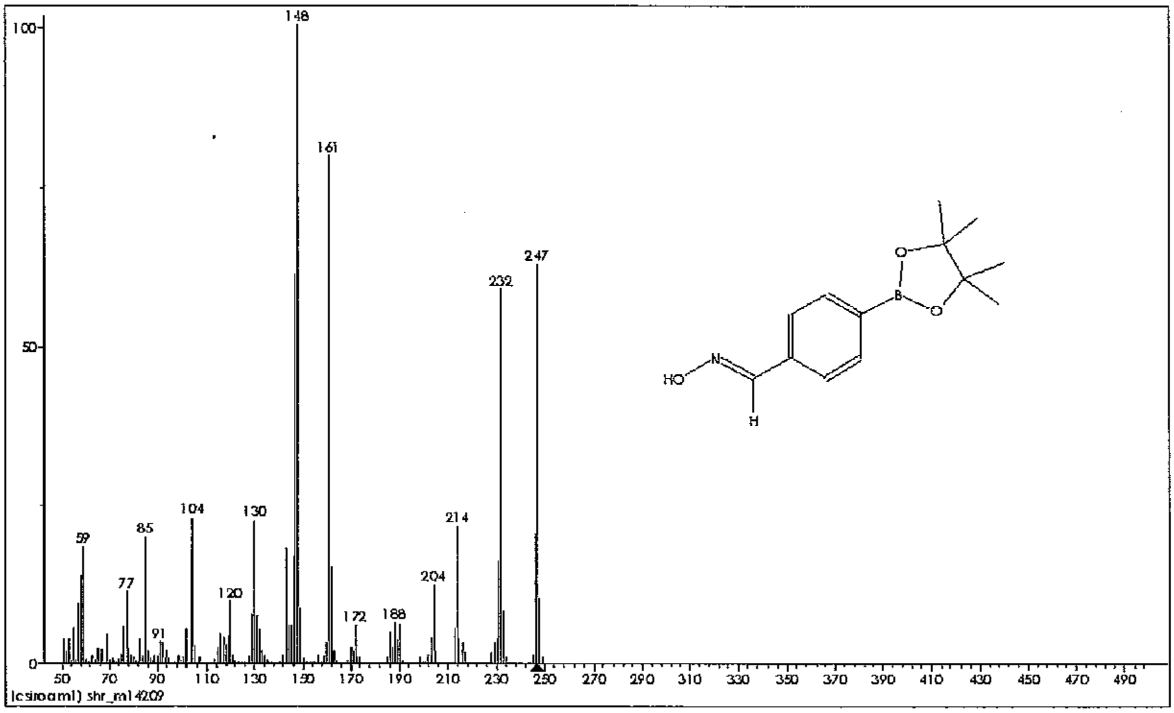

High Resolution EI Spectrum

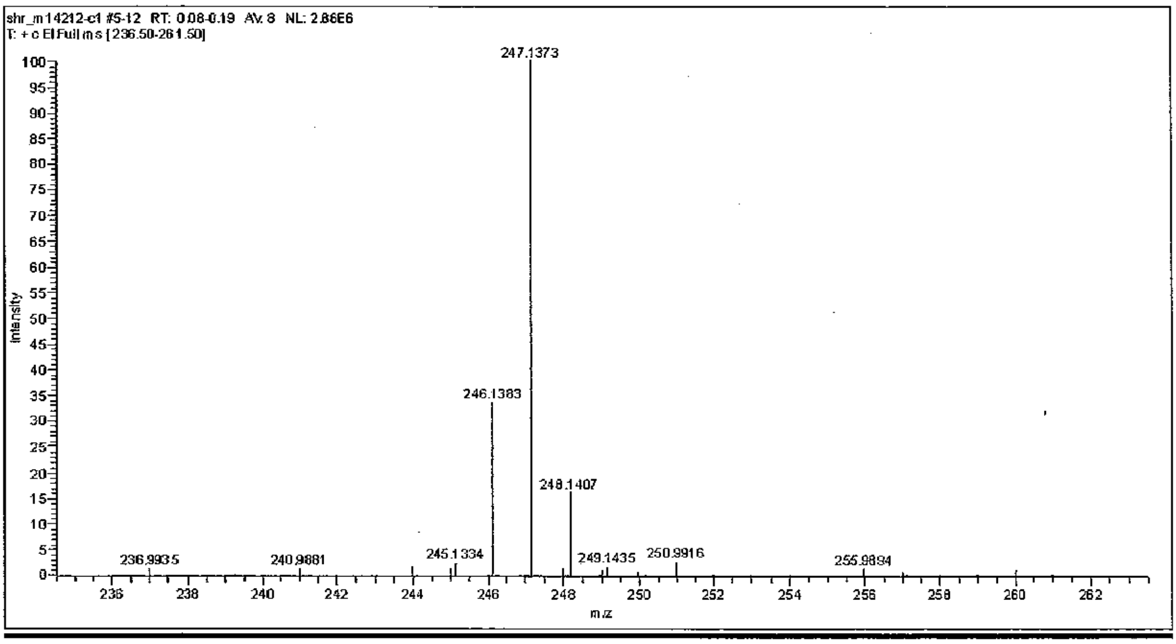

Name Sarah Harding
WBS R-00366-09-003
SLH077af1
Av400X_1H CDCl3 C:\\ har97c 27

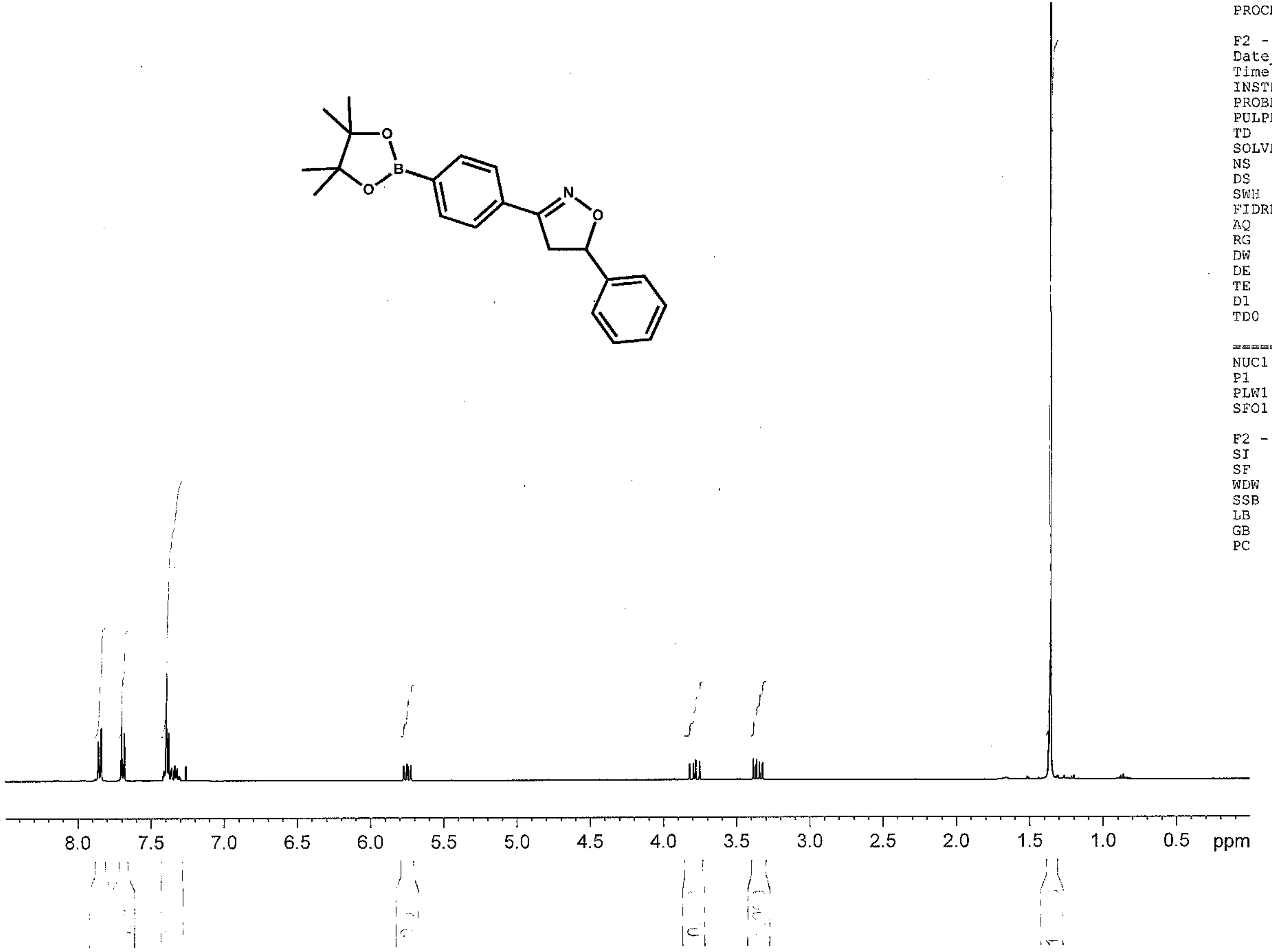

BRUKER

```
Current Data Parameters
NAME       HardingX40194
EXPNO                 10
PROCNO                 1

F2 - Acquisition Parameters
Date_           20111130
Time               16.09
INSTRUM           Av400X
PROBHD   5 mm PATBO BB-
PULPROG             zg30
TD                 32768
SOLVENT            CDCl3
NS                    32
DS                     2
SWH             6393.862 Hz
FIDRES          0.195125 Hz
AQ             2.5625076 sec
RG                 86.92
DW                78.200 usec
DE                  6.50 usec
TE                 297.1 K
D1            1.00000000 sec
TD0                    1

======== CHANNEL f1 ========
NUC1                  1H
P1                 17.00 usec
PLW1         20.00000000 W
SFO1         400.1328009 MHz

F2 - Processing parameters
SI                 65536
SF           400.1300097 MHz
WDW                   EM
SSB      0
LB                  0.10 Hz
GB       0
PC                  1.00
```

Name Sarah Harding
WBS R-00366-09-003
SLH077af1
Av400X_13C CDCl3 C:\\ har97c 27

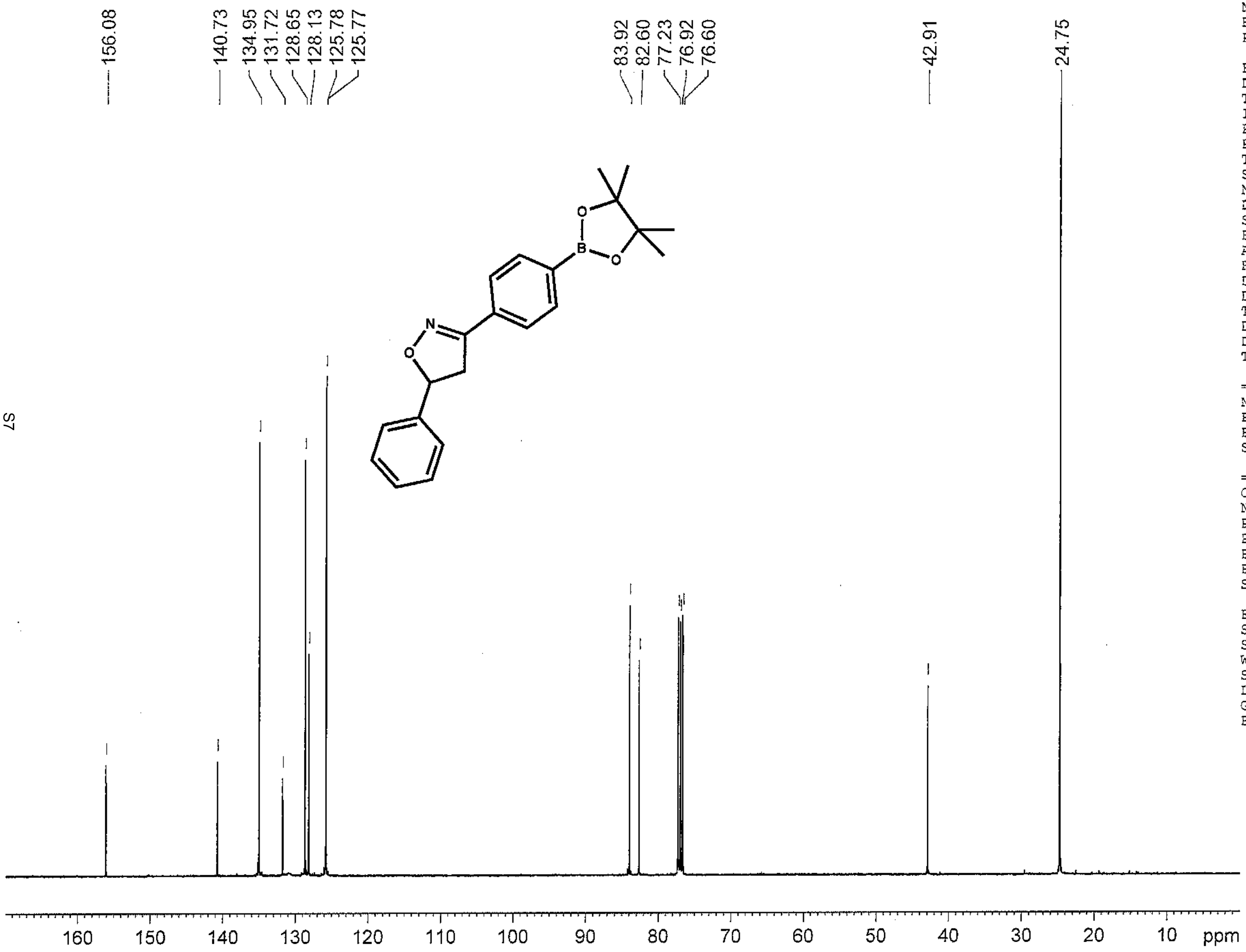

BRUKER

```
Current Data Parameters
NAME          HardingX40194
EXPNO                    15
PROCNO                    1

F2 - Acquisition Parameters
Date_              20111202
Time                   1.14
INSTRUM              Av400X
PROBHD       5 mm PATBO BB-
PULPROG             zgpg30
TD                    65536
SOLVENT               CDCl3
NS                     6000
DS                        4
SWH              26041.666 Hz
FIDRES            0.397364 Hz
AQ               1.2583412 sec
RG                   184.42
DW                   19.200 usec
DE                     6.50 usec
TE                    297.1 K
D1               1.00000000 sec
D11              0.03000000 sec
TD0                       1

======== CHANNEL f1 ========
NUC1                    13C
P1                    10.00 usec
PLW1            67.00000000 W
SFO1            100.6248425 MHz

======== CHANNEL f2 ========
CPDPRG2     bi_waltz65_256
NUC2                     1H
PCPD2                 90.00 usec
PLW2            20.00000000 W
PLW12            0.71358001 W
PLW13            0.57800001 W
SFO2            400.1316005 MHz

F2 - Processing parameters
SI                    32768
SF              100.6127829 MHz
WDW                      EM
SSB      0
LB                     1.00 Hz
GB       0
PC                     1.40
```

CSIRO Materials Science & Engineering
S.Harding

Accurate Mass Report
SLH077af1 (a)

CSIRO

Low Resolution EI Spectrum

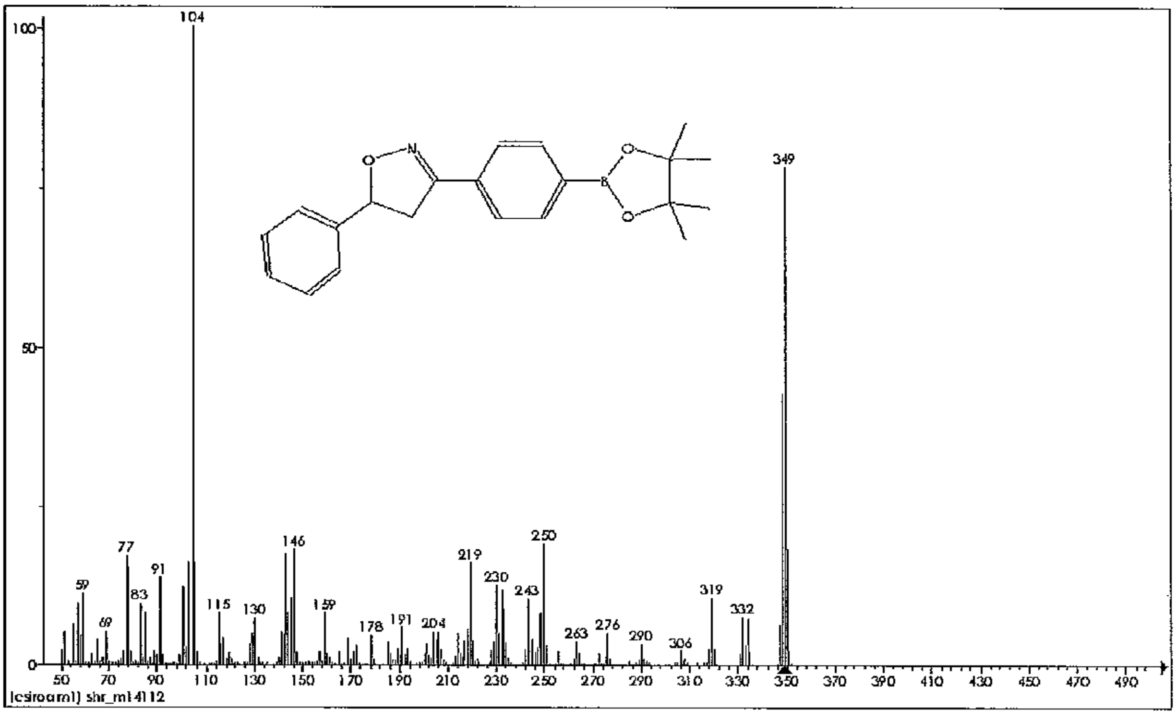

High Resolution EI Spectrum

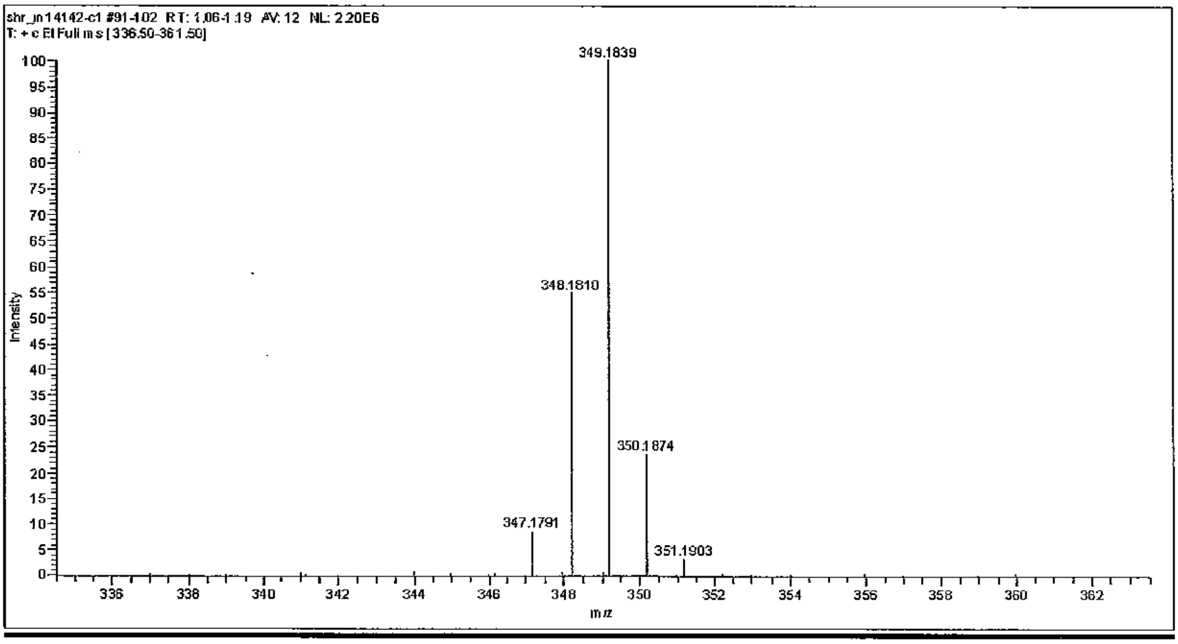

Australian Science, Australia's Future

Name Sarah Harding
WBS R-00366-09-003
SLH077cf1 C
Av400X_1H CDCl3 C:\\ har97c 26

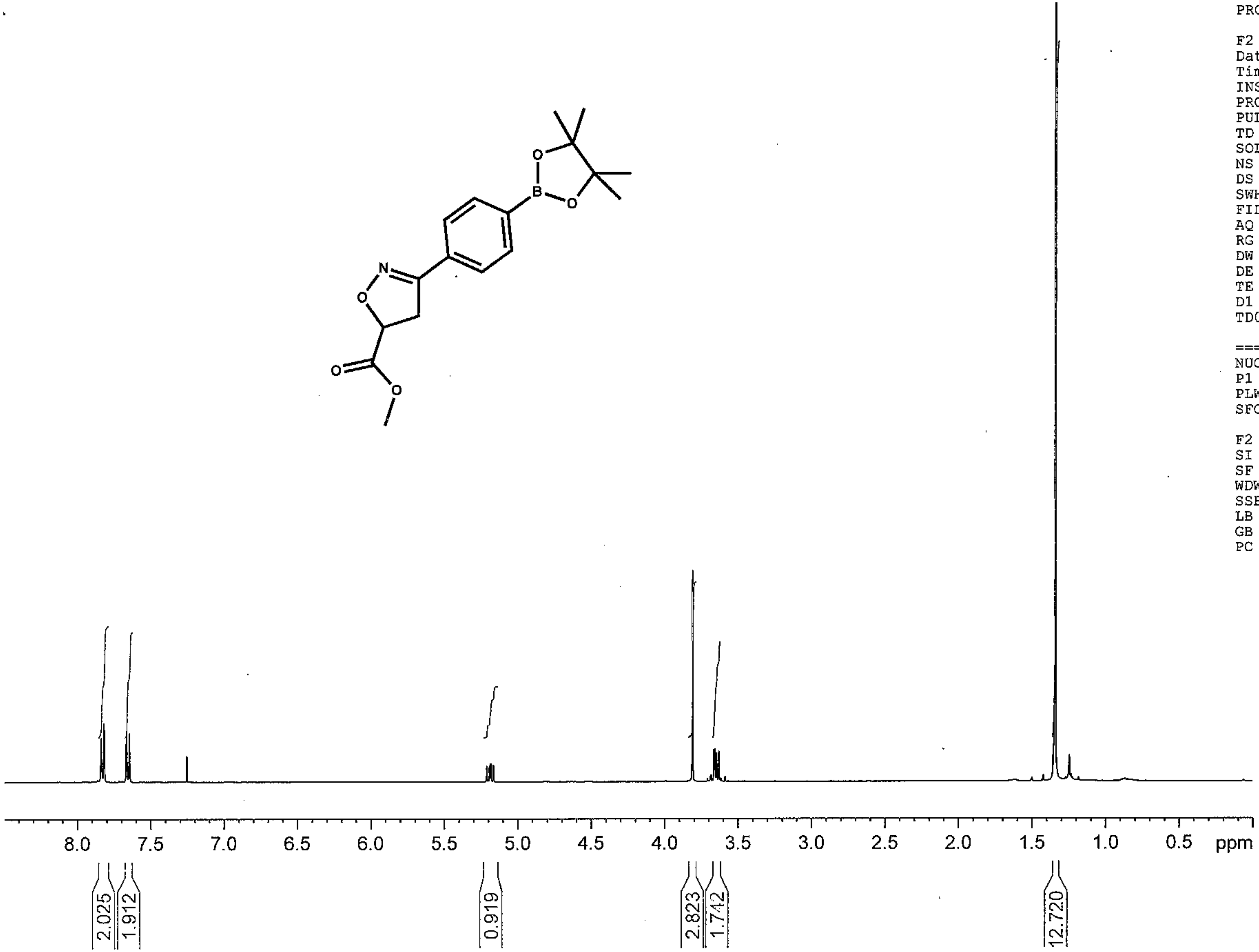

BRUKER

```
Current Data Parameters
NAME        HardingX40196
EXPNO                  10
PROCNO                  1

F2 - Acquisition Parameters
Date_            20111201
Time                14.50
INSTRUM            Av400X
PROBHD    5 mm PATBO BB-
PULPROG              zg30
TD                  32768
SOLVENT             CDCl3
NS                     32
DS                      2
SWH              6393.862 Hz
FIDRES           0.195125 Hz
AQ              2.5625076 sec
RG                 119.37
DW                 78.200 usec
DE                   6.50 usec
TE                  297.1 K
D1             1.00000000 sec
TD0                     1

======== CHANNEL f1 ========
NUC1                   1H
P1                  17.00 usec
PLW1          20.00000000 W
SFO1          400.1328009 MHz

F2 - Processing parameters
SI                  65536
SF            400.1300127 MHz
WDW                    EM
SSB      0
LB                   0.10 Hz
GB       0
PC                   1.00
```

Name Sarah Harding
WBS R-00366-09-003
SLH077cf1
Av400X_13C CDCl3 C:\\ har97c 26

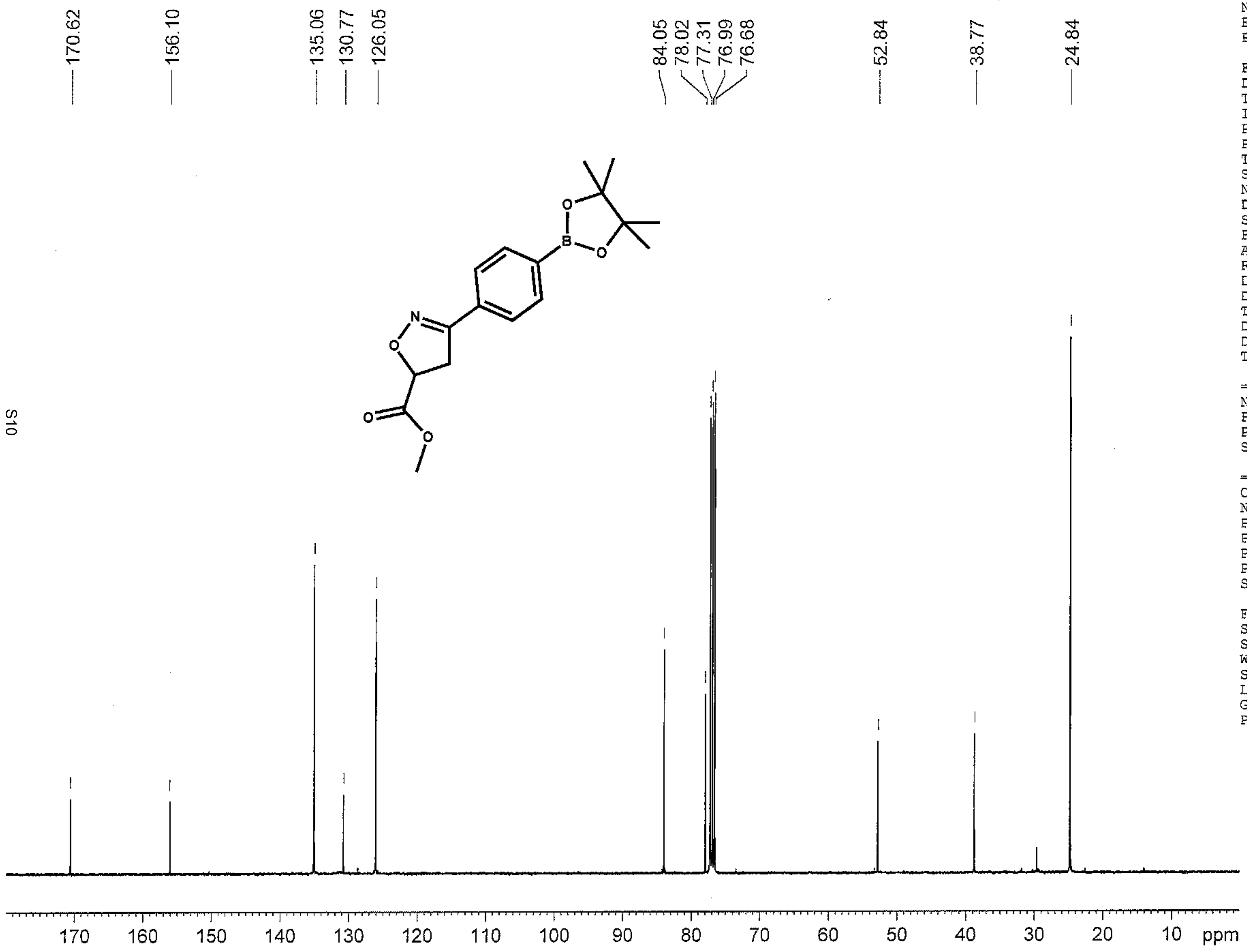


```
Current Data Parameters
NAME       HardingX40196
EXPNO                 11
PROCNO                 1

F2 - Acquisition Parameters
Date_           20111202
Time                6.52
INSTRUM           Av400X
PROBHD    5 mm PATBO BB-
PULPROG          zgpg30
TD                 65536
SOLVENT            CDCl3
NS                  5000
DS                     4
SWH            26041.666 Hz
FIDRES          0.397364 Hz
AQ             1.2583412 sec
RG                184.42
DW                19.200 usec
DE                  6.50 usec
TE                 297.1 K
D1            1.00000000 sec
D11           0.03000000 sec
TD0                    1

======== CHANNEL f1 ========
NUC1                 13C
P1                 10.00 usec
PLW1         67.00000000 W
SFO1        100.6248425 MHz

======== CHANNEL f2 ========
CPDPRG2   bi_waltz65_256
NUC2                  1H
PCPD2              90.00 usec
PLW2         20.00000000 W
PLW12         0.71358001 W
PLW13         0.57800001 W
SFO2        400.1316005 MHz

F2 - Processing parameters
SI                 32768
SF           100.6127734 MHz
WDW                   EM
SSB      0
LB                  1.00 Hz
GB       0
PC                  1.40
```

CSIRO Materials Science & Engineering
S.Harding

Accurate Mass Report
SLH077cf1 (c)

CSIRO

Low Resolution EI Spectrum

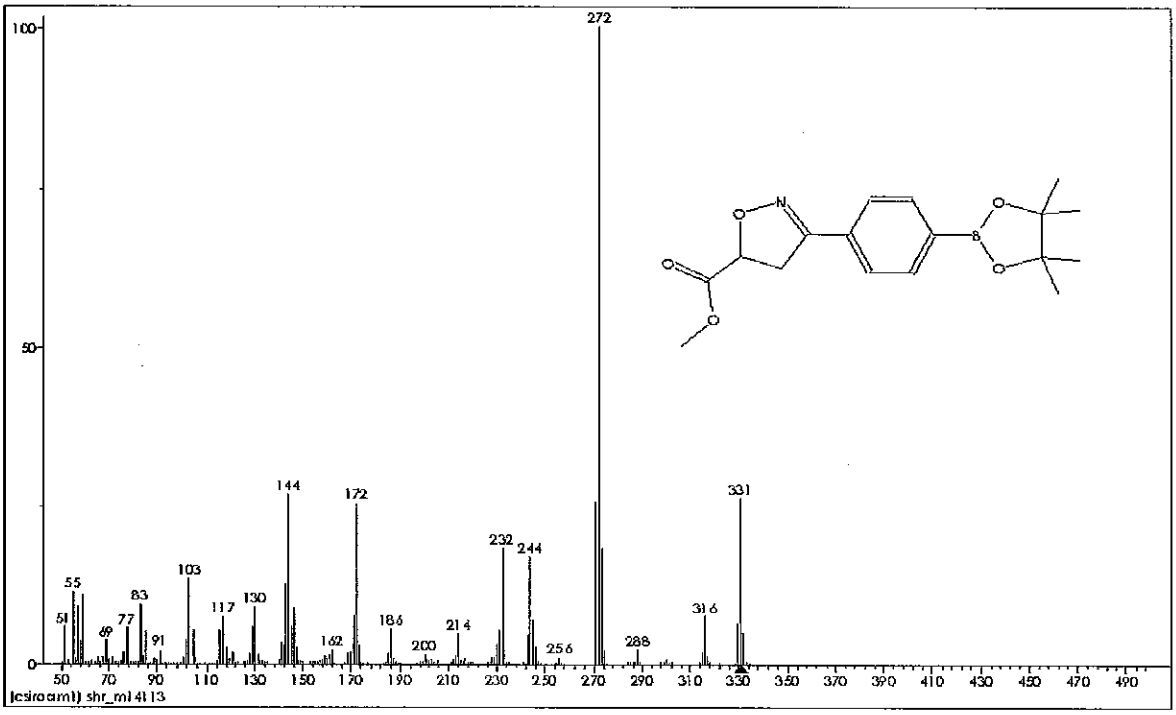

High Resolution EI Spectrum

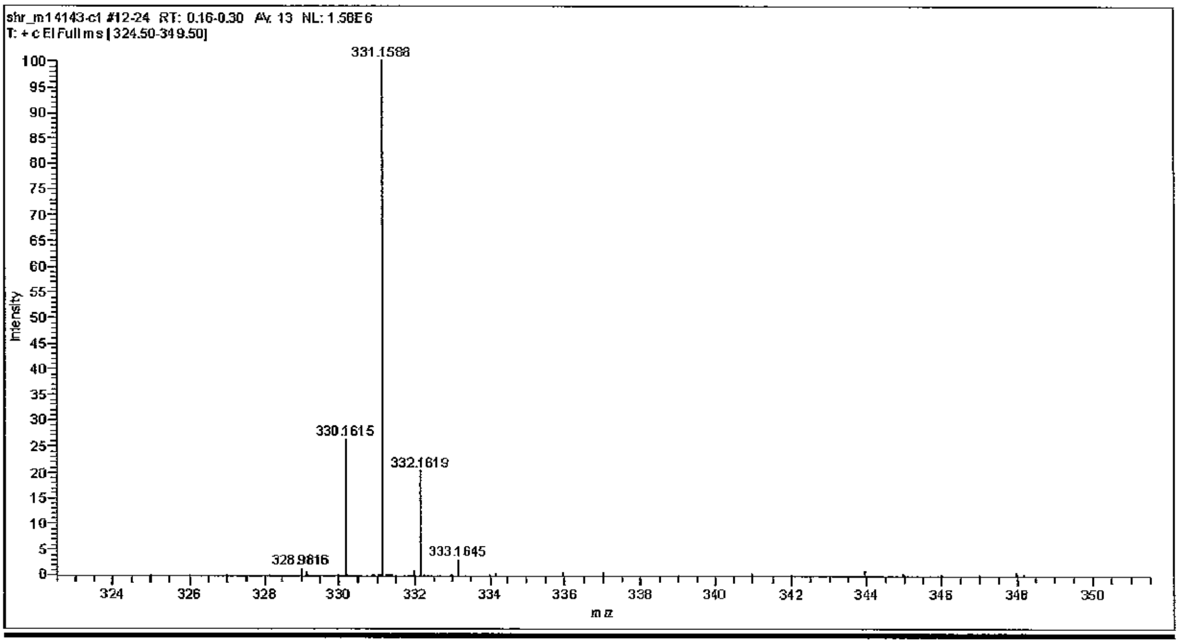

CSIRO Name Sarah Harding
WBS R-00366-09-003
SLH078ef1
Av400X_1H CDCl3 C:\\ har97c 26

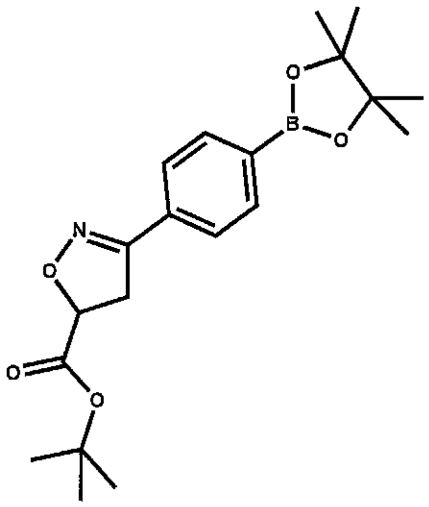

BRUKER

```
Current Data Parameters
NAME        HardingX40206
EXPNO                  10
PROCNO                  1

F2 - Acquisition Parameters
Date_            20111206
Time                16.34
INSTRUM            Av400X
PROBHD   5 mm PATBO BB-
PULPROG              zg30
TD                  32768
SOLVENT             CDCl3
NS                     32
DS                      2
SWH              6393.862 Hz
FIDRES           0.195125 Hz
AQ              2.5625076 sec
RG                   75.1
DW                 78.200 usec
DE                   6.50 usec
TE                  297.1 K
D1             1.00000000 sec
TD0                     1

======== CHANNEL f1 ========
NUC1                   1H
P1                  17.00 usec
PLW1          20.00000000 W
SFO1          400.1328009 MHz

F2 - Processing parameters
SI                  65536
SF            400.1300178 MHz
WDW                    EM
SSB      0
LB                   0.10 Hz
GB       0
PC                   1.00
```

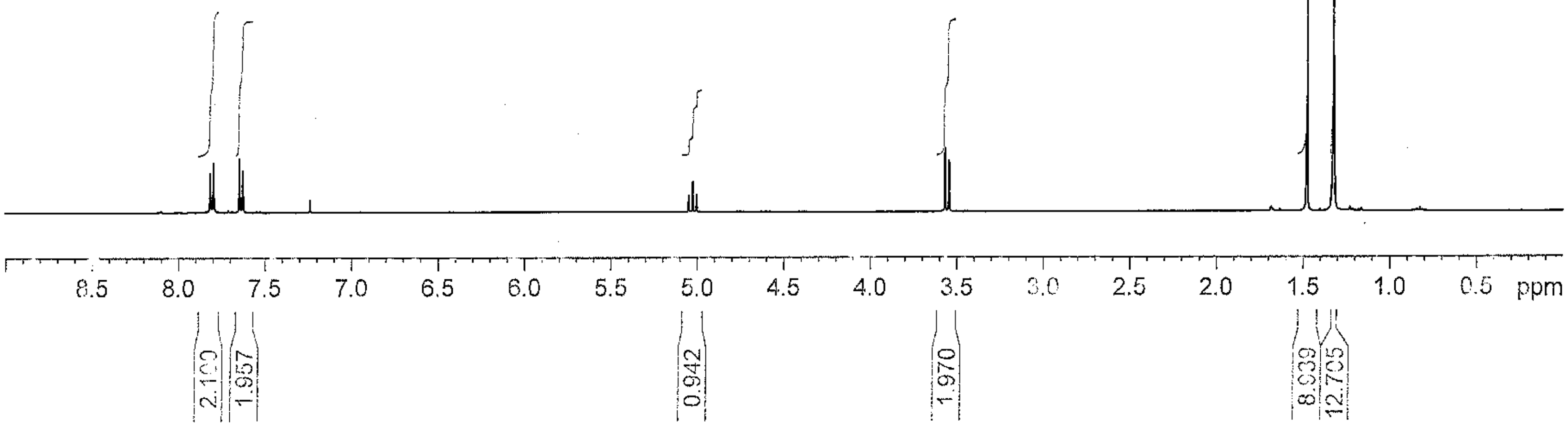

Name Sarah Harding
WBS R-00366-09-003
SLH078ef1
CSIRO Av400X_13C CDCl3 C:\\ har97c 26

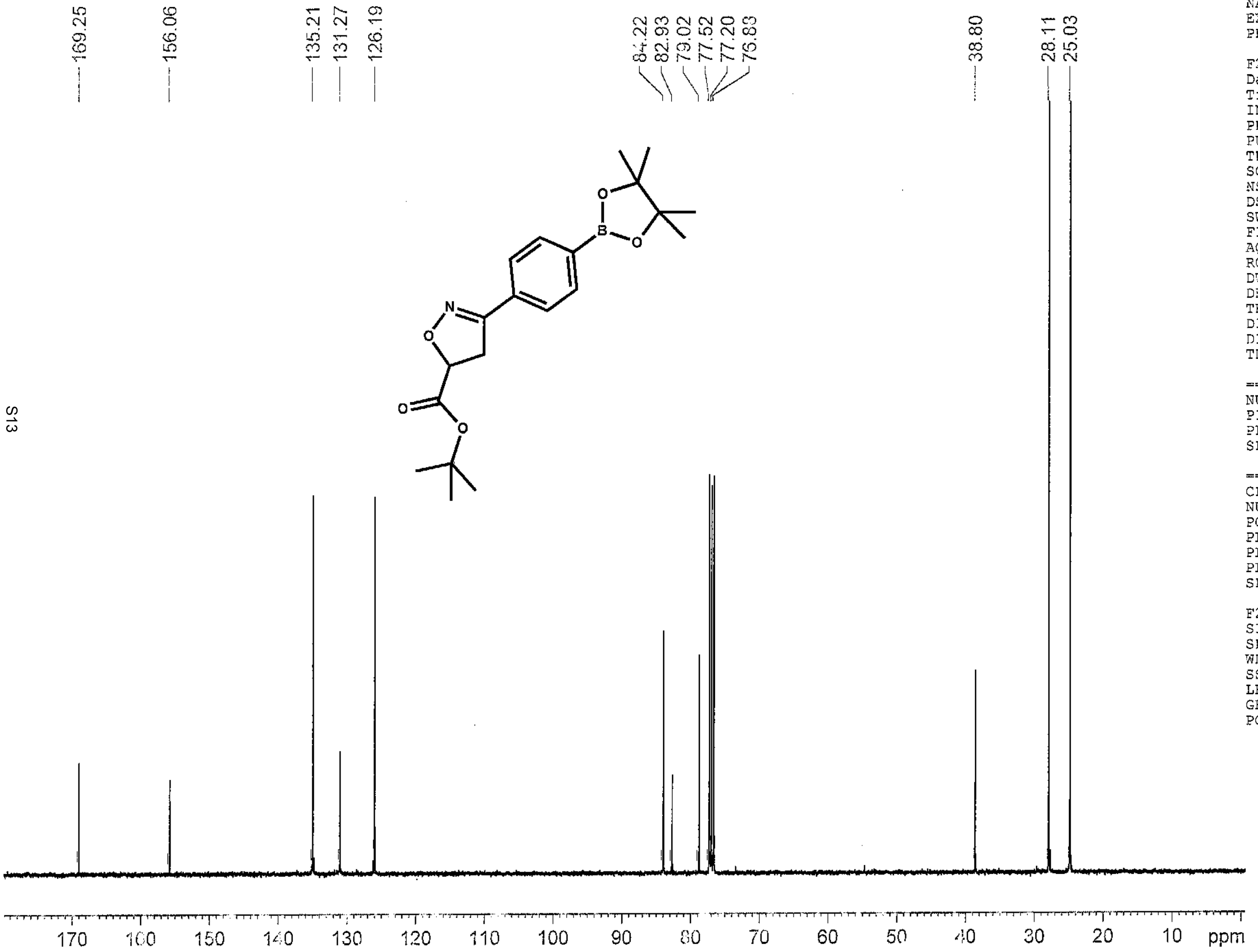


```
Current Data Parameters
NAME       HardingX40206
EXPNO                 11
PROCNO                 1

F2 - Acquisition Parameters
Date_           20111206
Time               17.18
INSTRUM           Av400X
PROBHD   5 mm PAT30 BF-
PULPROG          zgpg30
TD                 65536
SOLVENT            CDCl3
NS                   750
DS                     4
SWH            26041.666 Hz
FIDRES          0.397364 Hz
AQ             1.2583412 sec
RG                184.42
DW                19.200 usec
DE                  6.50 usec
TE                 297.1 K
D1            1.00000000 sec
D11           0.03000000 sec
TD0                    1

======== CHANNEL f1 ========
NUC1                 13C
P1                 10.00 usec
PLW1         67.00000000 W
SFO1         100.6248425 MHz

======== CHANNEL f2 ========
CPDPRG2  bi_waltz65_256
NUC2                  1H
PCPD2              90.00 usec
PLW2         20.00000000 W
PLW12         0.71358001 W
PLW13         0.57800001 W
SFO2         400.1316005 MHz

F2 - Processing parameters
SI                 32768
SF           100.6127768 MHz
WDW                   EM
SSB      0
LB                  1.00 Hz
GB       0
PC                  1.40
```

Name Sarah Harding
WBS R-00366-09-003
SLH078ef1
CSIRO Av400X_hmbc CDCl3 C:\\ har97c 26

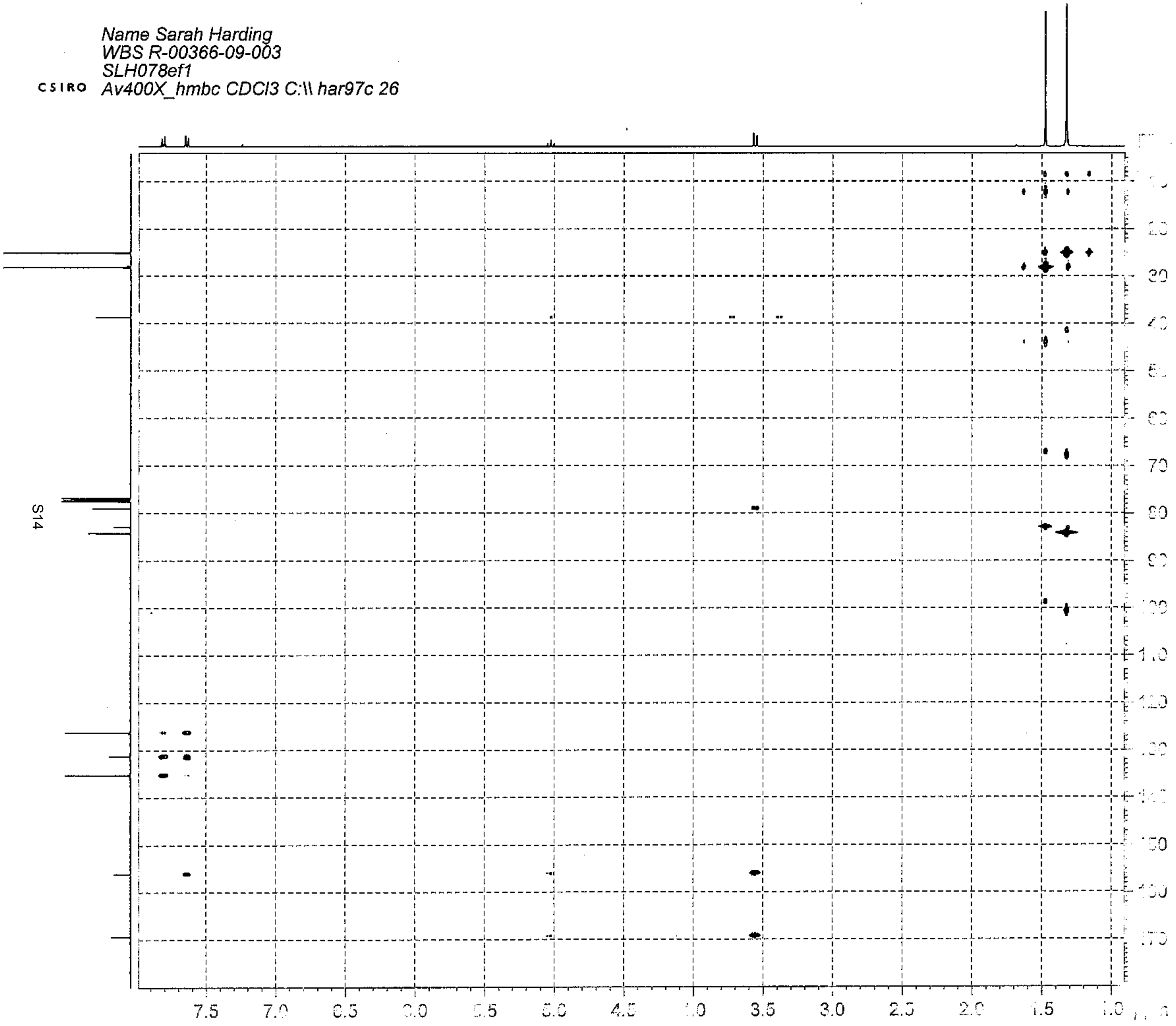

S14

BRUKER

Current Data Parameters
NAME HardingX40206
EXPNO 14
PROCNO 1

F2 - Acquisition Parameters
Date_ 20111207
Time 9.42
INSTRUM Av400X
PROBHD 5 mm PATBO BB-
PULPROG hmbcetgpl2nd
TD 4096
SOLVENT CDCl3
NS 8
DS 16
SWH 4795.396 Hz
FIDRES 1.170751 Hz
AQ 0.4271263 sec
RG 181.42
DW 104.[illegible] usec
DE 6.50 usec
TE 297.0 K
CNST6 120.0000000
CNST7 110.0000000
CNST13 3.0000000
CNST30 0.5981140
D0 0.00000300 sec
D1 1.50000000 sec
D6 0.03625000 sec
D16 0.00020000 sec
IN0 0.00002260 sec

======== CHANNEL f1 ========
NUC1 1H
P1 17.00 usec
P2 34.00 usec
PLW1 20.00000000 W
SFO1 400.1322007 MHz

======== CHANNEL f2 ========
NUC2 13C
P3 10.00 usec
P24 2000.00 usec
PLW2 67.00000000 W
SFO2 100.6233333 MHz
SPNAM7 Crp60comp.4
SPOAL7 0.500
SPOFFS7 0 Hz
SPW7 10.23700047 W

======== GRADIENT CHANNEL ========
GPNAM1 SMSQ10.100
GPNAM3 SMSQ10.100
GPNAM4 SMSQ10.100
GPNAM5 SMSQ10.100
GPZ1 80.00 %
GPZ3 15.00 %
GPZ4 10.00 %
GPZ5 -5.00 %
P16 1000.00 usec

F1 - Acquisition parameters
TD 512
SFO1 100.6233 MHz
FIDRES 43.235508 Hz
SW 219.995 ppm
FnMODE Echo-Antiecho

F2 - Processing parameters
SI 2048
SF 400.1300178 MHz
WDW QSINE
SSB 2
LB 0 Hz
GB 0
PC 1.40

F1 - Processing parameters
SI 2048
MC2 echo-antiecho
SF 100.6127541 MHz
WDW [illegible]
SSB 0
LB 0 Hz
GB 0

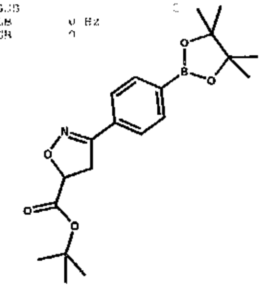

CSIRO Materials Science & Engineering — Accurate Mass Report

S.Harding — SLH078ef1 E

CSIRO

Low Resolution EI Spectrum

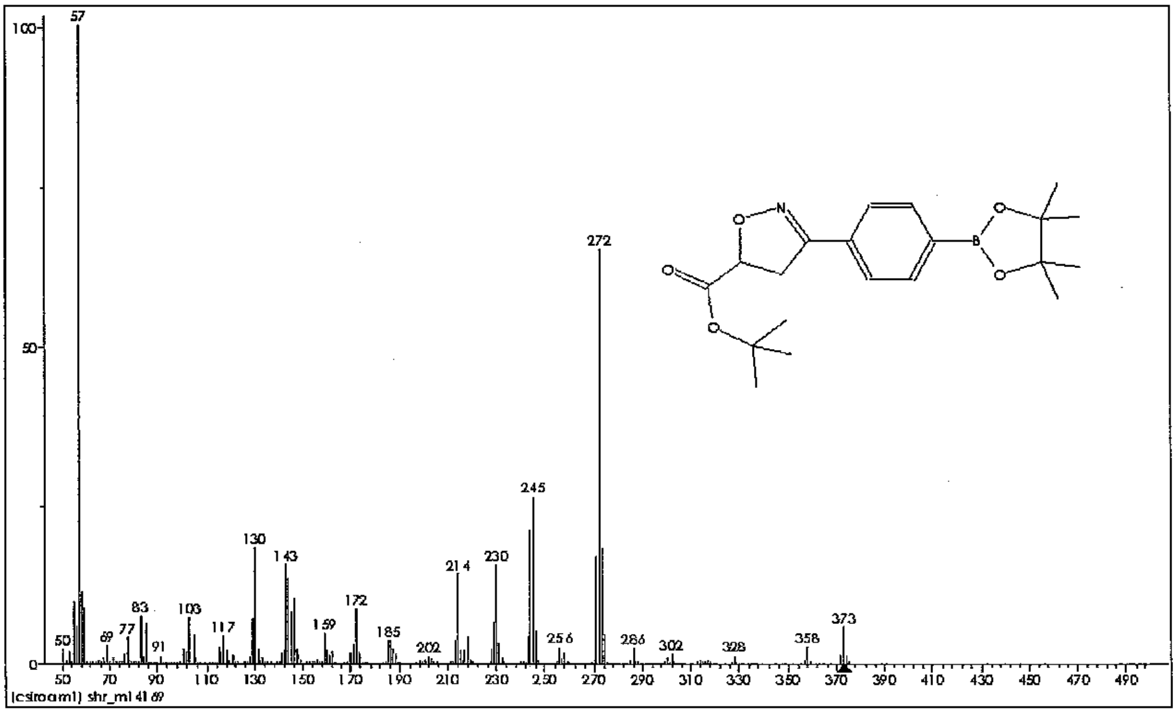

High Resolution EI Spectrum

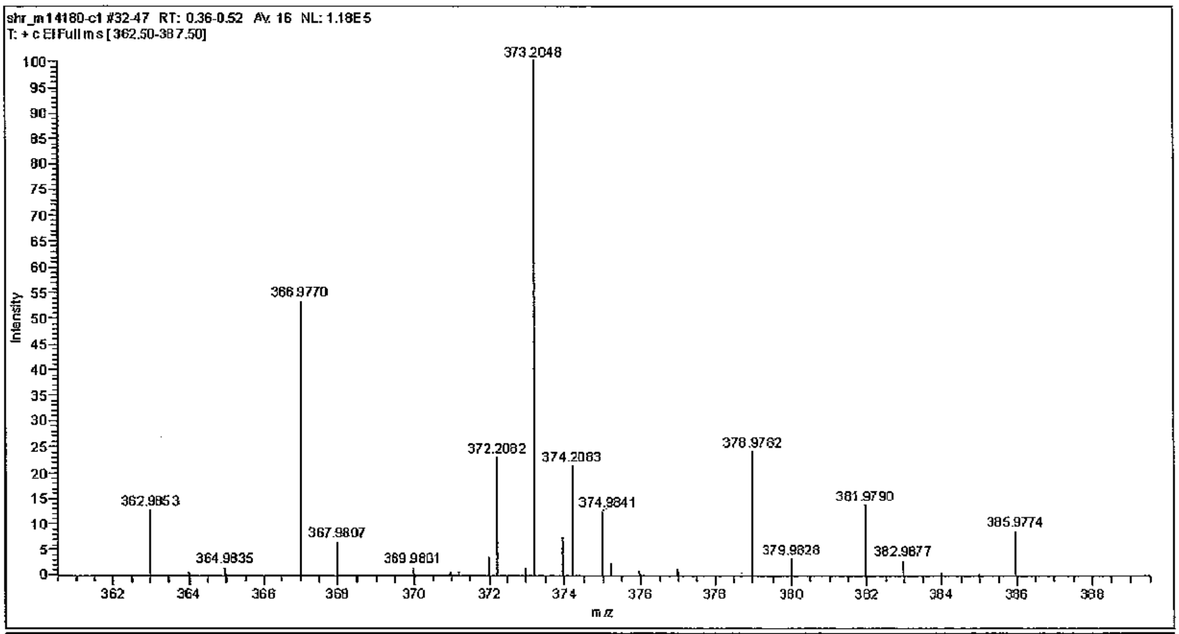

Australian Science, Australia's Future

Name Sarah Harding
WBS R-00366-09-003
SLH077bf1
CSIRO Av400X_1H CDCl3 C:\\ har97c 31

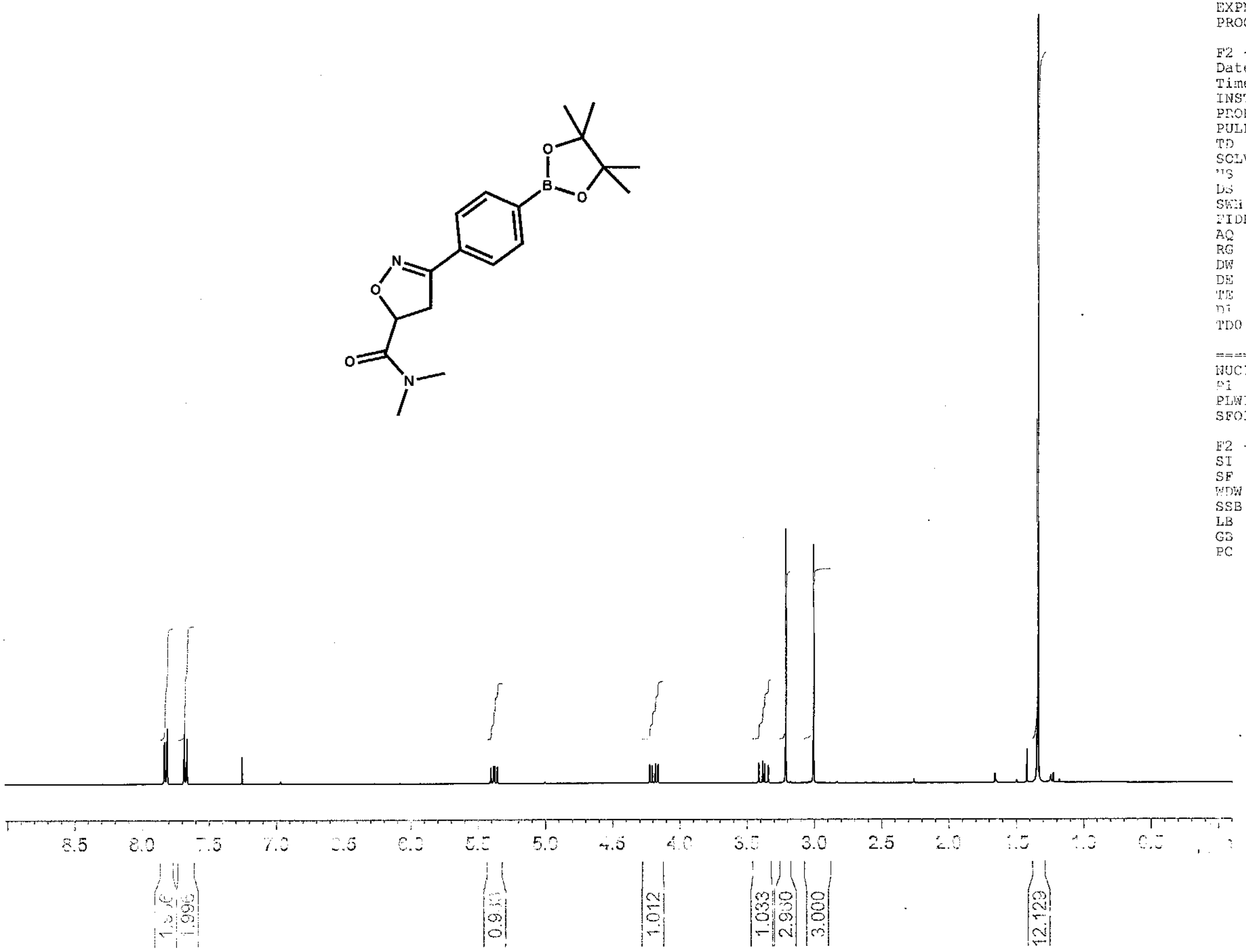

BRUKER

Curr[illegible]t Data Parameters
NAME HardingX40208
EXPNO 10
PROCNO 1

F2 - Acquisition Parameters
Date_ 20111206
Time 18.15
INSTRUM Av400X
PROBHD 5 mm PATBO BB-
PULPROG zg30
TD 32768
SOLVENT CDCl3
NS 32
DS 2
SWH 6393.862 Hz
FIDRES 0.195125 Hz
AQ 2.5625076 sec
RG 132.48
DW 78.200 usec
DE 6.50 usec
TE 297.0 K
D1 1.00000000 sec
TD0 1

======== CHANNEL f1 ========
NUC1 1H
P1 17.00 usec
PLW1 20.00000000 W
SFO1 400.1328009 MHz

F2 - Processing parameters
SI 65536
SF 400.1300127 MHz
WDW EM
SSB 0
LB 0.10 Hz
GB 0
PC 1.00

CSIRO Name Sarah Harding
WBS R-00366-09-003
SLH077bf1
Av400X_13C CDCl3 C:\\ har97c 31

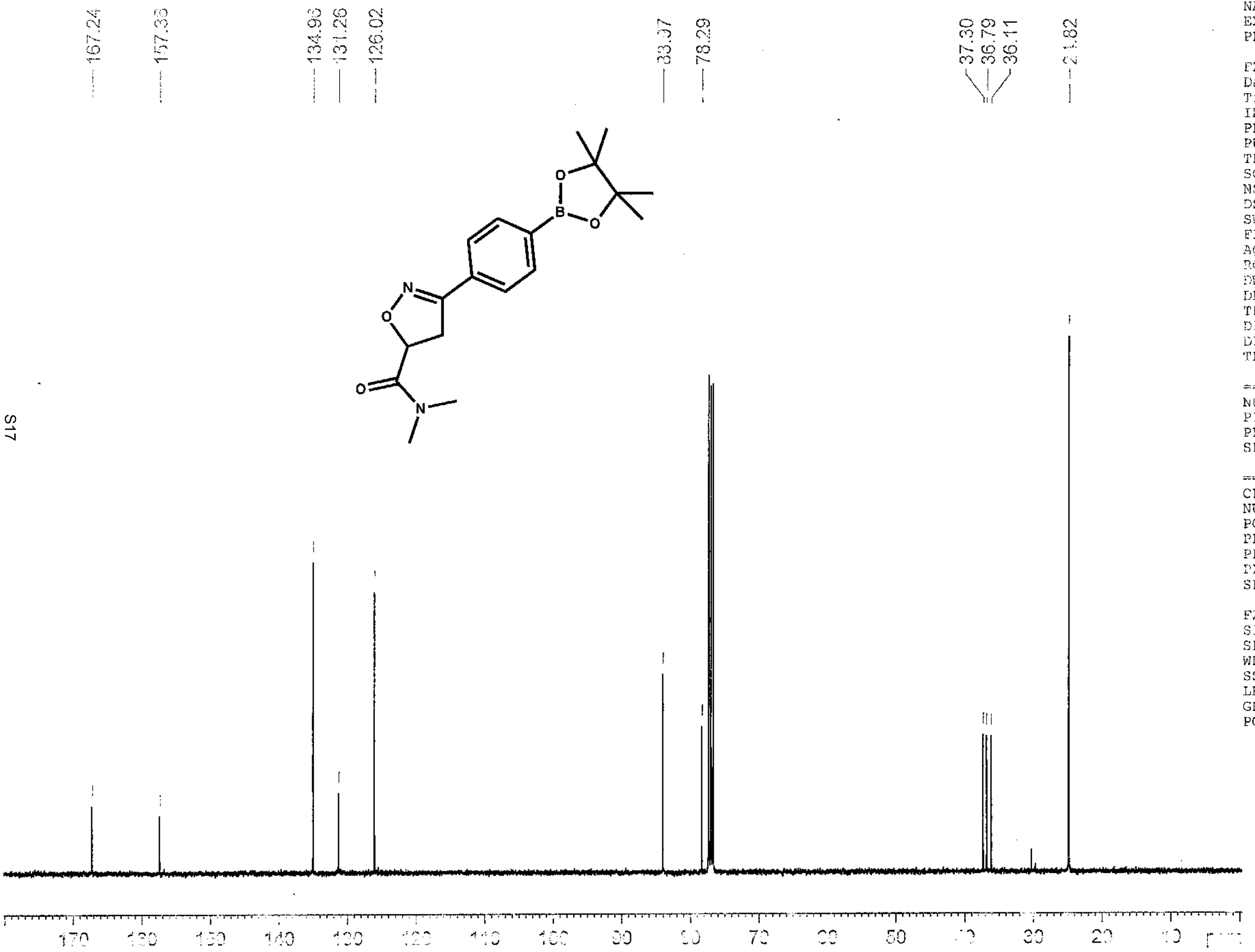

BRUKER

```
Current Data Parameters
NAME        HardingX40208
EXPNO                  11
PROCNO                  1

F2 - Acquisition Parameters
Date_            20111207
Time                 3.42
INSTRUM            Av400X
PROBHD    5 mm PATBO BB-
PULPROG            zgpg30
TD                  65536
SOLVENT             CDCl3
NS                   1000
DS                      4
SWH             26041.666 Hz
FIDRES           0.397364 Hz
AQ              1.2583412 sec
RG                 184.42
DW                 19.200 usec
DE                   6.50 usec
TE                  297.1 K
D1             1.00000000 sec
D11            0.03000000 sec
TD0                     1

======== CHANNEL f1 ========
NUC1                  13C
P1                  10.00 usec
PLW1          67.00000000 W
SFO1          100.6248425 MHz

======== CHANNEL f2 ========
CPDPRG2   bi_waltz65_256
NUC2                   1H
PCPD2               90.00 usec
PLW2          20.00000000 W
PLW12          0.71358001 W
PLW13          0.57800001 W
SFO2          400.1316005 MHz

F2 - Processing parameters
SI                  32768
SF            100.6127758 MHz
WDW                    EM
SSB       0
LB                   1.00 Hz
GB        0
PC                   1.40
```

Name Sarah Harding
WBS R-00366-09-003
SLH077bf1
CSIRO Av400X_cosy CDCl3 C:\\ har97c 31

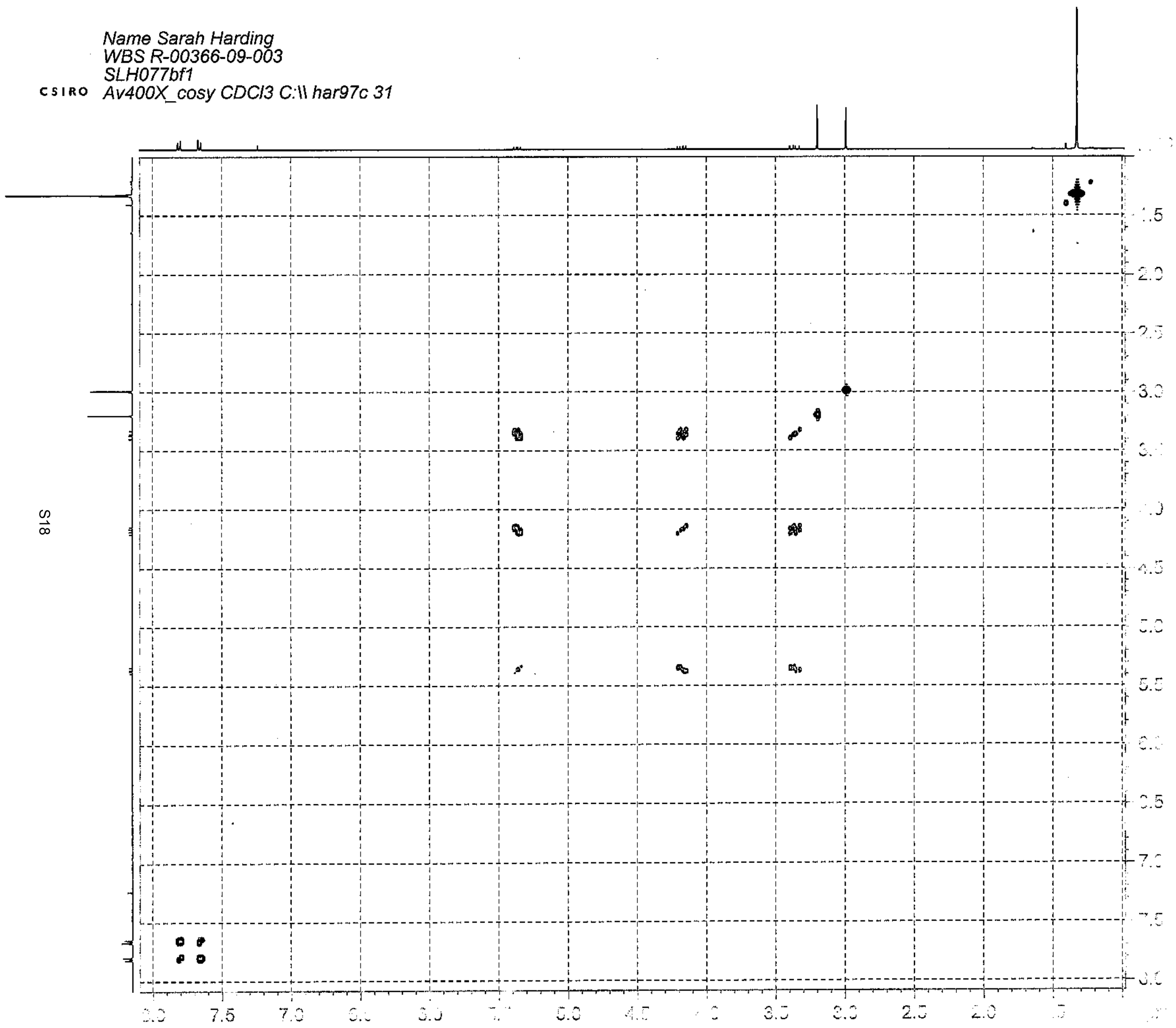

BRUKER

Current Data Parameters
NAME Harding240208
EXPNO 12
PROCNO 1

F2 - Acquisition Parameters
Date_ 20111207
Time 3.43
INSTRUM Av400X
PROBHD 5 mm PATBO BB-
PULPROG cosygpqf45_CSIRO
TD 2048
SOLVENT CDCl3
NS 1
DS 16
SWH 4807.692 Hz
FIDRES 2.347506 Hz
AQ 0.2130420 sec
RG 184.42
DW 104.000 usec
DE 6.50 usec
TE 296.0 K
D0 0.00000300 sec
D1 1.00000000 sec
D13 0.00000400 sec
D16 0.00020000 sec
IN0 0.00020800 sec

======== CHANNEL f1 ========
NUC1 1H
P0 8.50 usec
P1 17.00 usec
PLW1 20.00000000 W
SFO1 400.1322007 MHz

======== GRADIENT CHANNEL =====
GPNAM1 sms010.100
GPZ1 10.00 %
P16 1000.00 usec

F1 - Acquisition parameters
TD 512
SFO1 400.1322 MHz
FIDRES 9.390023 Hz
SW 12.015 ppm
FnMODE QF

F2 - Processing parameters
SI 1024
SF 400.1300178 MHz
WDW SINE
SSB 0
LB 0 Hz
GB 0
PC 1.00

F1 - Processing parameters
SI 1024
MC2 QF
SF 400.1300178 MHz
WDW
SSB 0
LB 0 Hz
GB 0

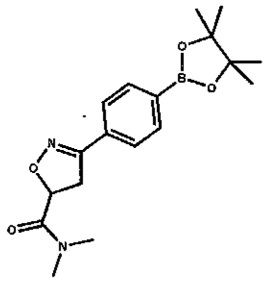

13/12/2011

CSIRO

CSIRO Materials Science & Engineering **Accurate Mass Report**
S.Harding **SLH077bf1** ß.

Low Resolution EI Spectrum

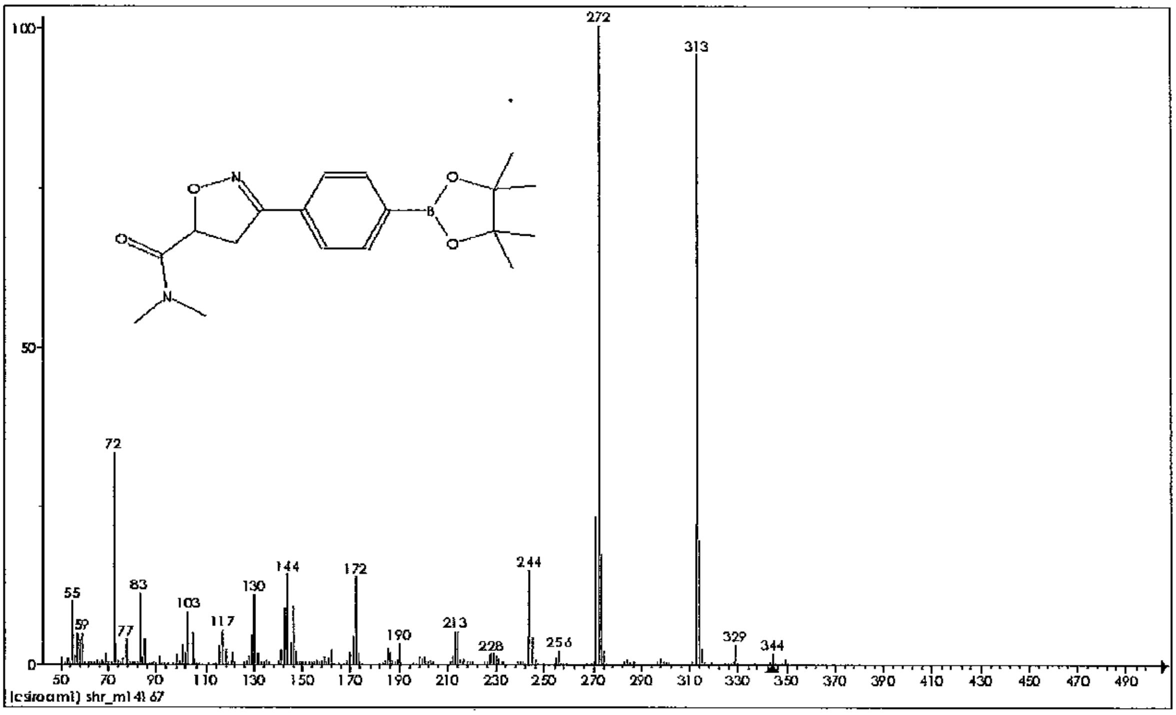

High Resolution EI Spectrum

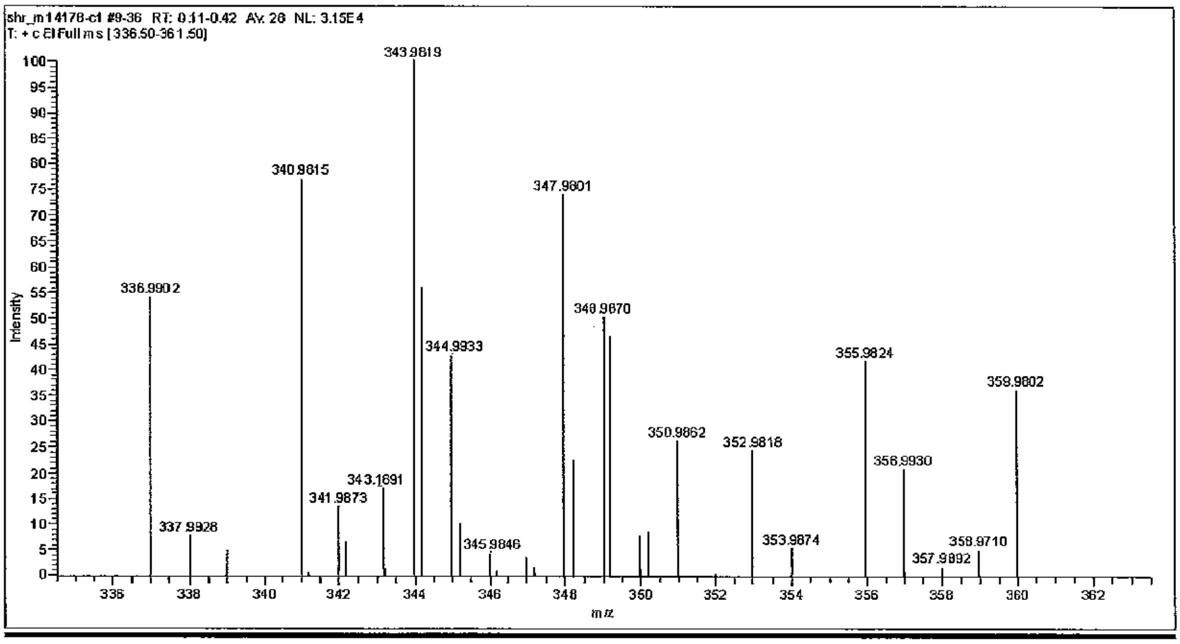

Name Sarah Harding
WBS R-00366-09-003
SLH078df1
CSIRO Av400X_1H CDCl3 C:\\ har97c 32

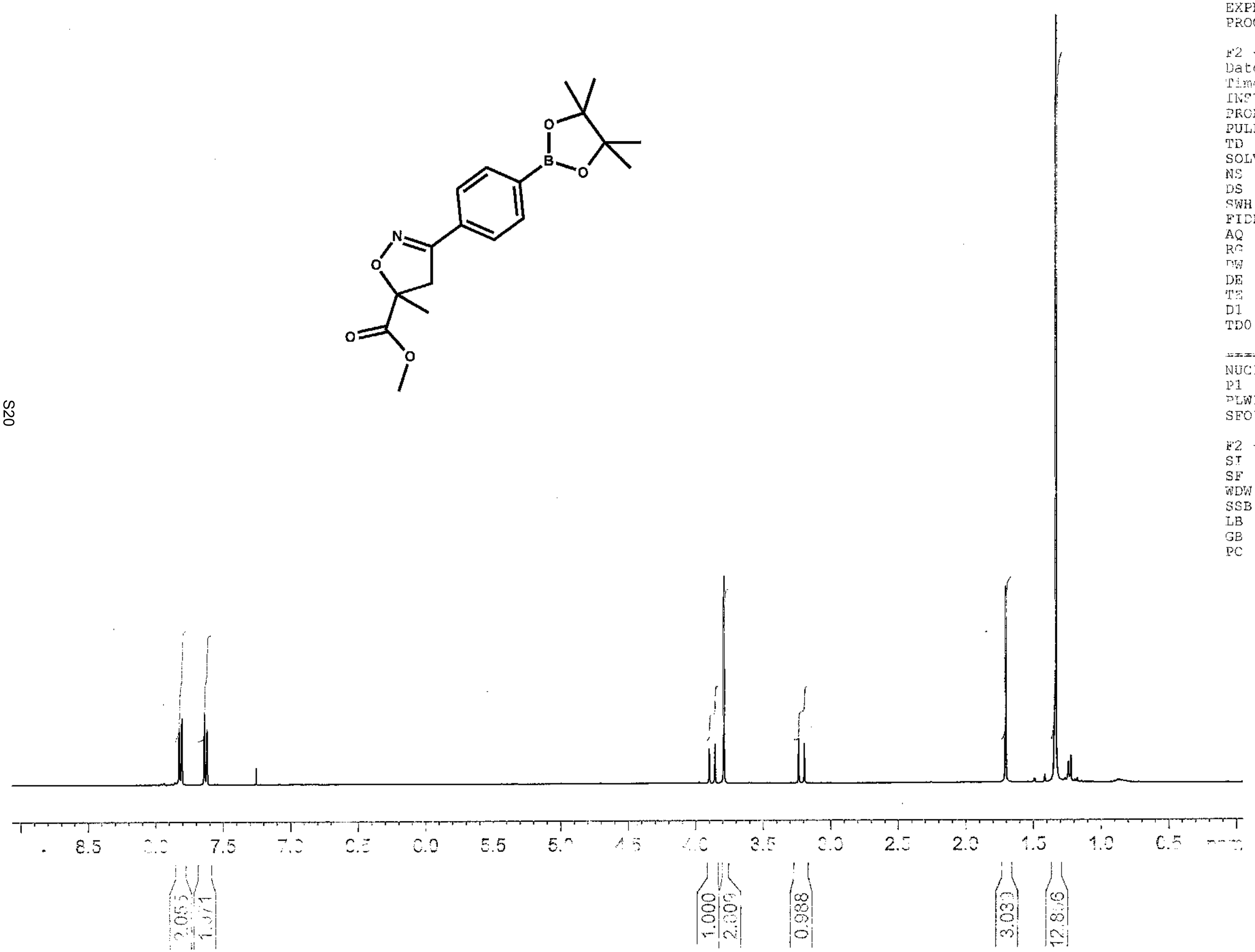

BRUKER

```
Curre?t Dat? ?arameters
NAME        HardingX10209
EXPNO                  10
PROCNO                  1

F2 - Acquisition Parameters
Date_            20111206
Time                1?.21
INSTRUM           Av400X
PROBHD    5 mm PATBO BB-
PULPROG              zg30
TD                  32768
SOLVENT             CDCl3
NS                     32
DS                      2
SWH              6393.862 Hz
FIDRES           0.195125 Hz
AQ              2.5625076 sec
RG                  6?.07
DW                 78.200 usec
DE                   6.50 usec
TE                  297.0 K
D1             1.00000000 sec
TD0                     1

======== CHANNEL f1 ========
NUC1                   1H
P1                  17.00 usec
PLW1          20.00000000 W
SFO1          400.1328?09 MHz

F2 - Processing parameters
SI                  65536
SF            400.1300127 MHz
WDW                    EM
SSB      0
LB                   0.10 Hz
GB       0
PC                   1.00
```

Name Sarah Harding
WBS R-00366-09-003
SLH078df1
CSIRO Av400X_13C CDCl3 C:\\ har97c 32

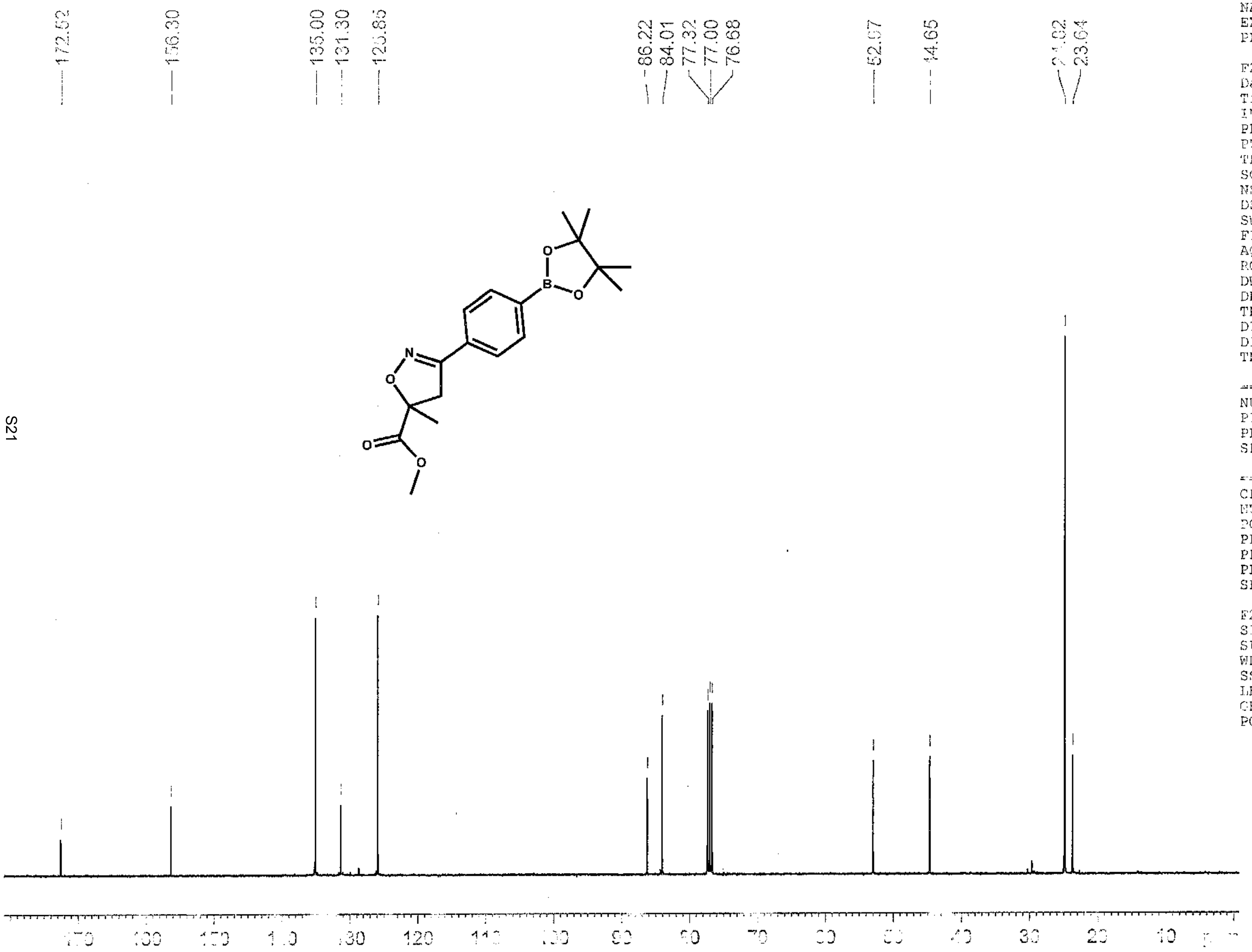

BRUKER

Current Data Parameters
NAME HardingX40209
EXPNO 11
PROCNO 1

F2 - Acquisition Parameters
Date_ 20111206
Time 19.42
INSTRUM Av400X
PROBHD 5 mm PATBO BB-
PULPROG zgpg30
TD 65536
SOLVENT CDCl3
NS 1000
DS 4
SWH 26041.666 Hz
FIDRES 0.397364 Hz
AQ 1.2583412 sec
RG 184.42
DW 19.200 usec
DE 6.50 usec
TE 297.1 K
D1 1.00000000 sec
D11 0.03000000 sec
TD0 1

======== CHANNEL f1 ========
NUC1 13C
P1 10.00 usec
PLW1 67.00000000 W
SFO1 100.6248425 MHz

======== CHANNEL f2 ========
CPDPRG2 bi_waltz65_256
NUC2 1H
PCPD2 90.00 usec
PLW2 20.00000000 W
PLW12 0.71358001 W
PLW13 0.57800001 W
SFO2 400.1316005 MHz

F2 - Processing parameters
SI 32768
SF 100.6127749 MHz
WDW EM
SSB 0
LB 1.00 Hz
GB 0
PC 1.40

Name Sarah Harding
WBS R-00366-09-003
SLH078df1
CSIRO Av400X_cosy CDCl3 C:\\ har97c 32

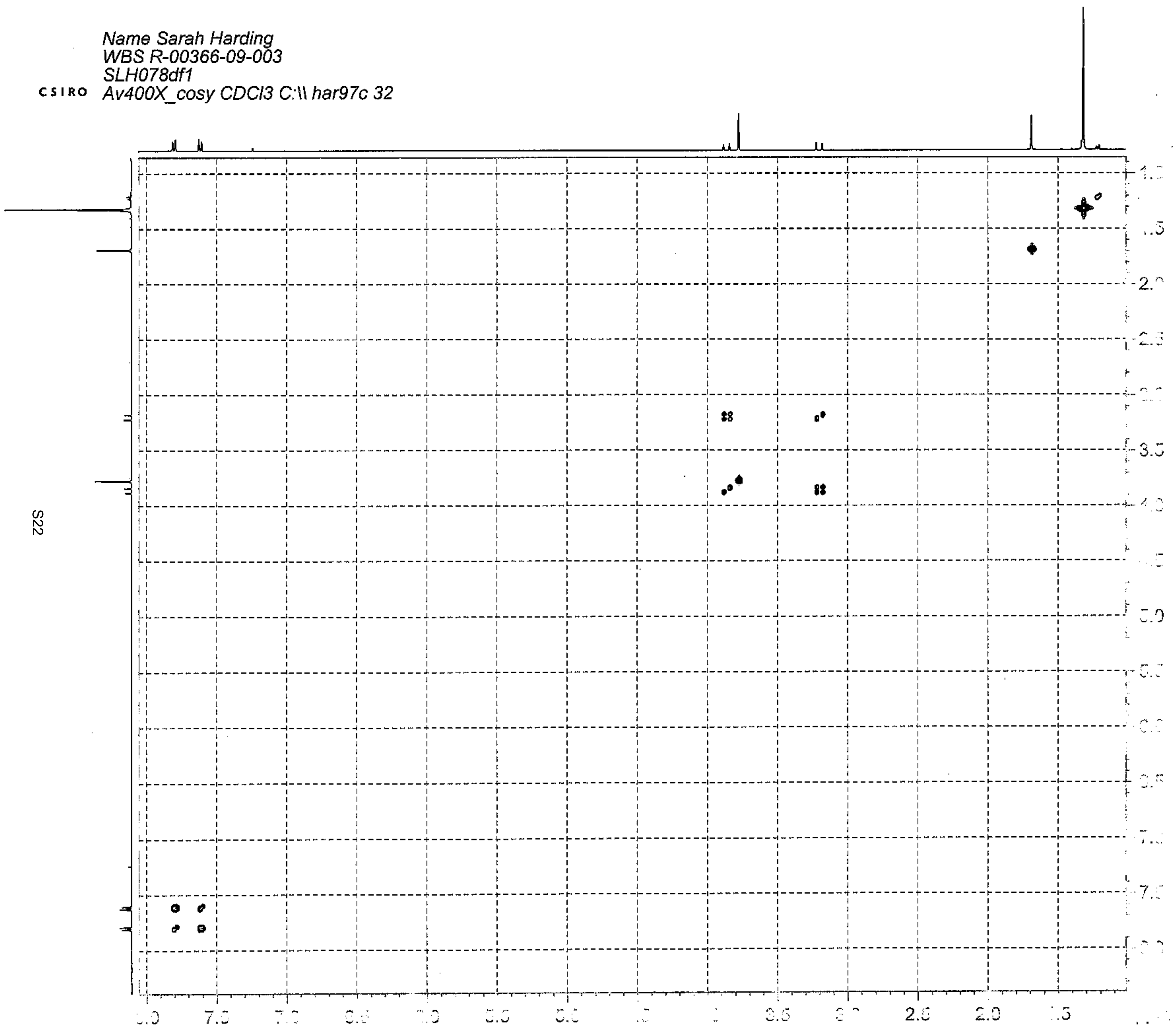

BRUKER

Current Data Parameters
NAME HardingX40209
EXPNO 12
PROCNO 1

F2 - Acquisition Parameters
Date_ 20111206
Time 19.43
INSTRUM Av400X
PROBHD 5 mm PATBO BB-
PULPROG cosygpqf45_CSIRO
TD 2048
SOLVENT CDCl3
NS 1
DS 16
SWH 4807.692 Hz
FIDRES 2.347506 Hz
AQ 0.2130420 sec
RG [illegible]
DW 104.000 usec
DE 6.50 usec
TE 296.8 K
D0 0.00000300 sec
D1 1.00000000 sec
D13 0.00000400 sec
D16 0.00020000 sec
IN0 0.00020800 sec

======== CHANNEL f1 ========
NUC1 1H
P0 8.50 usec
P1 17.00 usec
PLW1 20.00000000 W
SFO1 400.1322007 MHz

====== GRADIENT CHANNEL =====
GPNAM1 smsq10.100
GPZ1 10.00 %
P16 1000.00 usec

F1 - Acquisition parameters
TD 512
SFO1 400.1322 MHz
FIDRES 9.390023 Hz
SW 12.015 ppm
FnMODE QF

F2 - Processing parameters
SI 1024
SF 400.1300178 MHz
WDW SINE
SSB 0
LB 0 Hz
GB 0
PC 1.00

F1 - Processing parameters
SI 1024
MC2 QF
SF 400.1300178 MHz
WDW
SSB 0
LB 0 Hz
GB 0

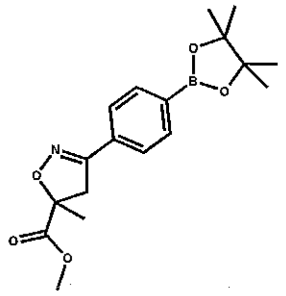

CSIRO

CSIRO Materials Science & Engineering
S.Harding

Accurate Mass Report
SLH078D

Low Resolution EI Spectrum

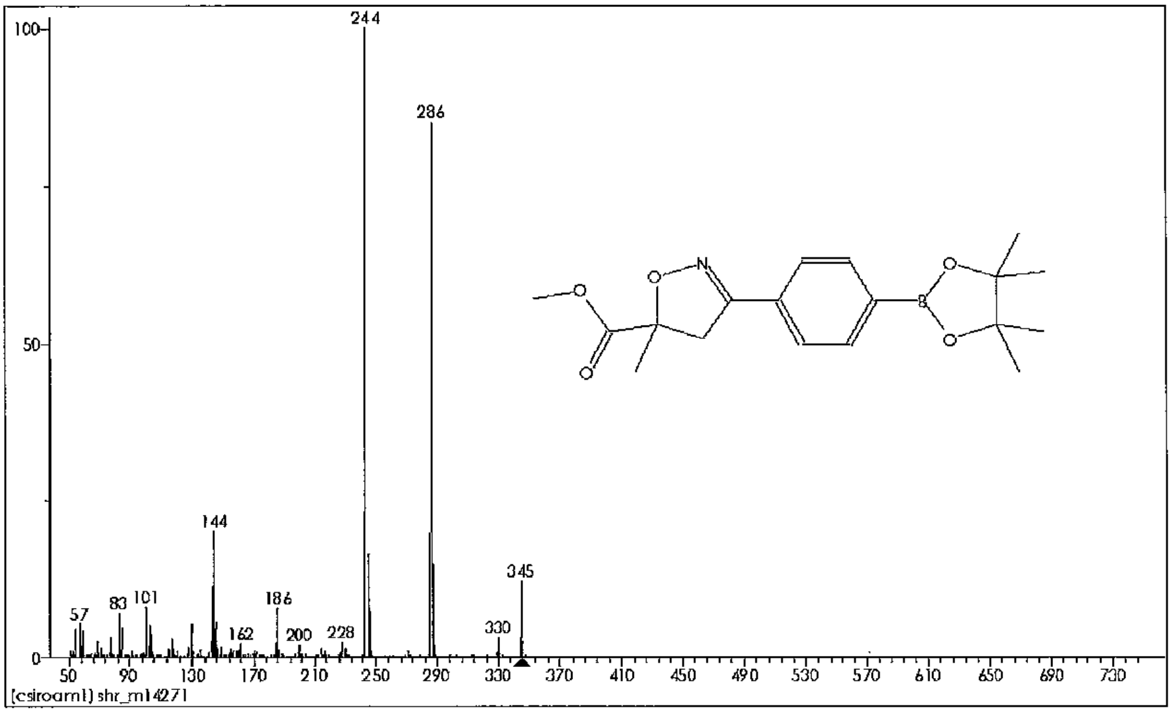

High Resolution EI Spectrum

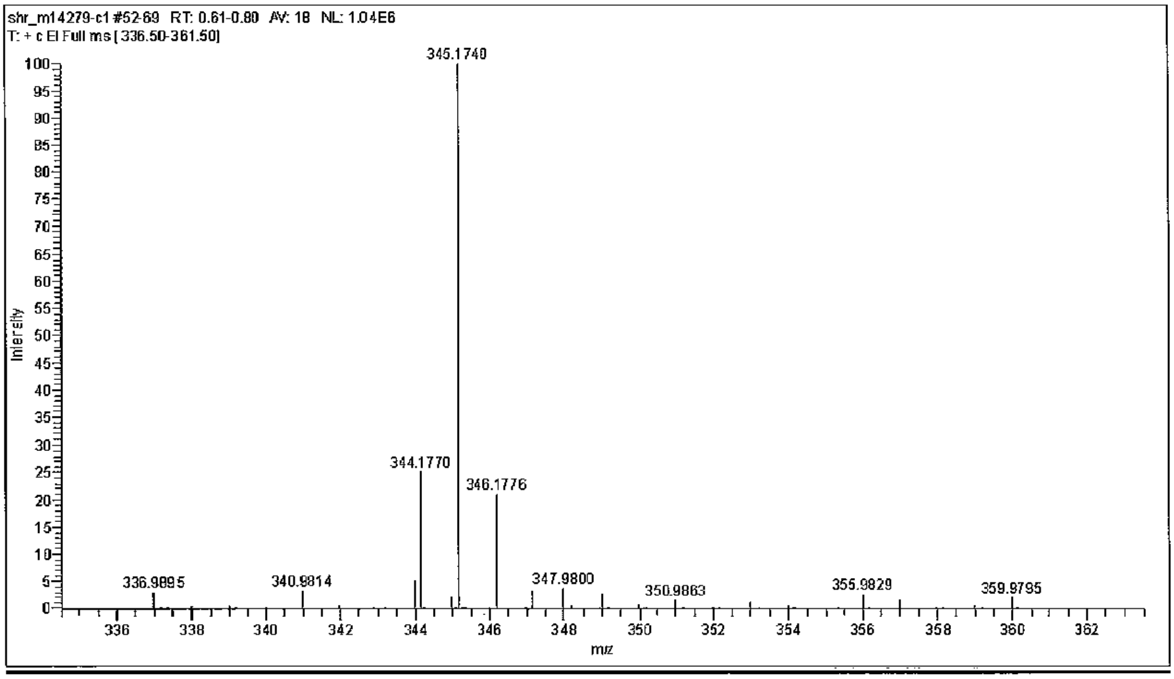

CSIRO Name Sarah Harding
WBS R-00366-09-003
SLH079hf1
Av400X_1H CDCl3 C:\\ har97c 7

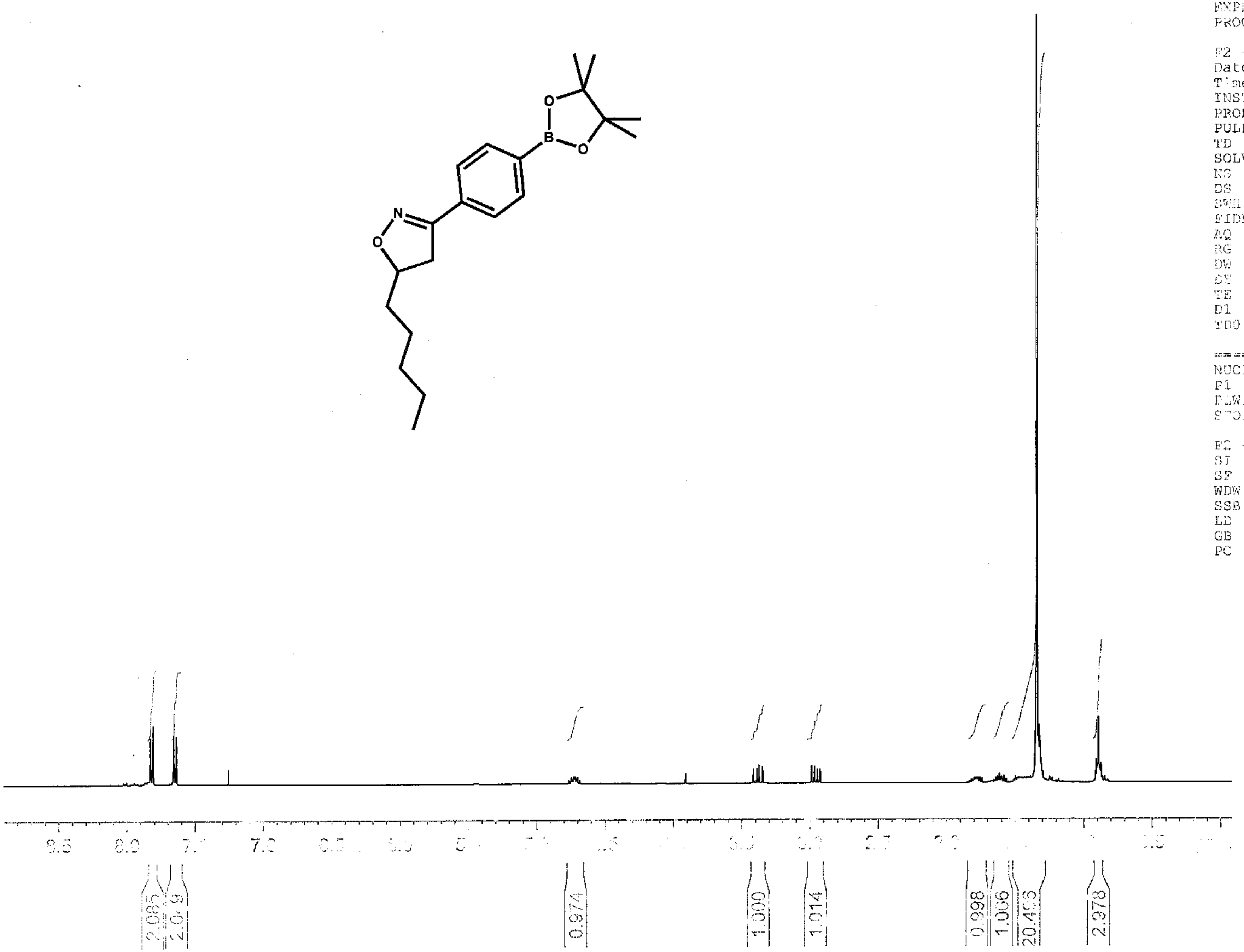

BRUKER

```
Current Data Parameters
NAME      HardingX40214
EXPNO                10
PROCNO                1

F2 - Acquisition Parameters
Date_          20111207
Time              16.55
INSTRUM          Av400X
PROBHD   5 mm PATBO BB-
PULPROG            zg30
TD                32768
SOLVENT           CDCl3
NS                   32
DS                    2
SWH            6393.862 Hz
FIDRES         0.195125 Hz
AQ            2.5625076 sec
RG                68.07
DW               78.200 usec
DE                 6.50 usec
TE                297.0 K
D1           1.00000000 sec
TD0                   1

======== CHANNEL f1 ========
NUC1                 1H
P1                17.00 usec
PLW1        20.00000000 W
SFO1        400.1328009 MHz

F2 - Processing parameters
SI                65536
SF          400.1300127 MHz
WDW                  EM
SSB      0
LB                 0.10 Hz
GB       0
PC                 1.00
```

CSIRO
Name Sarah Harding
WBS R-00366-09-003
SLH079hf1
Av400X_13C CDCl3 C:\\ har97c 7

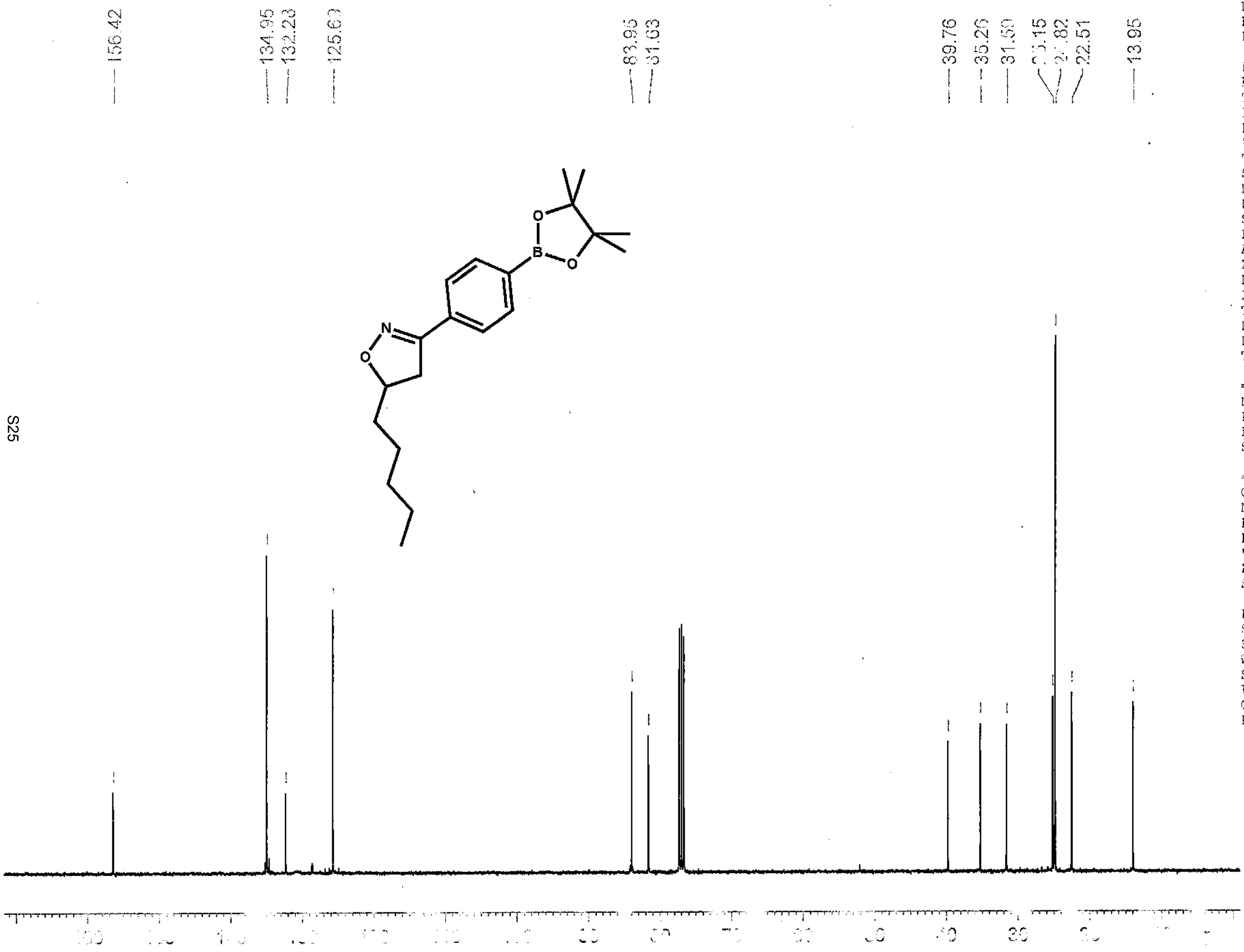

BRUKER

Current Data Parameters
NAME Hardin[illegible]
EXPNO 11
PROCNO 1

F2 - Acquisition Parameters
Date_ 20111207
Time 18.11
INSTRUM A.400X
PROBHD 5 mm PATBO BB-
PULPROG zgpg30
TD 65536
SOLVENT CDCl3
NS 750
DS 4
SWH 26041.666 Hz
FIDRES 0.397364 Hz
AQ 1.2583412 sec
RG 184.42
DW 19.200 usec
DE 6.50 usec
TE 297.0 K
D1 1.00000000 sec
D11 0.03000000 sec
TD0 1

======== CHANNEL f1 ========
NUC1 13C
P1 10.00 usec
PLW1 67.00000000 W
SFO1 100.6248425 MHz

======== CHANNEL f2 ========
CPDPRG2 bi_waltz65_256
NUC2 1H
PCPD2 90.00 usec
PLW2 20.00000000 W
PLW12 0.71358001 W
PLW13 0.57800001 W
SFO2 400.1316005 MHz

F2 - Processing parameters
SI 32768
SF 100.6127746 MHz
WDW EM
SSB 0
LB 1.00 Hz
GB 0
PC 1.40

Name Sarah Harding
WBS R-00366-09-003
SLH079hf1
CSIRO Av400X_cosy CDCl3 C:\\ har97c 7

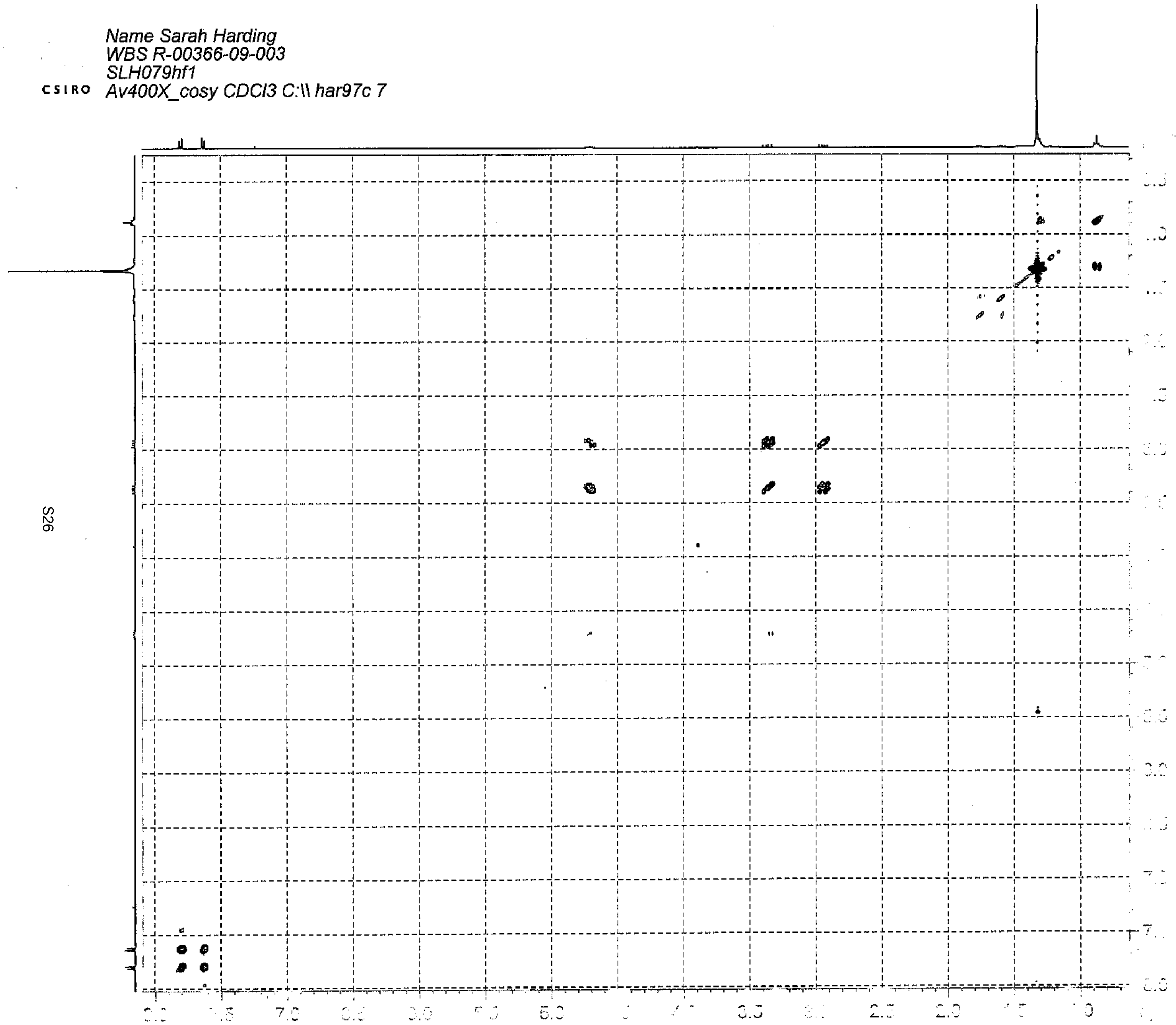

BRUKER

Current Data Parameters
NAME HardingX40714
EXPNO 12
PROCNO 1

F2 - Acquisition Parameters
Date_ 20111207
Time 16.12
INSTRUM Av400X
PROBHD 5 mm PATBO BB-
PULPROG cosygpqf45 CSIRO
TD 2048
SOLVENT CDCl3
NS 1
DS 16
SWH 4807.692 Hz
FIDRES 2.347506 Hz
AQ 0.2130420 sec
RG 181.42
DW 104.000 usec
DE 6.50 usec
TE 296.7 K
D0 0.00000300 sec
D1 1.00000000 sec
D12 0.00002000 sec
D16 0.00020000 sec
IN0 0.00020800 sec

======== CHANNEL f1 ========
NUC1 1H
P0 8.50 usec
P1 17.00 usec
PLW1 20.00000000 W
SFO1 400.1322007 MHz

======== GRADIENT CHANNEL ========
GPNAM1 sms q10.100
GPZ1 10.00 %
P16 1000.00 usec

F1 - Acquisition parameters
TD 512
SFO1 400.1322 MHz
FIDRES 9.390023 Hz
SW 12.015 ppm
FnMODE QF

F2 - Processing parameters
SI 1024
SF 400.1300179 MHz
WDW SINE
SSB 0
LB 0 Hz
GB 0
PC 1.00

F1 - Processing parameters
SI 1024
MC2 QF
SF 400.1[illegible]0176 MHz
WDW
SSB 0
LB 0 Hz
GB 0

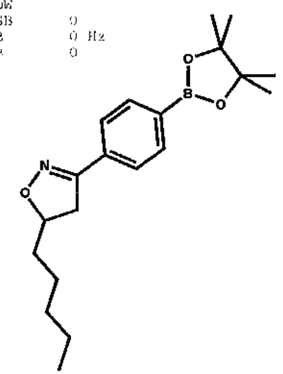

13/12/2011

CSIRO Materials Science & Engineering — Accurate Mass Report

S.Harding — SLH079hf1 H

CSIRO

Low Resolution EI Spectrum

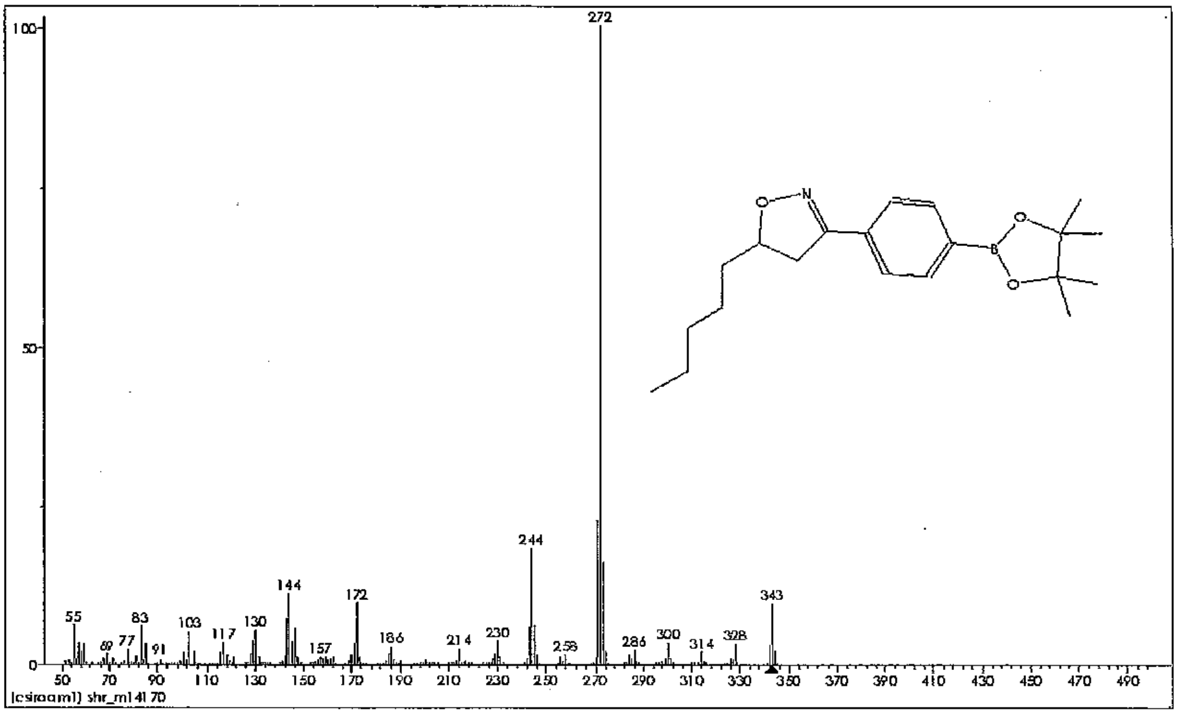

High Resolution EI Spectrum

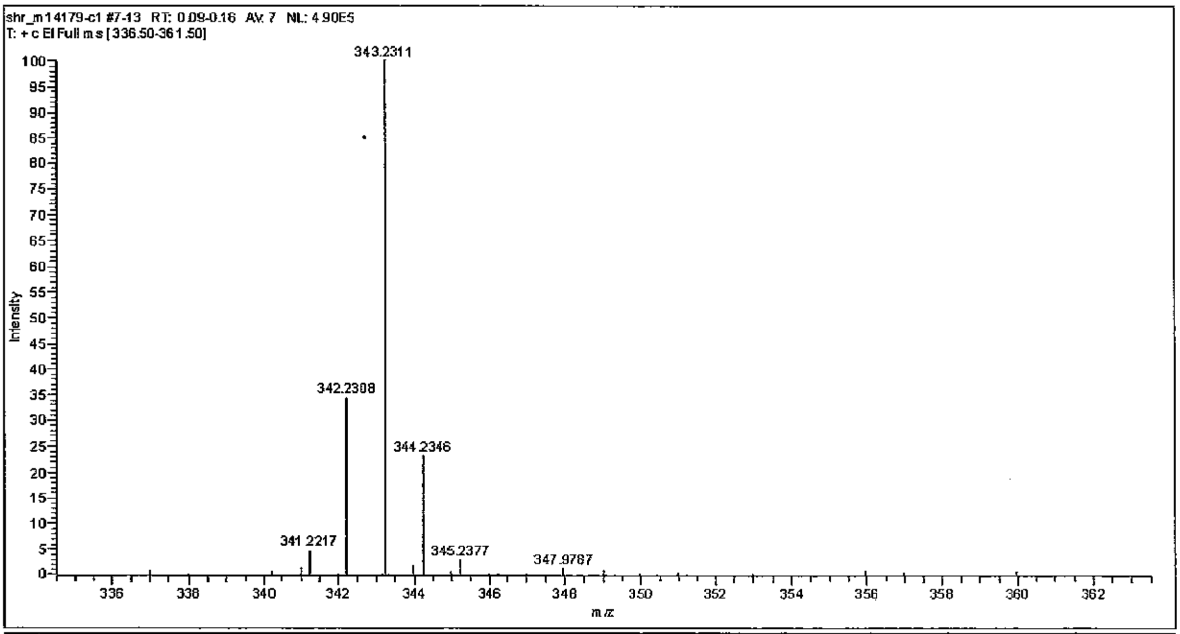

Australian Science, Australia's Future

Name Sarah Harding
WBS R-00366-09-003
SLH084-2d-alpha-f1
CSIRO Av400X_1H CDCl3 C:\\ har97c 22

BRUKER

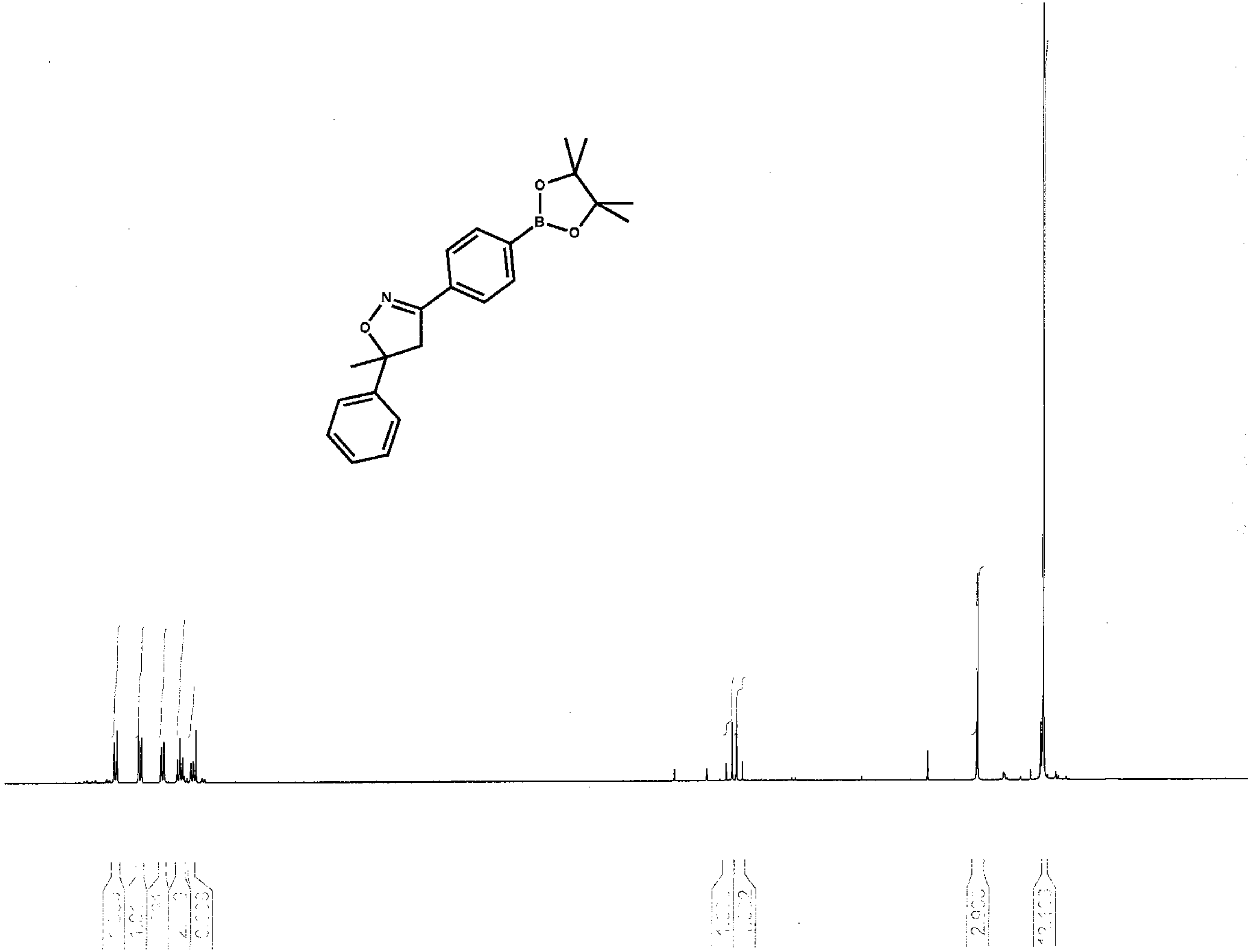

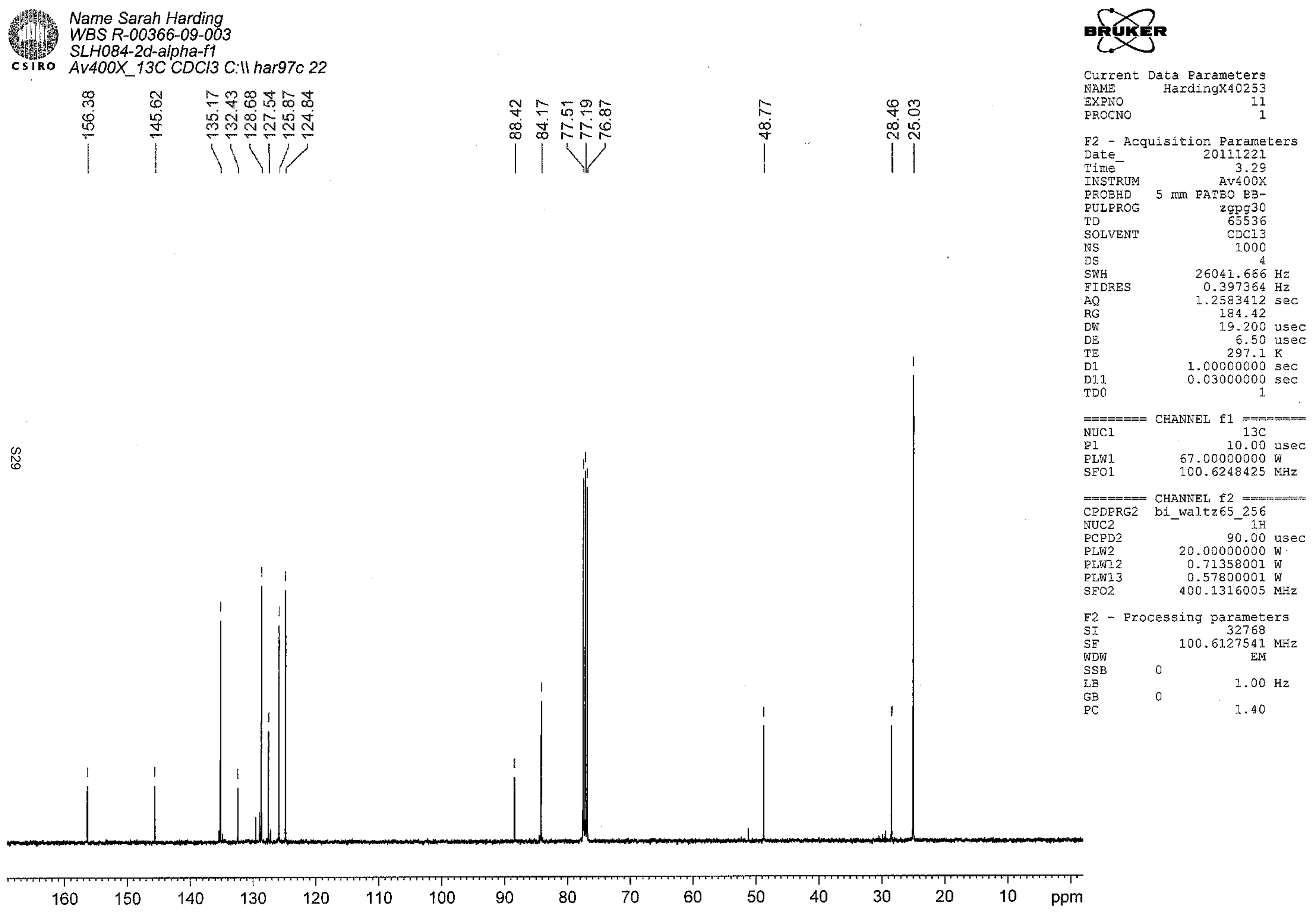

Name Sarah Harding
WBS R-00366-09-003
SLH084-2d-alpha-f1
Av400X_13C CDCl3 C:\\ har97c 22
156.38
145.62
135.17
132.43
128.68
127.54
125.87
124.84
88.42
84.17
77.51
77.19
76.87
48.77
28.46
25.03
160 150 140 130 120 110 100 90 80 70 60 50 40 30 20 10 ppm
Current Data Parameters
NAME HardingX40253
EXPNO 11
PROCNO 1
F2 - Acquisition Parameters
Date_ 20111221
Time_ 3.29
INSTRUM Av400X
PROBHD 5 mm PATBO BB-
PULPROG zgpg30
TD 65536
SOLVENT CDCl3
NS 1000
DS 4
SWH 26041.666 Hz
FIDRES 0.397364 Hz
AQ 1.2583412 sec
RG 184.42
DW 19.200 usec
DE 6.50 usec
TE 297.1 K
D1 1.00000000 sec
D11 0.03000000 sec
TD0 1
======== CHANNEL f1 ========
NUC1 13C
P1 10.00 usec
PLW1 67.00000000 W
SFO1 100.6248425 MHz
======== CHANNEL f2 ========
CPDPRG2 bi_waltz65_256
NUC2 1H
PCPD2 90.00 usec
PLW2 20.00000000 W
PLW12 0.71358001 W
PLW13 0.57800001 W
SFO2 400.1316005 MHz
F2 - Processing parameters
SI 32768
SF 100.6127541 MHz
WDW EM
SSB 0
LB 1.00 Hz
GB 0
PC 1.40

CSIRO

CSIRO Materials Science & Engineering
S.Harding

Accurate Mass Report
SLH084-2d-alpha

Low Resolution EI Spectrum

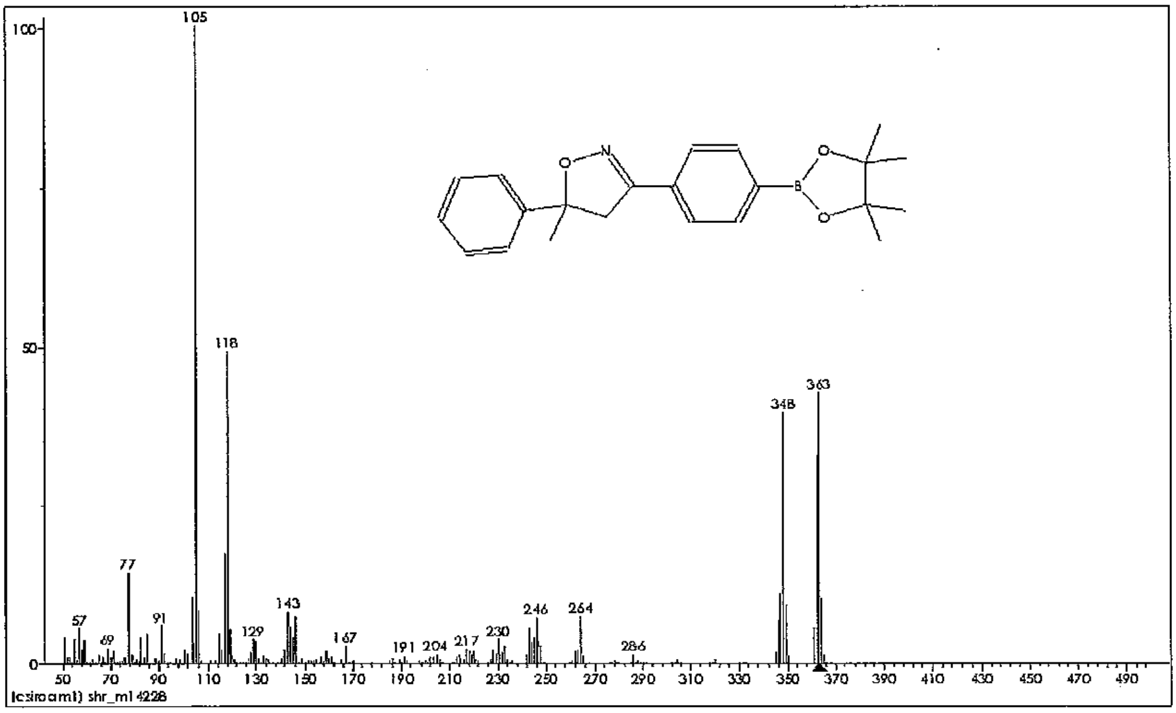

High Resolution EI Spectrum

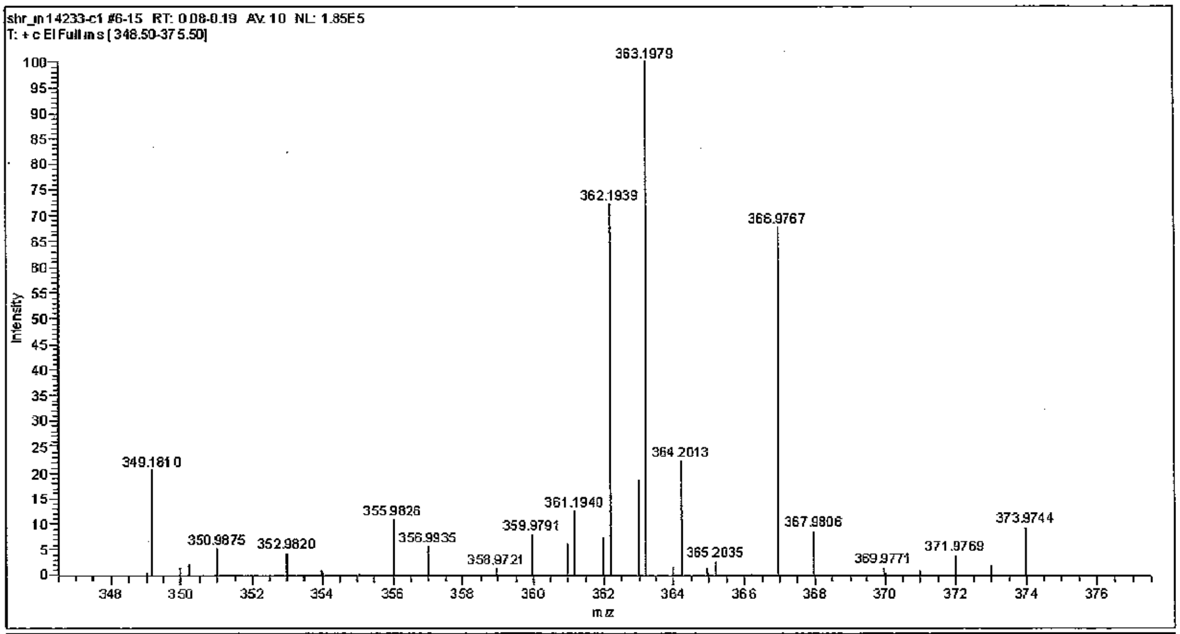

Name Sarah Harding
WBS R-00366-09-003
SLH084-2e-beta-f1
Av400X_1H CDCl3 C:\\ har97c 19

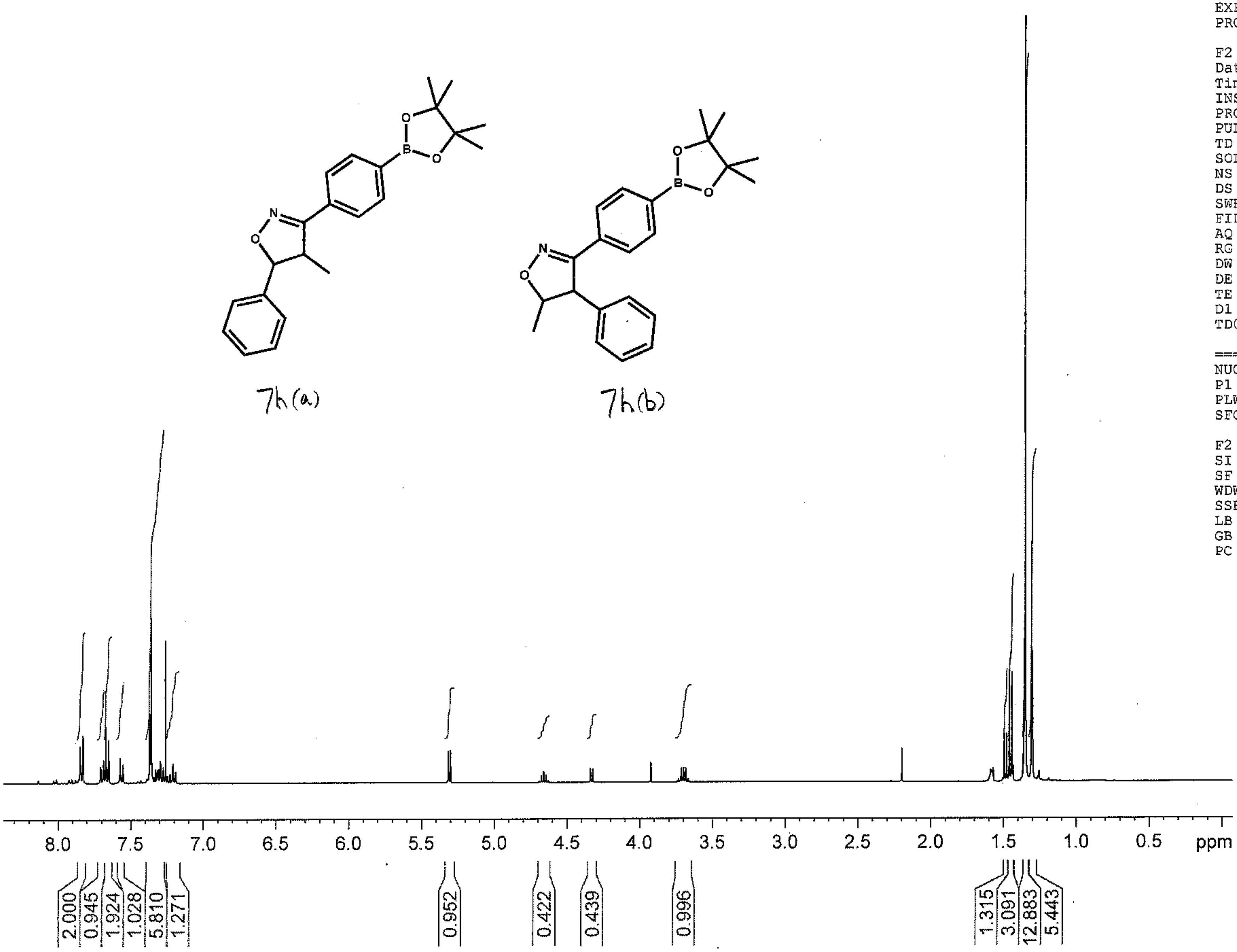


```
Current Data Parameters
NAME        HardingX40255
EXPNO                  10
PROCNO                  1

F2 - Acquisition Parameters
Date_            20111220
Time                16.32
INSTRUM           Av400X
PROBHD   5 mm PATBO BB-
PULPROG              zg30
TD                  32768
SOLVENT             CDCl3
NS                     32
DS                      2
SWH              6393.862 Hz
FIDRES           0.195125 Hz
AQ              2.5625076 sec
RG                 184.42
DW                 78.200 usec
DE                   6.50 usec
TE                  297.1 K
D1             1.00000000 sec
TD0                     1

======== CHANNEL f1 ========
NUC1                   1H
P1                  17.00 usec
PLW1          20.00000000 W
SFO1          400.1328009 MHz

F2 - Processing parameters
SI                  65536
SF            400.1300101 MHz
WDW                    EM
SSB      0
LB                   0.10 Hz
GB       0
PC                   1.00
```

Name Sarah Harding
WBS R-00366-09-003
SLH084-2e-beta-f1
Av400X_13C CDCl3 C:\\ har97c 19

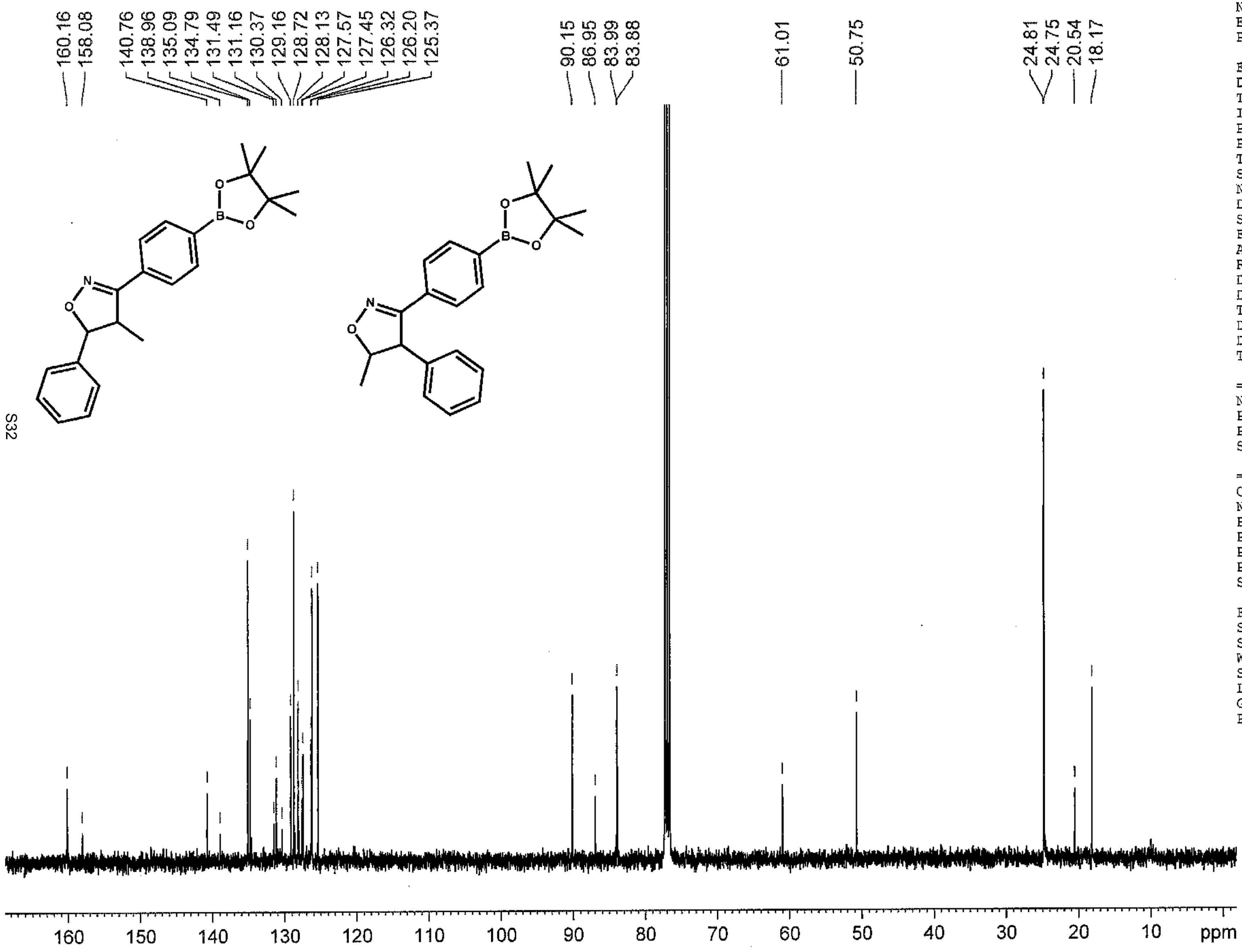


```
Current Data Parameters
NAME        HardingX40255
EXPNO                  11
PROCNO                  1

F2 - Acquisition Parameters
Date_            20111221
Time                 4.41
INSTRUM            Av400X
PROBHD    5 mm PATBO BB-
PULPROG           zgpg30
TD                 65536
SOLVENT            CDCl3
NS                  1000
DS                     4
SWH            26041.666 Hz
FIDRES          0.397364 Hz
AQ             1.2583412 sec
RG                184.42
DW                19.200 usec
DE                  6.50 usec
TE                 297.1 K
D1            1.00000000 sec
D11           0.03000000 sec
TD0                    1

======== CHANNEL f1 ========
NUC1                 13C
P1                 10.00 usec
PLW1         67.00000000 W
SFO1         100.6248425 MHz

======== CHANNEL f2 ========
CPDPRG2   bi_waltz65_256
NUC2                  1H
PCPD2              90.00 usec
PLW2         20.00000000 W
PLW12         0.71358001 W
PLW13         0.57800001 W
SFO2         400.1316005 MHz

F2 - Processing parameters
SI                 32768
SF           100.6127746 MHz
WDW                   EM
SSB      0
LB                  1.00 Hz
GB       0
PC                  1.40
```

Name Sarah Harding
WBS R-00366-09-003
SLH084-2e-beta-f1
Av400X_cosy CDCl3 C:\\ har97c 19

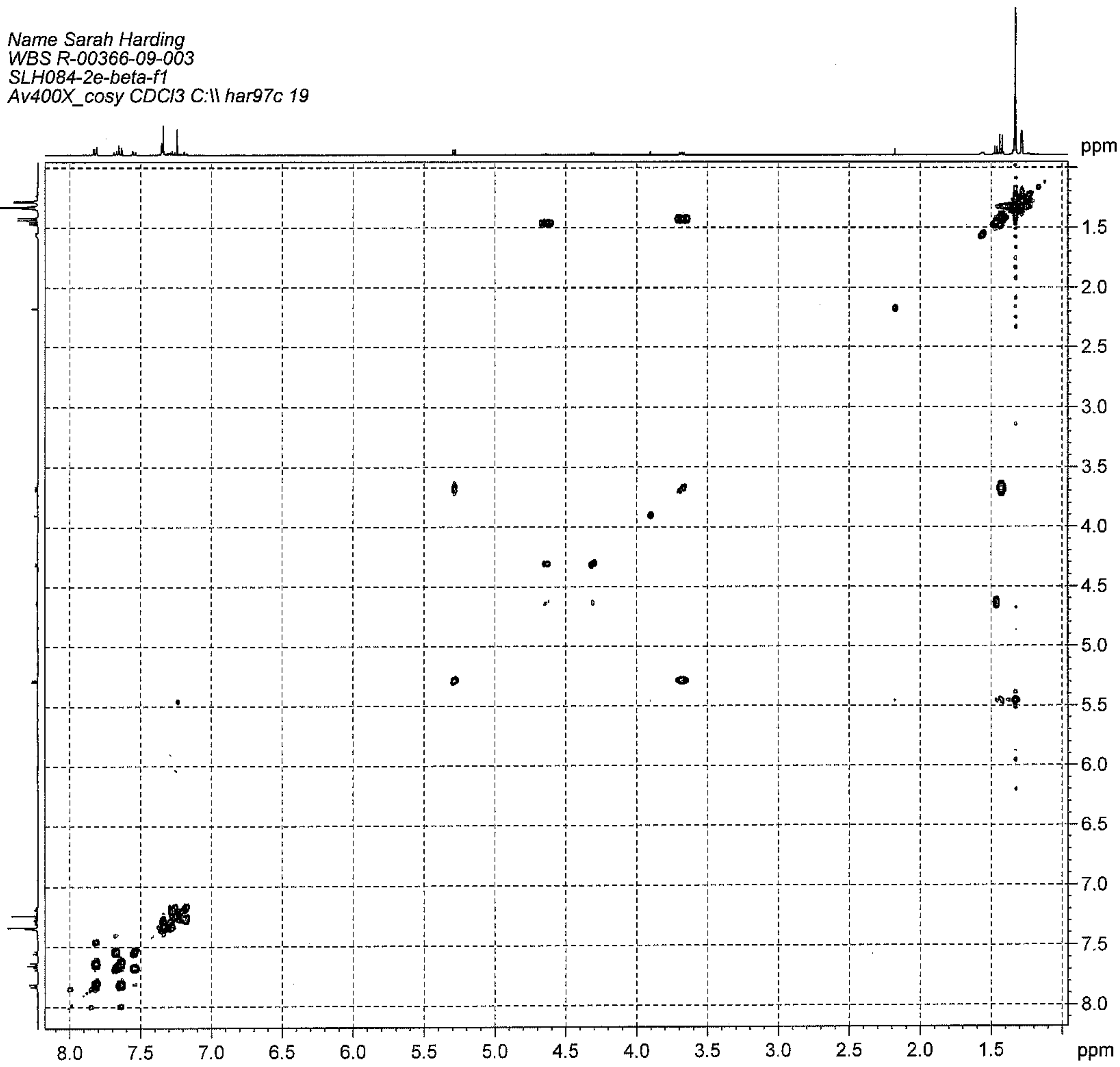


```
Current Data Parameters
NAME        HardingX40255
EXPNO                  12
PROCNO                  1

F2 - Acquisition Parameters
Date_            20111221
Time                 4.42
INSTRUM            Av400X
PROBHD     5 mm PATBO BB-
PULPROG  cosygpqf45_CSIRO
TD                   2048
SOLVENT             CDCl3
NS                      1
DS                     16
SWH              4807.692 Hz
FIDRES           2.347506 Hz
AQ              0.2130420 sec
RG                 184.42
DW                104.000 usec
DE                   6.50 usec
TE                  297.0 K
D0             0.00000300 sec
D1             1.00000000 sec
D13            0.00000400 sec
D16            0.00020000 sec
IN0            0.00020800 sec

======== CHANNEL f1 ========
NUC1                   1H
P0                   8.50 usec
P1                  17.00 usec
PLW1          20.00000000 W
SFO1          400.1322007 MHz

====== GRADIENT CHANNEL =====
GPNAM1         smsq10.100
GPZ1                10.00 %
P16               1000.00 usec

F1 - Acquisition parameters
TD                    512
SFO1             400.1322 MHz
FIDRES           9.390023 Hz
SW                 12.015 ppm
FnMODE                 QF

F2 - Processing parameters
SI                   1024
SF            400.1300178 MHz
WDW                  SINE
SSB      0
LB       0 Hz
GB       0
PC                   1.00

F1 - Processing parameters
SI                   1024
MC2                    QF
SF            400.1300178 MHz
WDW
SSB      0
LB       0 Hz
GB       0
```

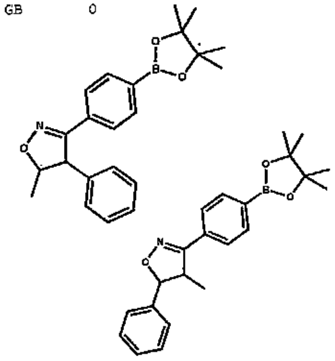

CSIRO Materials Science & Engineering
S.Harding

Accurate Mass Report
SLH084-2e-beta

CSIRO

Low Resolution EI Spectrum

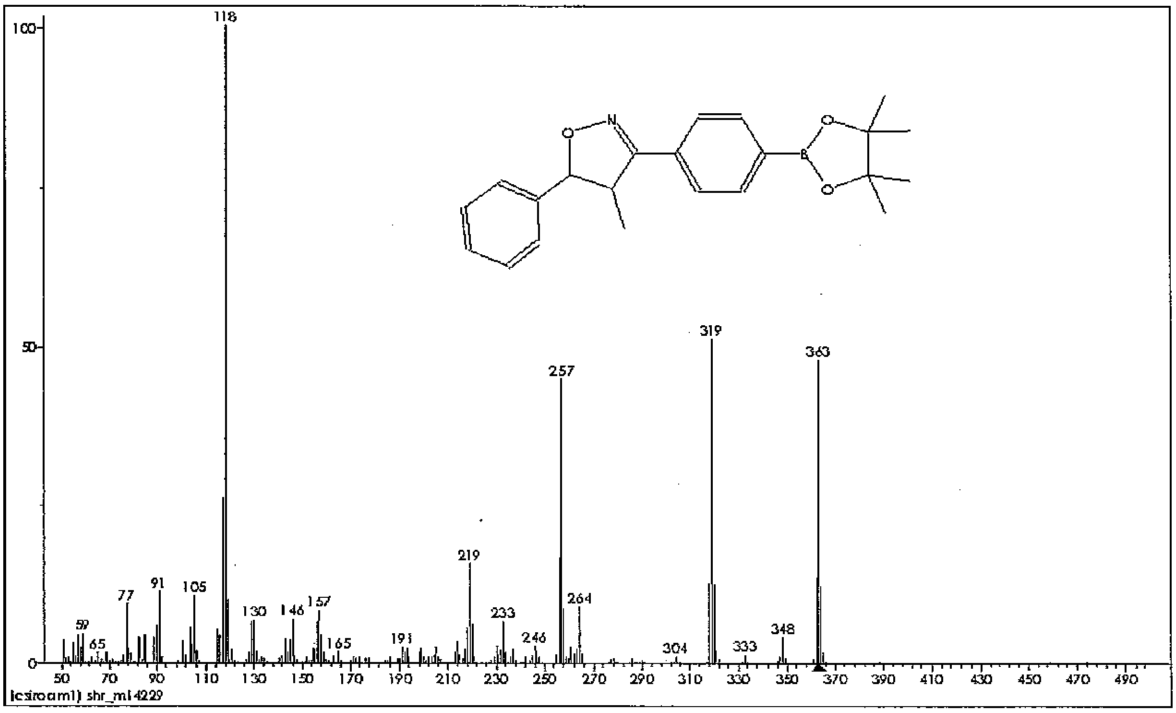

High Resolution EI Spectrum

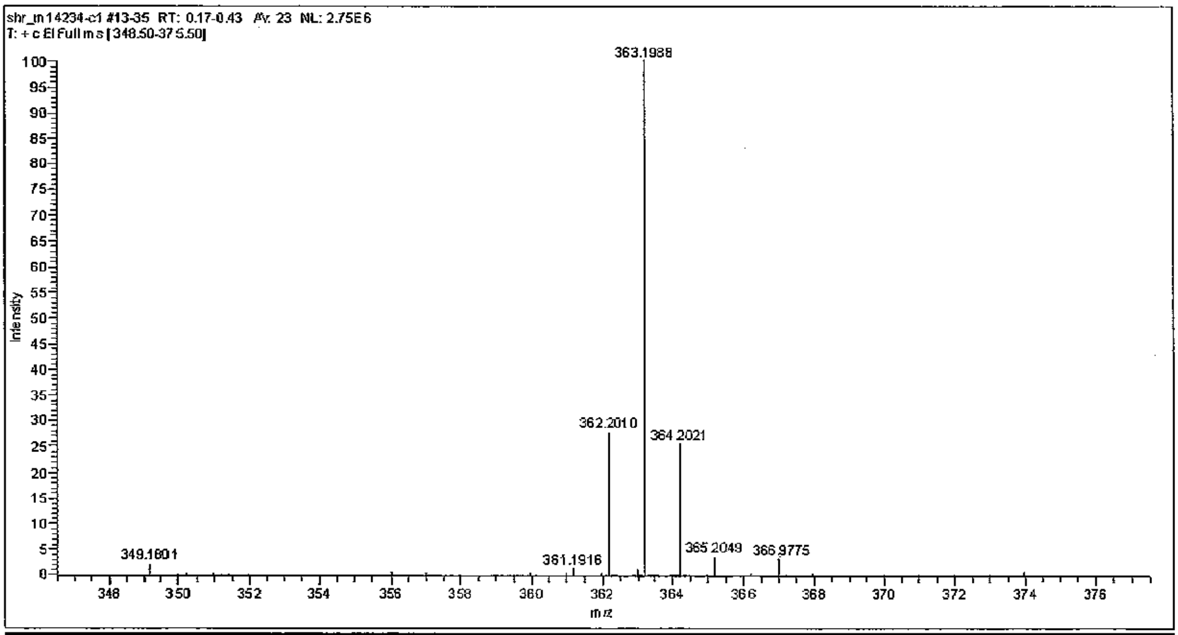

Name Sarah Harding
WBS R-00366-09-003
SLH085-2f-f2
CSIRO Av400X_1H CDCl3 C:\\ har97c 6

BRUKER

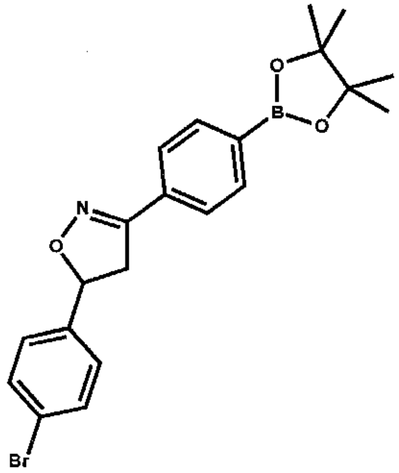

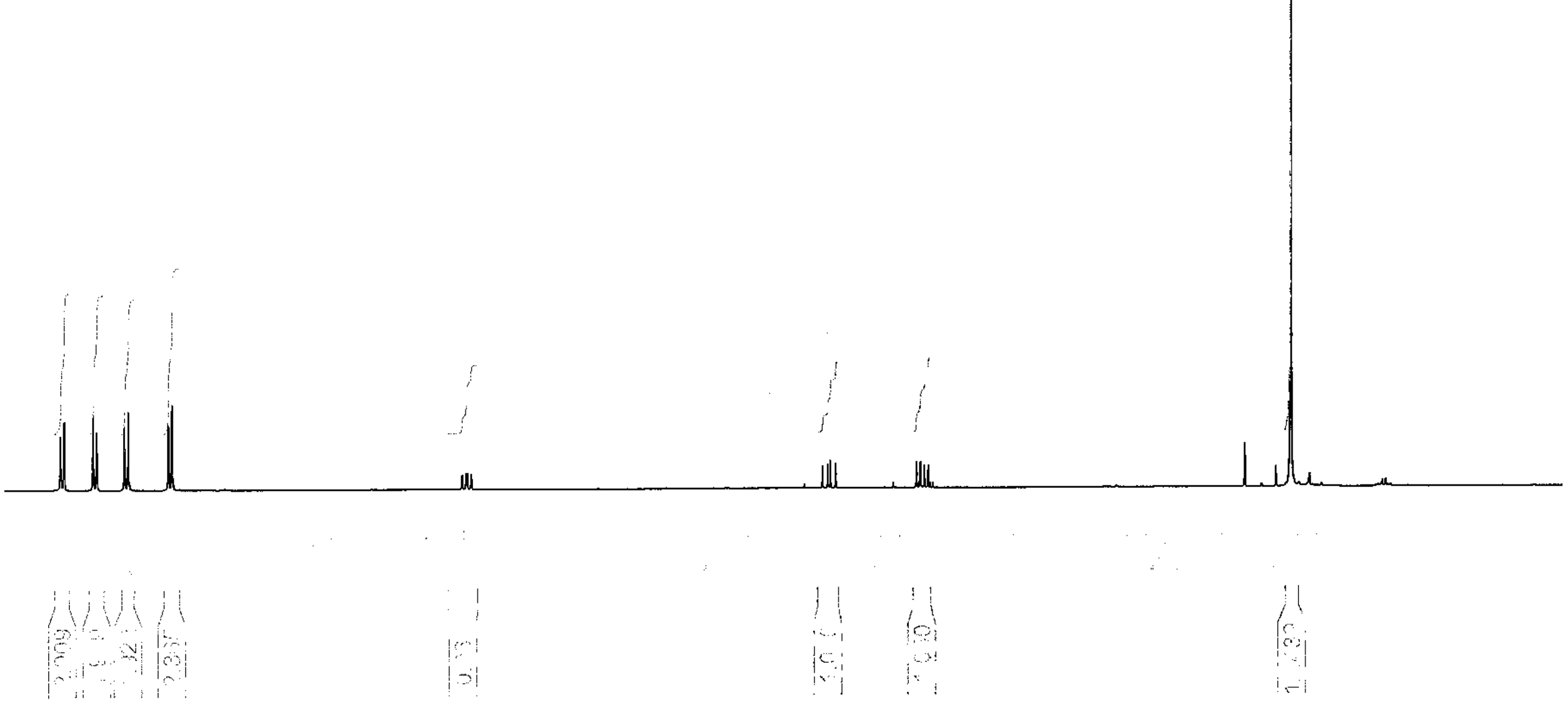

Name Sarah Harding
WBS R-00366-09-003
SLH085-2f-f2
Av400X_13C CDCl3 C:\\ har97c 6

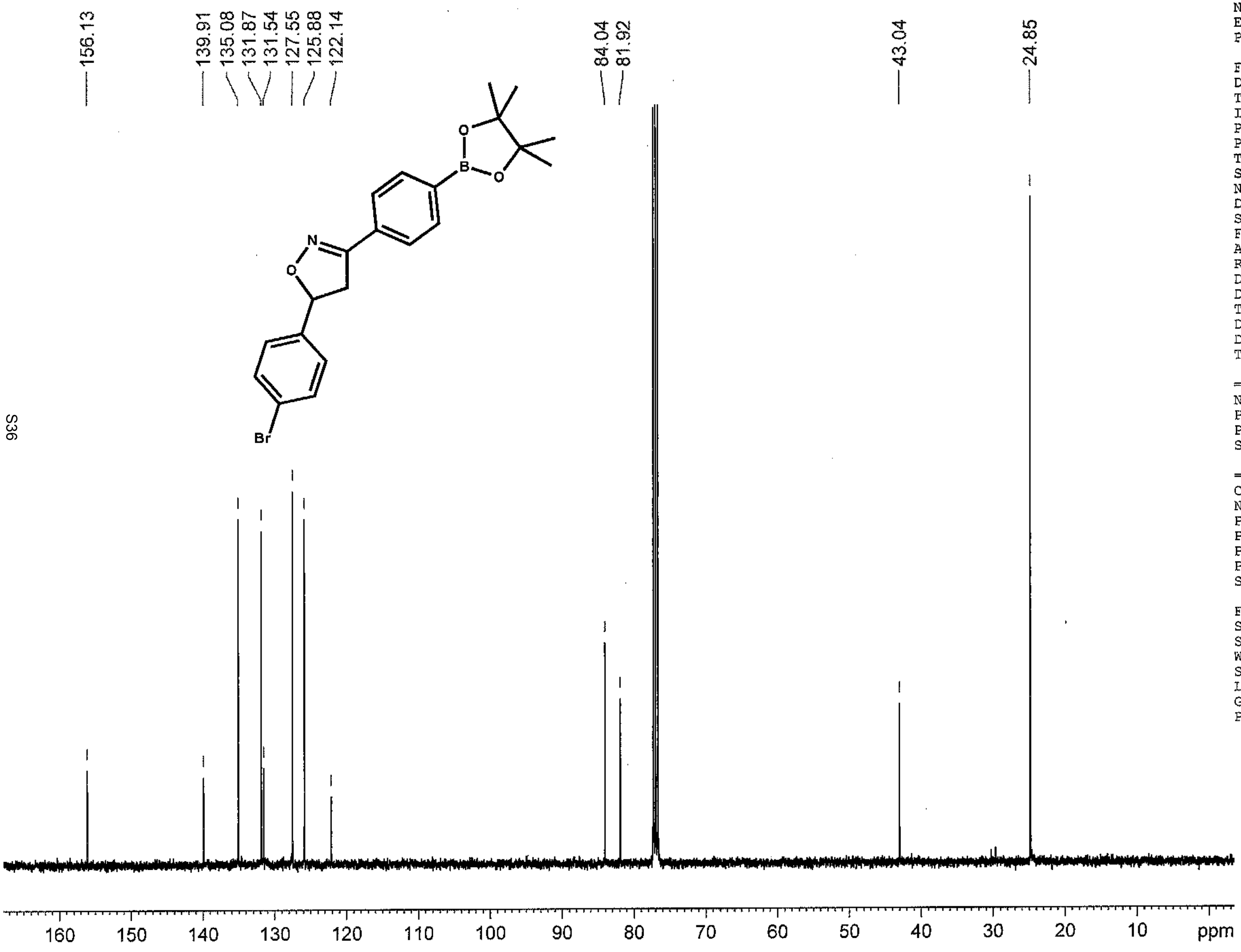


```
Current Data Parameters
NAME          HardingX40266
EXPNO                    11
PROCNO                    1

F2 - Acquisition Parameters
Date_              20111222
Time                  12.15
INSTRUM              Av400X
PROBHD     5 mm PATBO BB-
PULPROG             zgpg30
TD                    65536
SOLVENT               CDCl3
NS                      500
DS                        4
SWH               26041.666 Hz
FIDRES             0.397364 Hz
AQ                1.2583412 sec
RG                   184.42
DW                   19.200 usec
DE                     6.50 usec
TE                    297.1 K
D1               1.00000000 sec
D11              0.03000000 sec
TD0                       1

======== CHANNEL f1 ========
NUC1                    13C
P1                    10.00 usec
PLW1            67.00000000 W
SFO1            100.6248425 MHz

======== CHANNEL f2 ========
CPDPRG2     bi_waltz65_256
NUC2                     1H
PCPD2                 90.00 usec
PLW2            20.00000000 W
PLW12            0.71358001 W
PLW13            0.57800001 W
SFO2            400.1316005 MHz

F2 - Processing parameters
SI                    32768
SF              100.6127731 MHz
WDW                      EM
SSB        0
LB                     1.00 Hz
GB         0
PC                     1.40
```

CSIRO Materials Science & Engineering
S.Harding

Accurate Mass Report
SLH085F

CSIRO

Low Resolution EI Spectrum

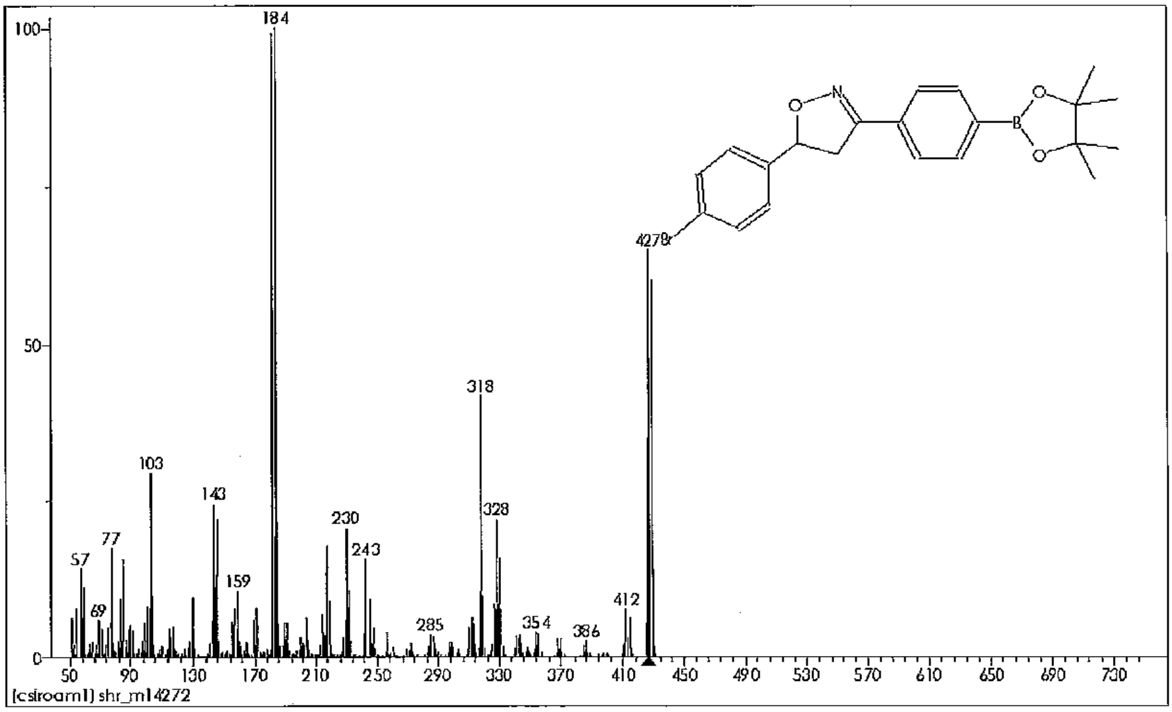

High Resolution EI Spectrum

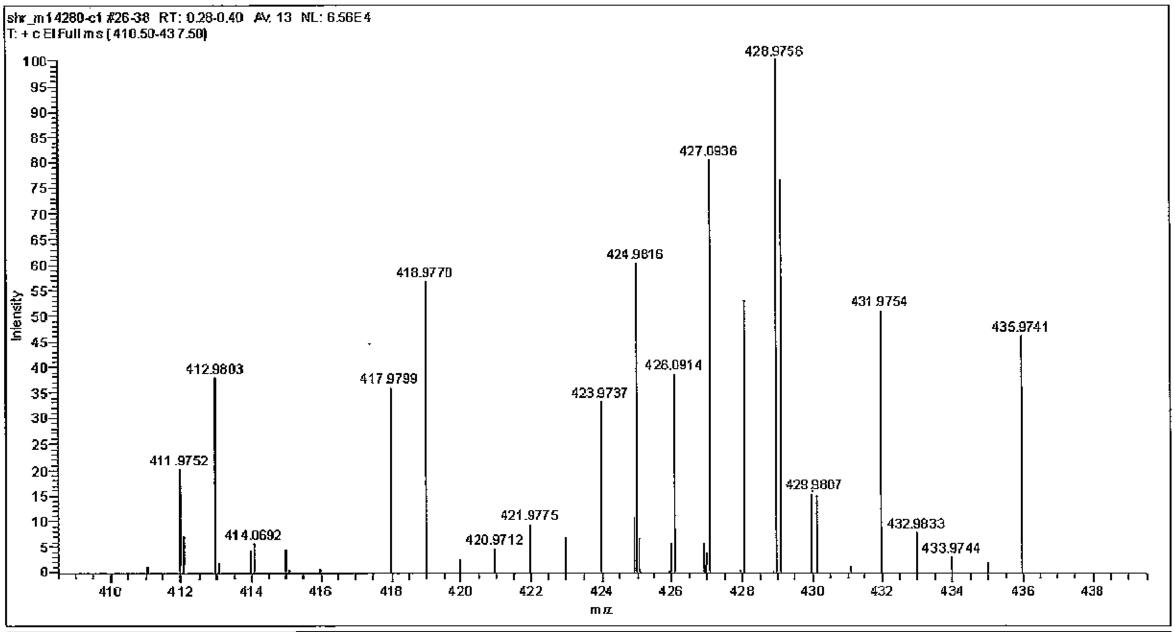

CSIRO

Name Sarah Harding
WBS R-00366-09-003
SLH074s A
Av400X_1H CDCl3 C:\\ har97c 8

BRUKER

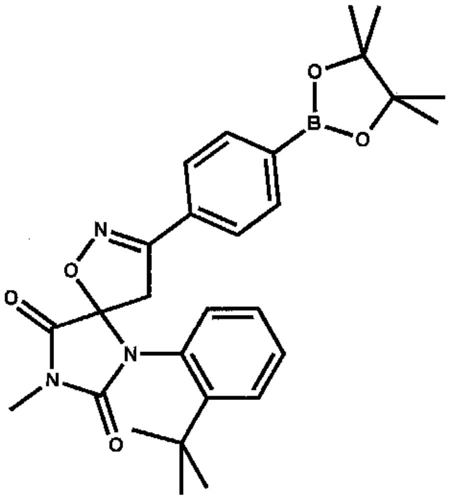

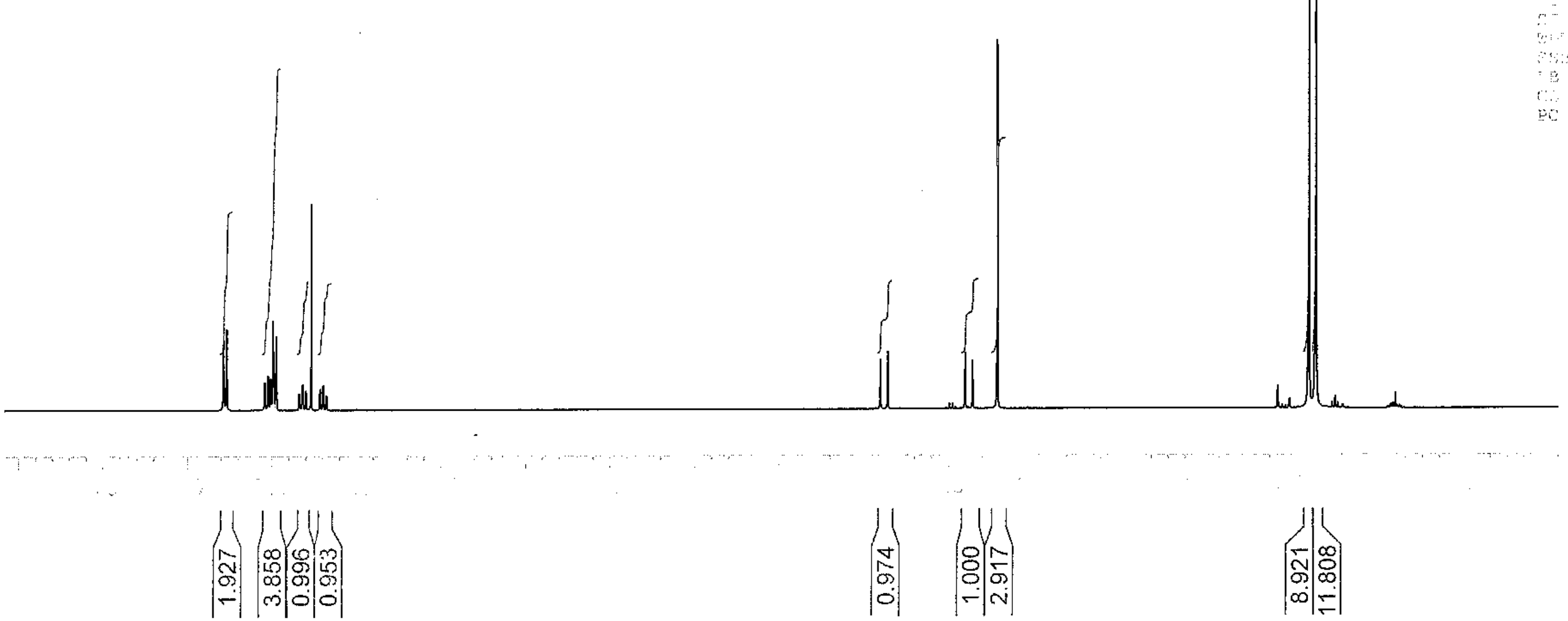

Name Sarah Harding
WBS R-00366-09-003
SLH074s
CSIRO Av400X_13C CDCl3 C:\\ har97c 31

BRUKER

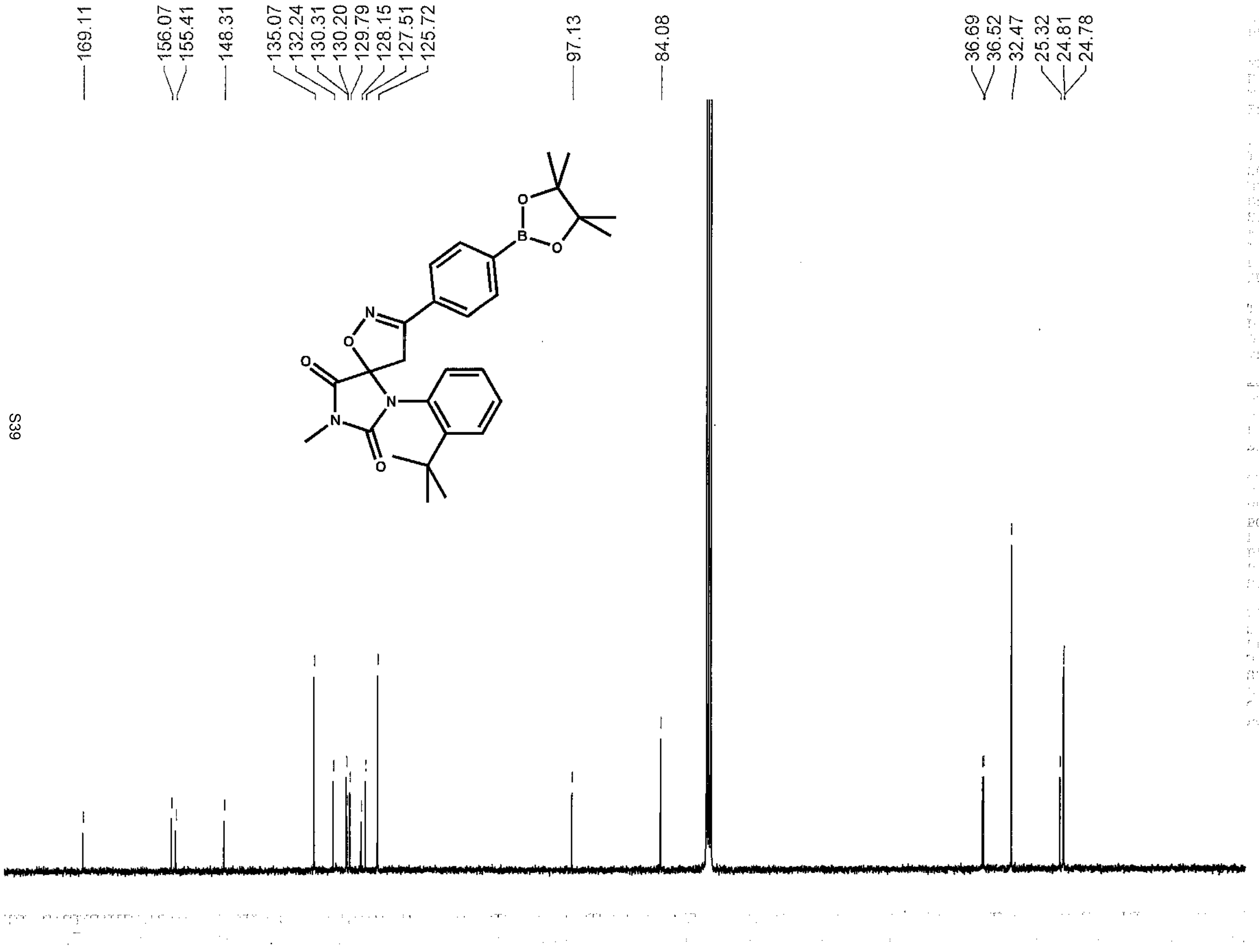

CSIRO

CSIRO Materials Science & Engineering
S.Harding

Accurate Mass Report
SLH074

Low Resolution EI Spectrum

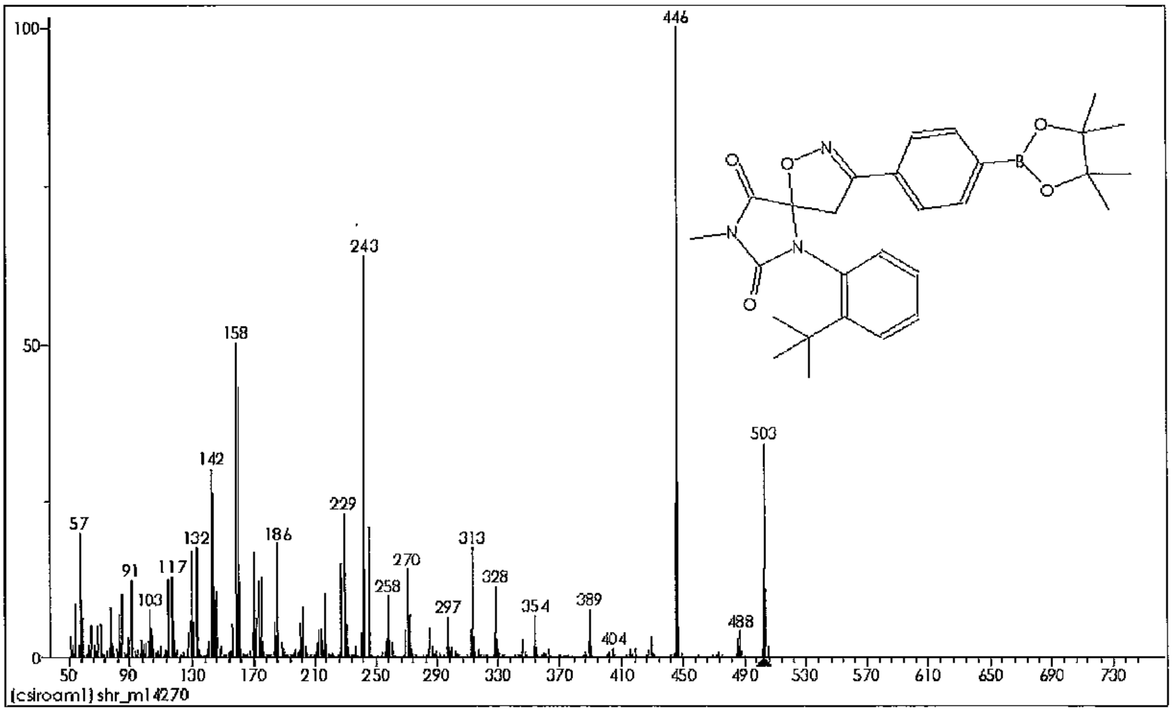

High Resolution EI Spectrum

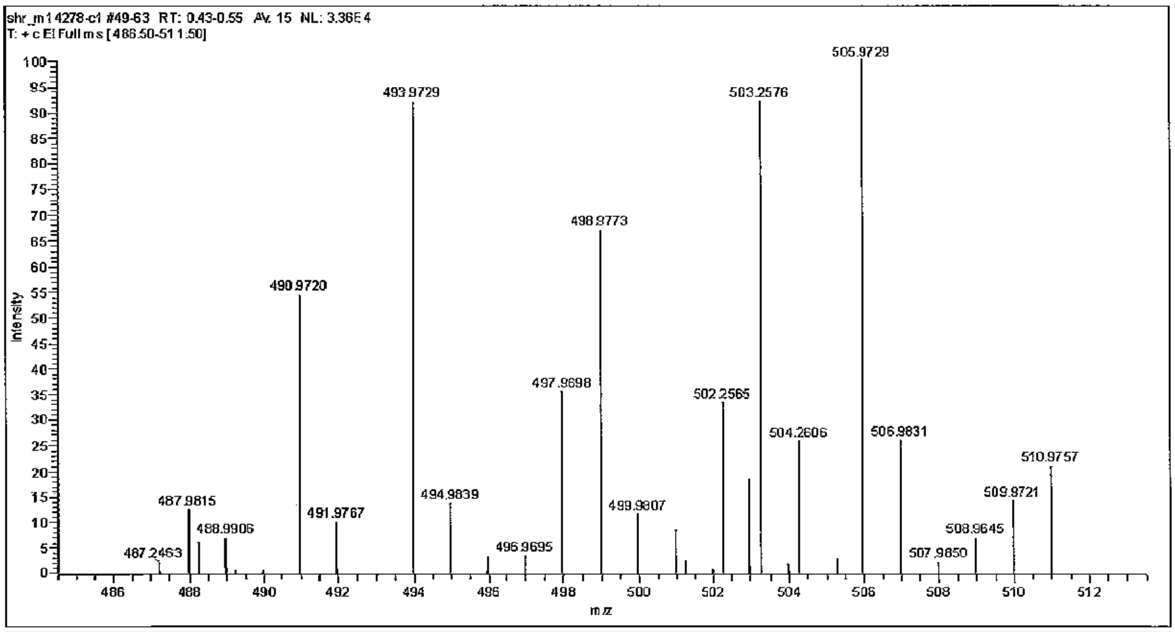

Supplement: File 1 — 1H and 13C NMR spectra, 2D spectra where required, and mass spectra for all compounds. [file Beilstein_J_Org_Chem-08-606-s001.pdf]
